# Three-Dimensional Super-Resolution in Eukaryotic Cells Using the Double-Helix Point Spread Function

**DOI:** 10.1016/j.bpj.2017.02.023

**Published:** 2017-04-11

**Authors:** Alexander R. Carr, Aleks Ponjavic, Srinjan Basu, James McColl, Ana Mafalda Santos, Simon Davis, Ernest D. Laue, David Klenerman, Steven F. Lee

**Affiliations:** 1 Department of Chemistry, University of Cambridge, Cambridge, United Kingdom; 2 Department of Biochemistry, University of Cambridge, Cambridge, United Kingdom; 3 Radcliffe Department of Clinical Medicine and Medical Research Council Human Immunology Unit, Weatherall Institute of Molecular Medicine, University of Oxford, Oxford, United Kingdom

## Abstract

Single-molecule localization microscopy, typically based on total internal reflection illumination, has taken our understanding of protein organization and dynamics in cells beyond the diffraction limit. However, biological systems exist in a complicated three-dimensional environment, which has required the development of new techniques, including the double-helix point spread function (DHPSF), to accurately visualize biological processes. The application of the DHPSF approach has so far been limited to the study of relatively small prokaryotic cells. By matching the refractive index of the objective lens immersion liquid to that of the sample media, we demonstrate DHPSF imaging of up to 15-*μ*m-thick whole eukaryotic cell volumes in three to five imaging planes. We illustrate the capabilities of the DHPSF by exploring large-scale membrane reorganization in human T cells after receptor triggering, and by using single-particle tracking to image several mammalian proteins, including membrane, cytoplasmic, and nuclear proteins in T cells and embryonic stem cells.

## Introduction

Single-molecule imaging (SMI) methods, including superresolution techniques such as photoactivated localization microscopy (PALM) (1) and (direct) stochastic optical reconstruction microscopy [(d)STORM] (2, 3, 4), are widely used to study the dynamics and organization of proteins within cells at a resolution below the diffraction limit (5). The majority of SMI has been carried out using total internal reflection fluorescence microscopy (TIRFM) (6), which is restricted to probing interfaces. Other excitation confinement techniques, such as light-sheet microscopy, are becoming widely used owing to their ability to image above the coverslip with high contrast (7), enabling intracellular imaging and minimizing surface-induced perturbations of protein dynamics (8). Like TIRFM, these methods typically employ two-dimensional (2D) imaging, which complicates the study of curved and irregular structures above the coverslip. This limitation has motivated the development of a number of three-dimensional (3D) SMI techniques, which allow for complete sampling of the protein distribution with high precision in all dimensions. Although many innovative techniques are available to access axial information (9, 10, 11), the most commonly implemented are astigmatism (12) and biplane (13). Both of these techniques are capable of achieving ∼10 nm lateral and ∼20 nm axial localization precision (14), but imaging is currently restricted to a diffraction-limited depth of field of ∼500 nm, which complicates the image analysis of large structures. The double-helix point spread function (DHPSF) (15, 16) is a widely used example of a rotating PSF whose intensity distributions rotate as they propagate along the optical axis, allowing access to a significantly larger depth of field (∼4 *μ*m) (17, 18). Whereas biplane and astigmatism have been shown to achieve a greater peak localization precision, one advantage of the DHPSF is that it maintains a more consistent localization precision in all dimensions across the entire depth of field (19). These factors make the DHPSF well suited to super-resolution imaging and single-particle tracking (SPT) in complex biological samples, for both imaging and live-cell single-molecule tracking experiments. In addition to PALM (1), the DHPSF is compatible with any other structurally resolved technique that relies on the localization of isolated emitters, including STORM (2, 3), dSTORM (4), and points accumulated for imaging nanoscale topography (PAINT) (20).

3D techniques have been used for SMI in a variety of prokaryotic (21, 22, 23) and eukaryotic (24, 25) cells. However, SMI in eukaryotic cells has proven to be particularly challenging due to their size, which significantly increases autofluorescence and out-of-focus fluorescence. This can be overcome by the use of selective plane illumination techniques (26), at the cost of increased experimental complexity. Scanning many separate image planes is also often required to capture entire cells, complicating the recovery of 3D information. On the other hand, 3D SMI techniques employing a large depth of field, such as DHPSF and multifocal microscopy (MFM) (27, 28), require significantly fewer imaging planes to cover thick samples, making these approaches more compatible with whole-cell imaging. However, spherical aberrations typically limit these techniques by degrading the image (29) above the coverslip, making very large volumes difficult to visualize. This effect can be minimized by matching the refractive index of the immersion liquid of the objective lens to the sample medium (30, 31), allowing for the DHPSF to be used above the coverslip surface (Figs. S1 and S2 in the Supporting Material).

Dynamic information can also be extracted from SMI by using SPT to determine the diffusion coefficient (32) and stoichiometries (33, 34) of protein complexes. SPT has been applied to a variety of eukaryotic cells (35). Most such studies have been conducted in two dimensions using TIRFM, although the use of 3D techniques is emerging, enabling SPT of cytoplasmic and nuclear proteins (36, 37). When tracking slower-moving membrane proteins, the narrow depth of field of most techniques is generally not a problem. However, faster-moving proteins are prone to escape the imaging volume, reducing the length of recorded trajectories and therefore potentially compromising the analysis. As a result, imaging of nuclear and cytoplasmic proteins has been problematic due to reduced depths of field when using high-numerical-aperture (high-NA) objective lenses. The large depth of field of the DHPSF combined with a water immersion objective lens allows for imaging of fast-moving proteins with long track lengths throughout cell volumes, making it ideally suited for SPT of a variety of target proteins.

Here, we demonstrate the versatility of the DHPSF in conjunction with a water immersion objective lens for both 3D whole-cell imaging and 3D SPT above coverslip surfaces, which can be performed in technically demanding areas of eukaryotic cells, including apical membranes and inside nuclei. Super-resolved localizations of a membrane protein covering ∼10- to 15-*μ*m-thick cell volumes reveal the large-scale receptor reorganization of the membrane of whole T cells.

We demonstrate that the technique can be applied to multiple cell types, including T cells and embryonic stem (ES) cells, enabling new biophysical studies on the 3D organization and dynamics of membrane, cytoplasmic, and nuclear proteins.

## Materials and Methods

### T cell culture

Jurkat T cells (ATCC TIB-152) were transduced via a lentiviral approach to stably express the *β*-chain of the T cell receptor (TCR), *ζ*-chain-associated protein kinase (Zap70) HaloTag-tagged proteins, and CD28-mEos3.2 chimeric protein. Jurkat T cells were grown in phenol red-free RPMI supplemented with 10% fetal calf serum (Sigma-Aldrich, Madison, WI), 1% HEPES buffer (Sigma-Aldrich), 1% sodium pyruvate (Sigma-Aldrich), 1% penicillin streptomycin (Sigma-Aldrich), and 2% glutamine (Sigma-Aldrich).

### T cell labeling

Before imaging, ∼10^6^ T cells expressing TCR-*β* or Zap70 HaloTag-tagged proteins were labeled with HaloLigand-tetramethylrhodamine (TMR; HaloTag TMR Ligand, Promega, Madison, WI) for 30 min at 37°C. The cells were then subjected to three washes in twice-filtered (0.22 *μ*m Millex-GP syringe filter unit, Millipore, Billerica, MA) phosphate-buffered saline (PBS, Life Technologies, Carlsbad, CA) followed by a 30 min incubation in T cell medium (involving centrifugation at 600 × *g*, 2 min). The cells were imaged live or were fixed in 4% paraformaldehyde (Sigma-Aldrich) and 0.2% glutaraldehyde (Sigma-Aldrich) for 60 min. Before imaging, the T cells were centrifuged and resuspended in 200 *μ*L of twice-filtered PBS.

### T cell coverslip coating

Glass slides (24 × 50 mm borosilicate, thickness No. 1, Brand, Wertheim, Germany) were cleaned with argon plasma (PDC-002, Harrick Plasma, Ithaca, NY) for 20 min and then coated with poly-L-lysine (molecular mass 150–300 kDa; P4832, Sigma-Aldrich) for 30 min when imaging fixed cells, or nonspecific immunoglobulin G (IgG; Jackson Immunoresearch Europe, Newmarket, UK) for 30 minutes, when imaging live cells. Then, 100 *μ*L of PBS was replaced and 20–100 *μ*L of cells in PBS was added and allowed to settle onto the surface.

To image fixed cells during immunological triggering events, argon-plasma-cleaned slides were coated with activating OKT3 (10 *μ*M/mL; S.D.’s group, Weatherall Institute for Molecular Medicine, University of Oxford) for 20 min. Live cells were added to the surface and allowed to settle for 5 or 10 min before the media was removed and replaced with 200 *μ*L of fixing solution for 60 min. The glass slides were then gently washed with PBS.

### ES cell culture

Mouse ES cell lines were cultured in standard serum and mouse leukemia inhibitory factor (mLIF) conditions (Glasgow minimum essential medium (MEM, Sigma-Aldrich) containing 100 mM 2-mercaptoethanol (Life Technologies), 1× MEM nonessential amino acids (Sigma-Aldrich), 2 mM L-glutamine (Invitrogen, Carlsbad, CA), 1 mM sodium pyruvate (Sigma-Aldrich), 10% fetal bovine serum (HyClone FBS, GE Healthcare, Vienna, Austria), and 10 ng/mL mLIF (provided by the Biochemistry Department, University of Cambridge)). They were passaged every 2 days by washing in PBS (Sigma-Aldrich), adding Trypsin-EDTA 0.25% (Invitrogen) to detach the cells, and then washing in media before replating in fresh media. To help the cells attach to the surface, the plates were incubated for 15 min at room temperature in PBS containing 0.1% gelatin (Sigma-Aldrich).

### ES cell labeling

Two days before imaging, cells were passaged onto 35 mm glass-bottom dishes (No. 1.0, MatTek, Ashland, MA) in phenol red-free serum and mLIF conditions. The cells were labeled with 0.5–5 nM HaloLigand-JF_549_ (38) for at least 15 min, followed by two washes in PBS, and then imaged in phenol red-free serum and mLIF conditions containing 5–10 mM Trolox. For cell fixation, cells were incubated in 1:1 methanol/ethanol for 6 min at −20°C and imaged in PBS containing 10 mM Trolox. The fixed cells were washed with PBS before imaging.

### DHPSF microscopy

**Figure 1:**
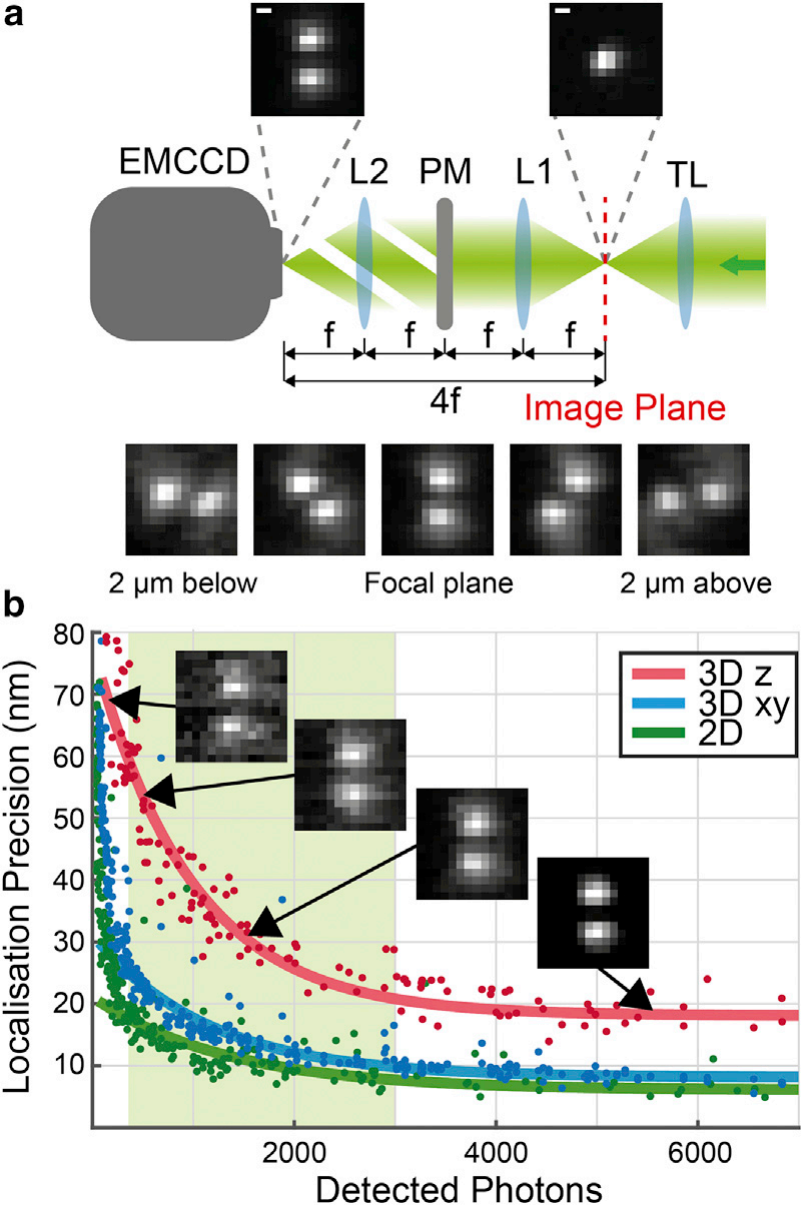
Emission path of DHPSF setup and empirically-determined localization precision of the DHPSF as a function of detected photons. (*a*) The DHPSF is implemented by the addition of a 4*f* system comprised of two lenses (L1 & L2) into the emission path of a fluorescence microscope with a DHPSF PM placed in the Fourier-transfer plane of the 4*f* system (*center of the two lenses*). The first plane of the 4*f* system is placed in the image plane of the microscope, relaying the emission signal onto an EMCCD placed a distance 4*f* away. Scale bars are 500 nm. (*b*) Measured lateral (xy) and axial (z) localization precision compared to a 2D fluorescence microscope as a function of detected photons with example DHPSFs. The highlighted region represents typical experimentally determined detected photon numbers when imaging fluorescent proteins and organic dyes. Localization precision was determined by imaging immobilized fluorescent beads.

A custom-made DHPSF microscope was built incorporating a 1.2 NA water immersion objective lens (Plan Apo VC 60×, Nikon, Tokyo, Japan), facilitating imaging above the coverslip surface. The DHPSF transformation was achieved by introducing additional optics into the emission path of a conventional fluorescence microscope (Eclipse Ti-U, Nikon) with the objective lens mounted onto a scanning piezo stage (P-726 PIFOC, PI, Karlsruhe, Germany) (Fig. S3). A 4*f* system of lenses placed at the image plane relayed the image onto an EMCCD detector (Evolve Delta 512, Photometrics, Tucson, AZ). A 580 nm optimized double-helix phase mask (PM) (DoubleHelix, Boulder, CO) placed in the Fourier plane of the 4*f* system performed the DHPSF transformation (Fig. 1 *a*). Excitation and activation illumination was provided by 561 nm (200 mW, Cobolt Jive 100, Cobolt, Solna, Sweden) and 405 nm (120 mW, iBeam smart-405-s, Toptica, Munich, Germany) lasers, respectively, that were circularly polarized, collimated, and focused to the back aperture of the objective lens. The fluorescence signal was then separated from the excitation beams into the emission path by a quad-band dichroic mirror (Di01-R405/488/561/635-25x36, Semrock, Rochester, NY) before being focused into the image plane by a tube lens. Finally, long-pass and band-pass filters (BLP02-561R-25 and FF01-580/14-25, respectively; Semrock) placed immediately before the camera isolated the fluorescence emission.

A range of exposure times were used to image the samples, with 30 ms used to image membrane proteins in SPT and 10–20 ms used to image the faster-diffusing cytoplasmic and nuclear proteins in SPT. An exposure time of 100 ms was used to image fixed cells in whole-cell scanning experiments. When imaging mEos3.2, a continuous 561 nm excitation beam at ∼1 kW/cm^2^ was used in conjunction with a shutter-pulsed (SC10 shutter controller, ThorLabs, Newton, NJ) 405 nm photoconversion beam at 5 W/cm^2^, which photoconverted mEos3.2 molecules across all planes of the sample. When imaging TMR, only a continuous 561 nm excitation beam at ∼940 W/cm^2^ was used. Specific case-by-case photon values and corresponding localization precisions are included for all presented experiments in Fig. S4.

### Fluorescent bead preparation/DHPSF calibration

To calibrate the angle between the lobes in the DHPSF as a function of axial position, 100 *μ*L of an ∼3.6 × 10^8^ particles/mL solution of fluorescent beads (0.1 *μ*m, TetraSpeck microspheres, ThermoFisher, Waltham, MA) in PBS was imaged on a surface. Microscope slides cleaned with argon plasma (PDC-002, Harrick Plasma) were coated with poly-L-lysine for 30 min and washed with PBS before adding the diluted beads. After 5 min at room temperature, the slides were washed in PBS and imaged using the DHPSF instrument. The piezo stage was used to scan the objective lens axially through the sample in 50 nm steps over 4 *μ*m, recording 10 30-ms exposures at each step.

### Determination of localization precision

The DHPSF microscope calibration slides were imaged for 2000 frames with 30 ms exposure, with and without the inclusion of the PM in the imaging path. A range of excitation powers was used such that the emission signal of the beads covered the dynamic range of the EMCCD. The localization precision was determined using a previously described method (23). Beads were localized using PeakFit (GDSC SMLM single-molecule plugins (39)) for 2D images and easy-DHPSF (40) for DHPSF microscopy images, and the distribution of localized positions for each bead was analyzed to determine the localization precision of the instrument as a whole. An exponential decay was fit to each data set to guide the eye.

Lateral and axial localization precision was measured at ∼10–25 nm and ∼20–60 nm, respectively, in typical measured photon ranges (Fig. S4) when imaging fluorescent proteins or organic dyes (Fig. 1 *b*). Typical photon values for each experiment are listed in Table S1.

### Whole-cell scanning

To image whole cells, we scanned the focal plane axially through the cells in 3 *μ*m steps via the piezo-mounted objective, acquiring 100 images at each position before moving to the next position. This whole process was repeated until no more localizations were observed, typically 50 times (∼20,000 frames). Between 1000 and 5000 localizations were collected for each cell depending on the experiment. By offsetting the recorded localizations from different imaging planes, a super-resolution whole-cell map of the tagged protein was produced. Typically, for a 10–15 *μ*m cell, three to five overlapping planes were collected. The collection efficiency profile of the objective lens causes a reduction in recorded localization density at the periphery of the depth of field (Fig. S5, *a* and *b*). We have experimentally determined that successive imaging planes should be offset by ∼3 *μ*m or 75% of the working depth of field to achieve the flattest localization density across multiple imaging planes (Fig. S5 *c*). We determined the sample drift and lateral offset between imaging planes by imaging fluorescent beads (Figs. S6 and S7), and found them to be small compared with other error sources in the presented quantitative analysis (a maximum of 89 ± 8 nm and 45.5 ± 22 nm).

### Mesh fitting

Using a standard method and functions included in Meshlab open source software, (http://meshlab.sourceforge.net), the 3D localization data were converted into an object mesh. For each point, 50–200 neighbors (depending on the localization density) were considered to estimate a normal. These points and their normals were then used to build a surface using the Poisson surface reconstruction approach (41, 42), which solves an approximate indicator function of the object by fitting its gradient to the input normal. Finally, meshes were uniformly sampled, creating an even distribution of vertices (see Fig. S8 for detailed instructions).

### Cell-specific complete spatial randomness model

An algorithm to create a complete spatial randomness (CSR) model from a given cell mesh was written in MATLAB (The MathWorks, Natick, MA). A number of vertices equal to the number of unique proteins localized from the cell were sampled randomly from the mesh. The select vertices were then displaced by a random distance between −250 and 250 nm in all three dimensions, representing the approximate precision of the fitted mesh (∼500 nm). An additional displacement was sampled from a normal distribution in all dimensions to represent the reconstruction error. This displacement was centered at the recorded localization precisions for the number of photons detected in the corresponding experiment. These vertices could then be used as model localization data in quantitative analysis, representing the position of proteins on the outer cell membrane.

### Interprotein distance analysis

An algorithm to analyze the interprotein distances of 3D localization data was written in MATLAB. First, duplicate localizations originating from repeat localizations of the same fluorophore were removed by filtering for nearby recorded events in space (<500 nm) and time (<1 s). For each remaining localization, the distance to all other localizations was calculated and used to create a histogram. The peak value of this histogram, representing the median interprotein distance, was then recorded for each localization.

The effect of cell morphology was corrected for by finding the deviation from the cell-specific CSR model localizations. The interprotein distance analysis was conducted on 1000 instances of model localization data for each cell, taking the mean and the 5th and 95th percentiles to create error limits. The mean deviation of the true interprotein distance from the model interprotein distance provides information about how well the CSR model fits as a whole. The distribution of median interprotein distance as a function of axial position was also investigated by taking the mean value in 500 nm slices axially.

### Diffusion analysis

An algorithm to perform mean-square displacement (MSD) analysis using 3D localization data was written in MATLAB by extending a previously published method (43) to three dimensions. The performance of the algorithm was benchmarked using simulated data and by comparing the results from 2D data with those produced by the parent algorithm (Fig. S9).

To determine the diffusion coefficient of imaged proteins, an ensemble MSD curve was created by taking the mean MSD of the trajectory data at each time interval given by

(1) $$MSD\left(n\Delta t\right)=\frac{1}{l-n}\sum_{i=1}^{l-n}{\left[x\left(i+n\right)-x\left(i\right)\right]}^{2}+{\left[y\left(i+n\right)-y\left(i\right)\right]}^{2}+{\left[z\left(i+n\right)-z\left(i\right)\right]}^{2},$$

where Δ*t* denotes the time step between frames, *l* the trajectory length, and *x*(*i*), *y*(*i*), and *z*(*i*) the position of the particle in frame *i*. The gradient of a linear fit to the first four points of the curve was divided by twice the number of dimensions of diffusion (two for membrane-bound diffusion and three for intracellular diffusion) to give the diffusion coefficient (32, 44), with the error in fit determining the calculated error in the mean diffusion coefficient:

(2) MSD(nΔt)=qDnΔt+C,

where *q* is given by twice the number of dimensions of diffusion, *D* denotes the diffusion coefficient, and *C* is the offset.

To separate bound and unbound trajectories, MSD plots were created for individual trajectories and fit to a straight line. The R-squared value of this fit was used to threshold bound and unbound trajectories. An R-squared value of 0.85 was determined by quantitative analysis of simulated data using empirically determined parameters, including localization precision, and then verified with experimental data (Fig. S10).

## Results

### DHPSF whole-cell super-resolution imaging

**Figure 2:**
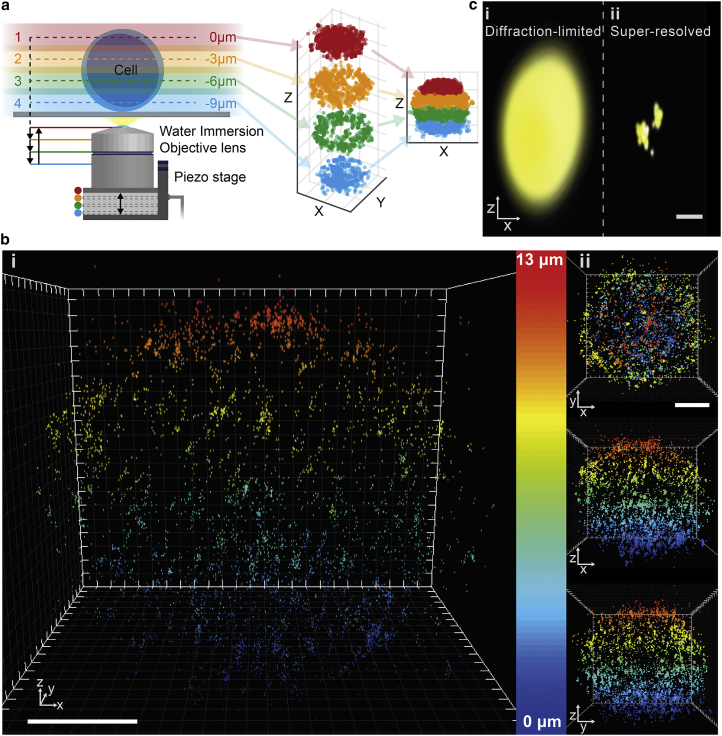
Super-resolved whole-cell reconstruction of a T cell using the DHPSF. (*a*) Experimental procedure for whole-cell scanning. A 60x 1.2NA water immersion objective lens was mounted on a piezo stage. Localizations were recorded from 3–5 overlapping imaging planes throughout the cell volume before being combined to form the final whole-cell image. (*b*) Super-resolved whole-cell reconstruction of CD28 membrane protein on a Jurkat T cell (4810 localisations). Jurkat cells expressing CD28-mEos3.2 were imaged with the DHPSF. Localizations are color coded by axial height from the coverslip and rendered with isotropic 3D Gaussians with size determined by experimentally measured localization precision for ∼350 photons detected (Figure S3) (*b i*). (*b ii*) Top-down and side-on views of (*b i*) rendered with 100 nm localization precision for visibility. Scale bars are 4 μm. (*b*) and (*c*) were plotted using ViSP localization visualization software (71). (*c*) Comparison between diffraction-limited (*c i*) and super-resolved (25 nm lateral precision and 50 nm axial precision) (*c ii*) rendering of two clusters of localizations originating from two individual TCR proteins separated by ∼300 nm. Scale bar is 300 nm. Jurkat cells expressing TCR-HaloTag were labeled with TMR-HaloTag ligand and imaged with the DHPSF.

Whole-cell super-resolution imaging was achieved by scanning a water immersion objective lens in three to five axial planes (Fig. 2 *a*; Materials and Methods). This allowed DHPSF imaging of a variety of T cells stably expressing fluorescently tagged forms of two membrane receptors with multiple labeling strategies, including CD28-mEos3.2 in a PALM mode and low-concentration labeling of TCR-HaloTag-TMR. As expected, fixed Jurkat cells appeared predominantly spherical (Fig. 2 *b*), with no large systematic differences in shape for all cells imaged (see Fig. S11 for other examples). In addition, the localization precision (∼10–25 nm laterally and ∼20–60 nm axially; Fig. 1 *b*) allowed for receptors to be resolved well below the diffraction limit (Fig. 2 *c*). The distribution of nearest-neighbor distances between CD28 molecules revealed that 27% were too close to be resolved by standard confocal microscopy (<250 nm apart) and 63% were closer than 500 nm apart (Fig. S12).

### Visualizing and quantifying large-scale reorganization over whole cells

**Figure 3:**
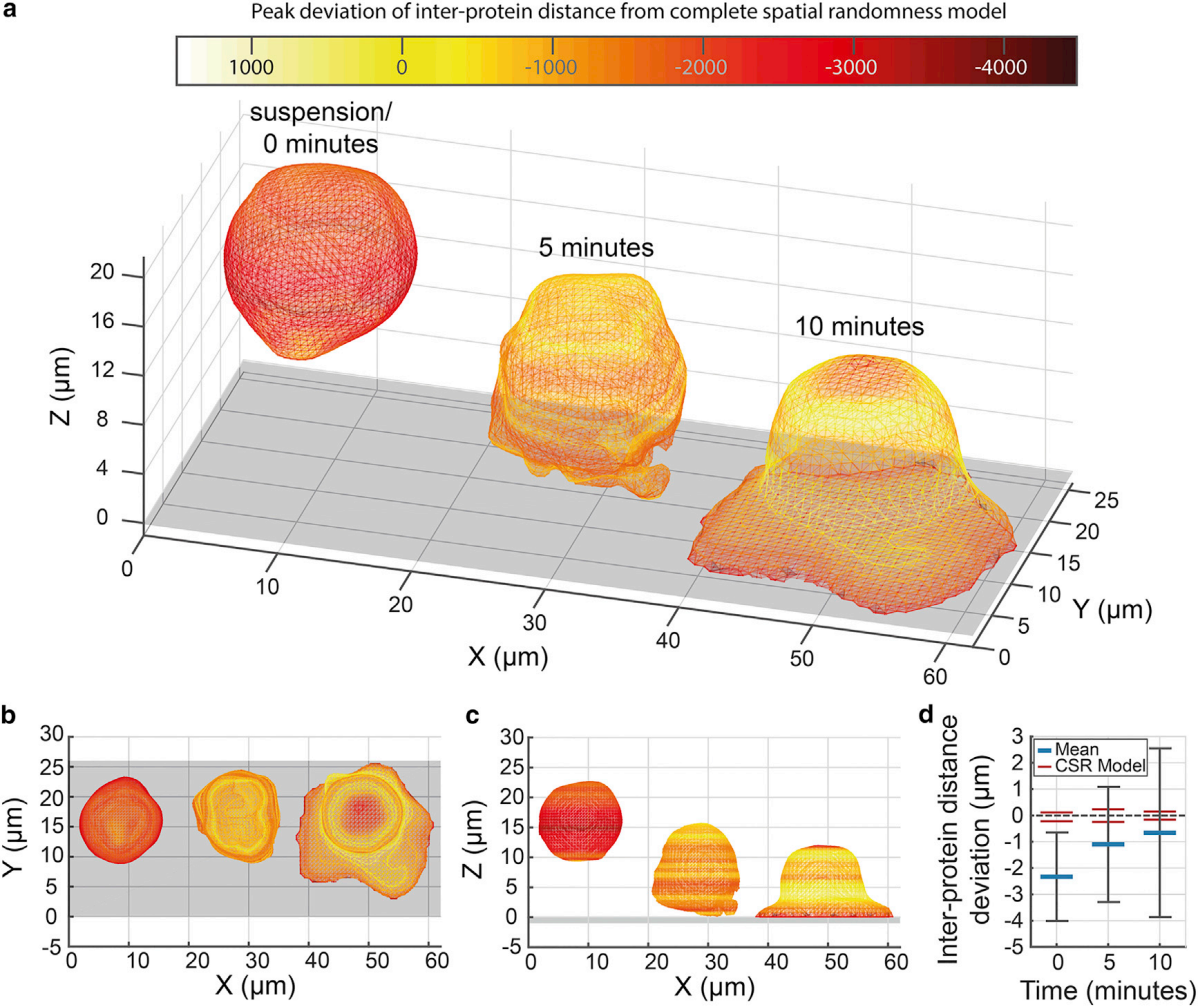
Visualization of large-scale reorganization of the outer membrane of Jurkat T cells on activating coated surfaces by mesh fitting to 3D localization data. (*a*) Meshes were fit to DHPSF whole-cell TCR localization data of three Jurkat T cells tagged with HaloTag-TMR. Cells were fixed in solution (*left*, 1149 localizations), 5 minutes (*center*, 2495 localizations), or 10 minutes (*right*, 2397 localisations) after being dropped onto an activating OKT3 coated surface. Large-scale reorganization of the outer membrane caused by immune-response triggering and the formation of an immunological synapse is visualized at different stages. Cells are colored axially by deviation of inter-protein distance from a complete spatial randomness model of a membrane-bound protein with yellow, which indicates no deviation from model system, and red/black, which indicates a reduction in peak inter-protein distance compared to model system. The flat grey surface represents the coverslip of the experiment. The cell fixed in solution (*left*) has been rendered away from the coverslip to emphasize the resting state. (*b*–*c*) Top-down and side-on views of (*a*). (*d*) Mean inter-protein distance deviation of each cell shown in (*a*) from a complete spatial randomness model of a membrane-bound protein.

T cells are known to undergo large-scale spatial reorganizations when activated, presumably to maximize contact with the target antigen-presenting cell (45). A key protein in this process is the TCR, which binds to major histocompatibility proteins that are expressed by the antigen-presenting cell and present antigenic peptides, resulting in T cell activation (46, 47, 48). DHPSF imaging was used to examine and quantify the distribution of the TCR on individual T cells. By fitting meshes to these sparse localization data, the mean position of the plasma membrane of each T cell was extracted and used to visualize large-scale morphological changes at three distinct time points during activation (Fig. 3, *a*–*c*). As the fitted meshes served to report the average position of the outer membrane, some proteins were localized >1 *μ*m away from the mesh, corresponding to large pseudopodia and internal stores.

Substantial differences in morphology were observed between resting cells fixed in suspension versus those fixed after contacting an activating antibody (OKT3)-coated surface. In suspension, the cells were largely spherical, whereas cells that had been in contact with an activating surface for 5 min exhibited flattening and extension of the basal membrane close to the surface. Much larger membrane spreading was evident for cells contacting the surface for 10 min.

For the three time points, the vertex densities in the final cell meshes were 5.1, 4.9, and 2.8 vertices/*μ*m^2^ with 0.4, 0.7, and 0.8 localizations per vertex, respectively.

Furthermore, it is possible to decouple protein reorganization from morphological changes by using quantitative analysis (Fig. S13). Initially, the mean interprotein distance distribution of the TCR was computed across whole cells, and by creating cell-specific volumes, this distribution was compared with a model of protein positioned randomly on the outer membrane (see Materials and Methods for a detailed discussion). The interprotein distance distribution of the TCR in the resting cell was found to be significantly different from that predicted by the membrane-bound CSR model. In contrast, the TCR distribution in the activated cells (after 5 and 10 min) overlapped substantially with the analogous CSR models (Fig. 3 *d*). Deviation in the inter-TCR distance was not observed to change significantly with axial depth for any of the cells analyzed (Fig. S13, *e*–*g*).

### DHPSF-SPT on the apical surface and in the cytoplasm of T cells

**Figure 4:**
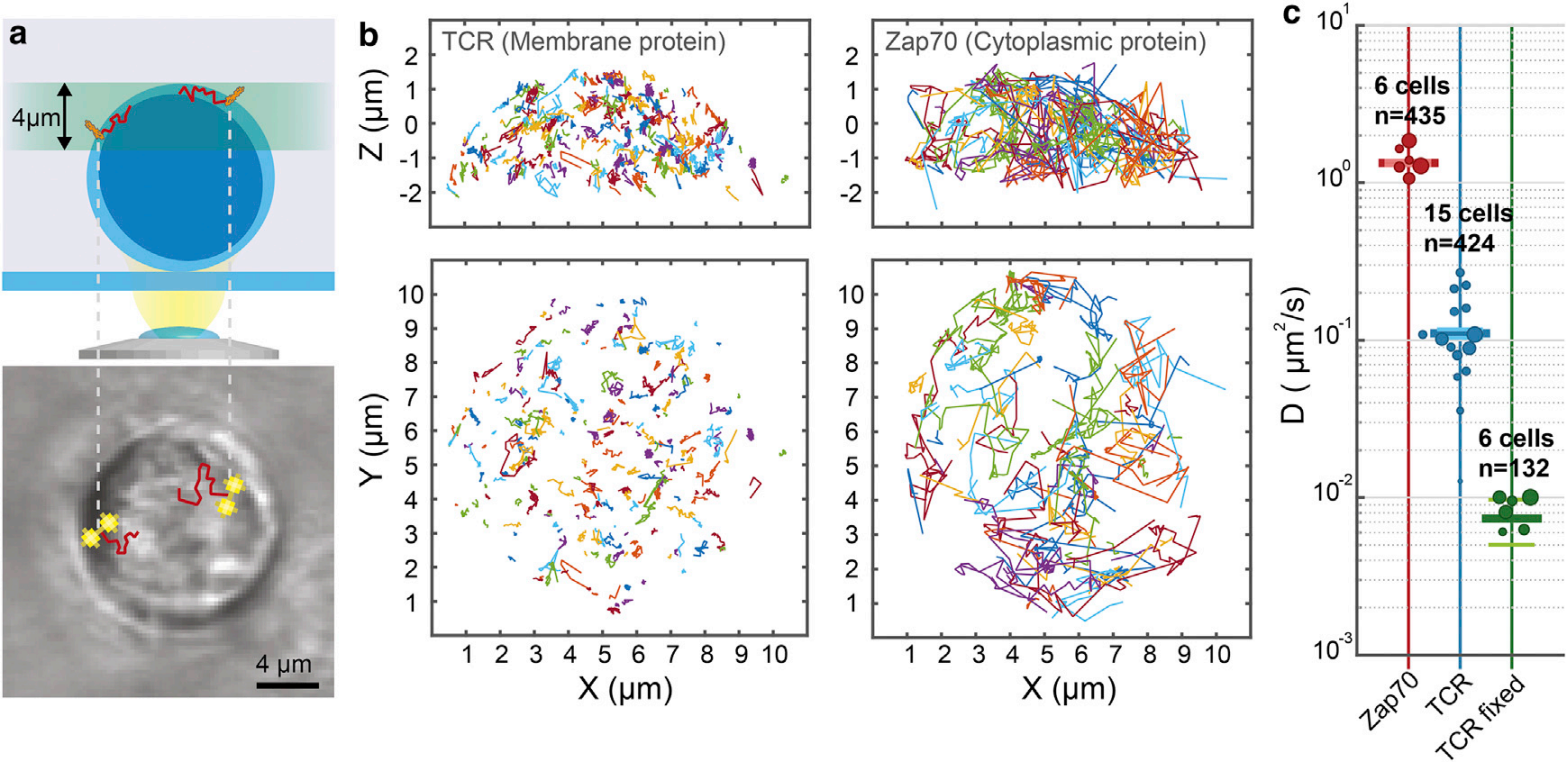
SPT of membrane and cytoplasmic proteins using the DHPSF in live human T cells. (*a*) Experimental procedure for SPT at the apical surface using the DHPSF. Jurkat T cells were labeled with HaloTag-TMR and dropped onto passivating-coated coverlips. A 60x water immersion objective was focused at the apical surface to image the membrane protein TCR or the cytoplasmic protein Zap70. TCR was also imaged in fixed cells. (*b*) Representative side-on (*top*) and top-down (*bottom*) views of trajectories of unfixed TCR (*left*) and Zap70 (*right*) proteins at the apical surface. The difference in speed between the two proteins can be clearly seen in the length of trajectories. (*c*) Mean diffusion coefficient for Zap70 and TCR live and fixed determined by MSD analysis (*horizontal bars*) with cell-to-cell variation (*circles*) and total number of trajectories. The size of the circles is proportional to the number of tracks obtained from the cell. Small circles represent cells with fewer trajectories while large circles represent cells with many trajectories. The number of tracks ranges from ∼30-140 per cell for Zap70 and ∼5-140 for TCR.

Membrane-bound and cytoplasmic T cell proteins were imaged on the top ∼4 *μ*m of Jurkat T cells attached to IgG-coated coverslips (Fig. 4 *a*). The membrane-associated TCR, labeled with HaloTag-TMR, was imaged as it diffused over the nanostructured outer membrane of live Jurkat T cells, and the intracellular protein Zap70, also labeled with HaloTag-TMR, was imaged inside live Jurkat T cells (Fig. 4 *b*). MSD analysis revealed that the TCR had a mean diffusion coefficient of 0.110 ± 0.007 *μ*m^2^/s (424 trajectories, 15 cells), whereas Zap70 had a significantly faster mean diffusion coefficient of 1.34 ± 0.04 *μ*m^2^/s (435 trajectories, six cells) (Fig. 4 *c*). The precision in diffusion coefficient measurements was estimated by MSD analysis of static TCR in fixed cells and found to be 0.008 ± 0.002 *μ*m^2^/s (132 trajectories, six cells), agreeing well with the observed errors in live-cell TCR experiments.

The MSD plot for the TCR fits well to a straight line, indicating free diffusion, whereas the plot for Zap70 drops below the straight line fitted to the first four points, indicating a degree of confinement (32) (Fig. S14).

### DHPSF-SPT in the nucleus of ES cells

**Figure 5:**
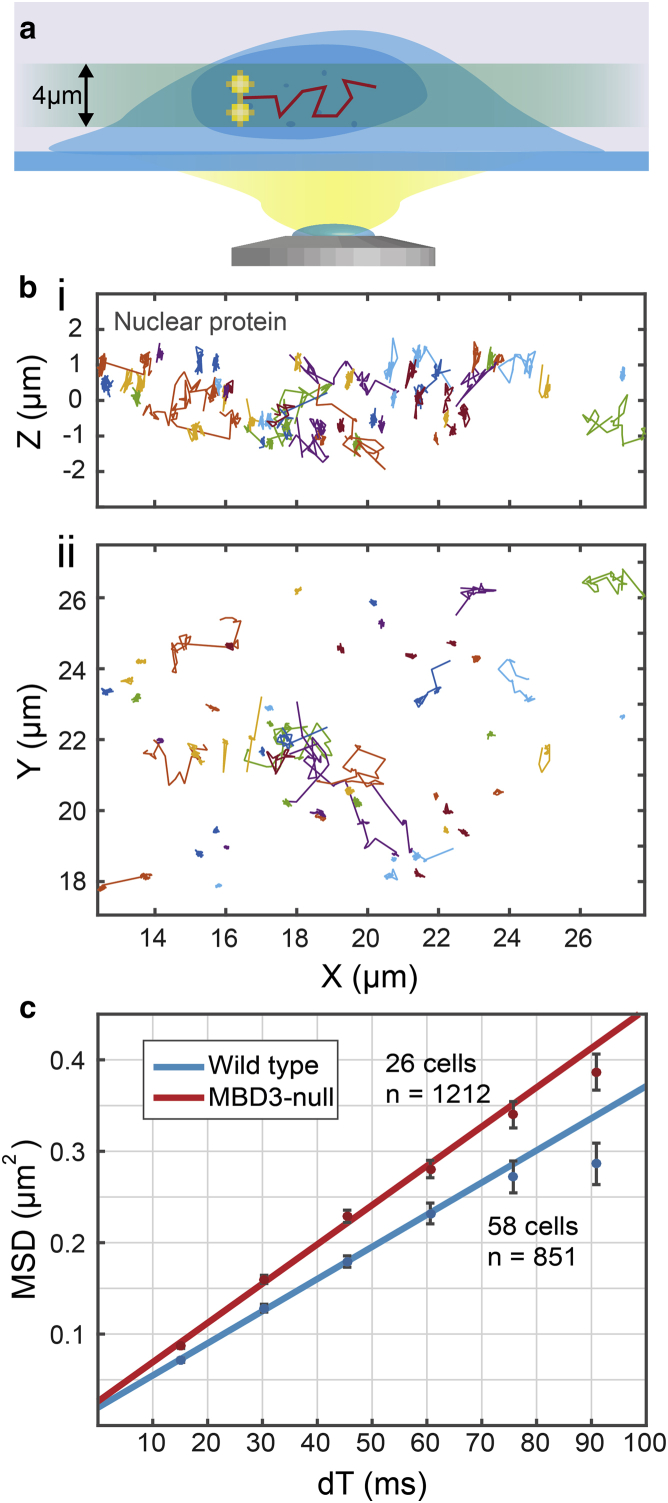
SPT of CHD4 nuclear protein using the DHPSF in live mouse ES cells. (*a*) Experimental procedure for SPT in the nucleus of ES cells using the DHPSF. CHD4 was labelled with HaloTag-JF549 in WT cells and MBD3 null mutants and imaged in a ∼4 μm-thick plane above the coverslip, within the nucleus of ES cells. (*b*) Side-on (*i*) and top-down (*ii*) view of CHD4 trajectories from a single ES nucleus. The bound and unbound states of CHD4 can clearly be seen. (*c*) MSD plot for the unbound trajectories in WT cells and MBD3 null cells with linear fits to the first 4 points.

We investigated the chromatin remodeler CHD4, a nuclear protein that is known to play a critical role in ES cell pluripotency as part of the larger nucleosome remodeling and deacetylase complex, as well as in the DNA damage response and cell-cycle regulation (49). CHD4 was previously shown to occupy two diffusion states in near-equal ratios: 1) bound to chromatin and 2) a fast-moving state (50). It was reported that removal of the nuclear protein Methyl-CpG Binding Domain Protein 3 (MBD3) resulted in an ∼25% increase in the diffusion coefficient of the fast-moving population of CHD4 compared with wild-type (WT) cells (50). We used the DHPSF to track nuclear CHD4 tagged with HaloTag-JF_549_ in WT mouse ES cells and MBD3 null mouse ES cells (Fig. 5, *a* and *b*).

The mean diffusion coefficient of unbound CHD4 was determined to be 0.60 ± 0.01 *μ*m^2^/s in wild-type cells (851 trajectories, 58 cells) and 0.75 ± 0.03 *μ*m^2^/s in MBD3 null cells (1212 trajectories, 26 cells), agreeing with previous observations (50). Both MSD plots exhibited linearity for the first four points (Fig. 5 *c*), as observed for measurements of Zap70 diffusion.

## Discussion

### Whole-cell imaging with the DHPSF

The DHPSF facilitates the probing of complex structures in the intricate 3D environment of the cell nucleus and the apical cell surface. Due to its large depth of field, super-resolution, whole-cell images of ∼10- to 15-*μ*m-thick samples can be collected in as few as three to five imaging planes. Reducing the number of required imaging planes is beneficial not only because it reduces experimental complexity but also because it allows one to image a greater fraction of emitting fluorophores simultaneously when using excitation geometries that excite all axial planes (i.e., epifluorescence or highly inclined and laminated optical sheet) (51). This is highly relevant when imaging low-density samples such as weakly expressing proteins (e.g., the TCR). Comparable sectioning of whole cells has previously been demonstrated with other 3D imaging methods (28, 52).

### Visualizing morphology and quantifying protein reorganization

Fitting meshes to localization data facilitates the visualization of larger morphological changes in the outer membrane of cells as compared with rendering individual localizations, as the eye is no longer drawn to the variations in localization density that are intrinsic to pointillism-based imaging techniques. The meshes report the weighted position of outer localizations, emphasizing the cell membrane and reducing the visual impact of localizations arising from intracellular sources (53).

The precision of the fitted mesh is primarily determined by the sampling density of the localization data. However, even at relatively low sampling densities, large-scale membrane reorganization can be observed and quantitatively analyzed. Mesh fitting is also compatible with super-resolution membrane imaging and visualization, providing adequate sampling density. This is possible in imaging strategies with large localization numbers, such as PAINT (20), which can achieve 10^6^–10^9^ localizations from a single cell with sufficiently long imaging periods (54).

The reorganization shown in Fig. 3 is likely to be an active process as the T cell attempts to form an immunological synapse (55, 56). The peak interprotein distance of the TCR in the resting cell was found to be smaller than predicted by the corresponding membrane-only model system, implying that a significant fraction of the TCR was localized intracellularly. However, the distribution of peak inter-TCR distance in activated cells overlapped with their model systems, indicating a reduction in the intracellular fraction. This observation is supported by an additional analysis of membrane-bound and cytoplasmic TCR fractions, in which TCR molecules that localized within the mesh by >1 *μ*m were considered as intracellular (Fig. S15). In the resting cell, 30% of TCR molecules were localized in the cytoplasm, whereas 17% and 13% of TCR molecules were localized in the cytoplasm in both activated cells (5 and 10 min, respectively). In the resting cell, 65% of TCR molecules were localized within 1 *μ*m of the mesh, whereas 72% and 74% were localized within 1 *μ*m of the mesh in both activated cells (5 and 10 min, respectively). These data are consistent with the notion that immunological stimulation causes intracellular TCRs to be recruited to the outer membrane in Jurkat T cells. One possible mechanism for this is that the TCR is stored in microvesicles in the resting cell, and T cell signaling results in the release of these stored TCRs at the cell surface (57, 58). No axial dependence of the distribution of interprotein distance of the TCR was seen, implying that the changes occur across the entire cell rather than being part of a directed process. This analysis nevertheless demonstrates that the DHPSF can resolve relatively small redistributions of molecules across whole cells, such as the transfer of proteins from the cytosol to the cell surface. In the case of low-expressing proteins (e.g., the TCR), a small number of molecules moving from the cell cytoplasm to the cell membrane could significantly affect the overall distribution.

### SPT of proteins on the apical surface and in the cytoplasm of T cells

The TCR diffusion rates are significantly higher than suggested by literature values obtained from the apical surface of Jurkat T cells, which James et al. (59) measured to be 0.06 ± 0.01 *μ*m^2^/s using fluorescence correlation spectroscopy. No value for cytoplasmic Zap70 could be found for comparison.

Analysis of a 2D *x-y* projection of the 3D data produces a diffusion coefficient of 0.064 ± 0.004 *μ*m^2^/s for the TCR (Fig. S16 *d*), in good agreement with the literature values. This difference in extracted diffusion coefficient, from three dimensions to two dimensions, was found to be greater than predicted by simulating trajectories on the apical surface of a spherical membrane alone (a factor of 1.72 ± 0.22 difference was seen compared with the predicted factor of 1.34 ± 0.02; Fig. S16 *b*). This is due to trajectories displaying a radial component as well as the angular component expected from a spherical surface (highlighted in Fig. S16 *c*). This is most likely caused by pseudopodia or surface ruffling present on the plasma membrane (60, 61). A previous analysis of T cell morphology using electron microscopy determined a roughness factor of 1.8 (62), which has since been used to correct for 3D effects in T cell membrane protein studies (63, 64). This represents a case where 3D SPT is essential for accurately studying protein dynamics in complex 3D environments.

### SPT of proteins in the nucleus of ES cells

An increase in the diffusion coefficient of CHD4 in the absence of MBD3 compared with wild-type cells was seen, confirming observations made in a previous 2D study (50). The earlier study of CHD4 was not able to utilize MSD analysis because the fast diffusion led to short recorded trajectories, primarily due to the small nominal focal plane (∼500 nm) in conventional 2D SPT. Due to the large depth of field afforded by the DHPSF, longer trajectories could be recorded and thus MSD analysis could show that the fast fraction of CHD4 is largely freely diffusing within the nucleus. This example confirms that the DHPSF can be used to study proteins exhibiting a variety of diffusion states across a large dynamic range of diffusion coefficients.

### Advantages and disadvantages of the DHPSF

As we have shown, the DHPSF can be used to perform 3D SPT in two key areas in which 2D methods typically perform poorly: 1) on apical cell surfaces and 2) in the nuclei of living cells. Even when imaging membrane phenomena on glass surfaces, the presence of membrane ruffles could make a significant axial contribution to observable behaviors (60, 65).

The increased depth of field of the DHPSF (∼4 *μ*m) compared with the majority of 3D single-molecule SPT techniques (e.g., astigmatism and biplane) also has the advantage of capturing extended trajectories, as the fluorescent molecules are less likely to leave the imaging volume. This allows for more robust quantitation in standard analysis tools such as MSD, making the DHPSF well suited for tracking a large range of proteins throughout the cell, particularly fast-moving cytoplasmic and nuclear proteins. Other 3D tracking techniques, such as the emerging MFM, have demonstrated a similar depth of field and axial localization precision (27).

The primary disadvantage of the DHPSF is the increased size of the PSF versus other methods. As with the majority of 3D localization techniques, the PSF occupies more area on the detector in the conjugate image plane. In the case of the DHPSF, the area is ∼5-fold larger compared with the analogous 2D experiment. Consequently, labeling concentrations should be reduced accordingly, which increases the experimental acquisition time. Secondary issues are the reduction in signal originating from the DHPSF and inefficiencies in the DHPSF PM and additional optics. As the photons comprising the standard 2D PSF are split into two separately localized lobes, the apparent fluorophore intensity is reduced (∼43% of 2D PSF photons are observed in each lobe). Furthermore, the use of a low-NA water immersion objective lens not only reduces collection efficiency compared with oil immersion objectives but also causes background signal to be collected from a larger volume. Therefore, samples with a low signal/noise ratio can be more difficult to image, requiring careful experimental optimization and design. Fluorescence-background-reduction techniques such as selective plane illumination could be employed to increase signal/noise ratios (66) and offset this effect. The large depth of field of DHPSF is ideally suited to implementing simple light-sheet systems that typically have a thickness of a few microns (67). Light-sheet microscopy has been combined with the DHPSF to increase signal/noise ratios (68), but this approach has not yet been applied to cell imaging.

## Conclusions

3D super-resolution imaging and SPT have become vital biophysical tools for understanding the organization and dynamics of membrane and intracellular proteins. Using the DHPSF, we were able to super-resolve the position of membrane proteins across whole cells as well as provide quantitative information about their distribution. This approach could potentially be used to elucidate biophysical phenomena related to the nanoscale organization of proteins, such as the recruitment of intracellular TCR to the outer membrane, the role played by TCR clustering in the adaptive immune response (69), and nuclear protein organization in ES cells (70). Large-scale spatial reorganization could be quantified at the single-molecule level and visualized by fitting the superresolved localizations of membrane proteins with a surface mesh. The diffusion coefficients measured and observations made using the DHPSF for SPT agree well with previously published findings. Ultimately, we demonstrate the first, to our knowledge, implementation of the DHPSF in eukaryotic cells, and show that the technique is compatible with both super-resolution imaging and SPT for studying protein dynamics with super-localise spatial resolution in all three dimensions (71).

## Author Contributions

S.F.L. and A.R.C. designed the experimental plan. A.R.C. performed experiments, analyzed the data, and wrote the article. A.P. provided code for simulating tracks on a spherical membrane. S.B. provided and labeled ES cells. J.M. maintained and labeled Jurkat T cells. A.M.S. and S.D.'s lab provided all T cell lines. All authors contributed to the writing of the manuscript and helpful discussion.

## Supporting Material

Document S1. Supporting Materials and Methods, Figs. S1–S16, and Table S1

Data S1. Sample Localization and Mesh Data

Document S2. Article plus Supporting Material

## References

[1] Betzig E., Patterson G.H., Hess H.F. (2006). Imaging intracellular fluorescent proteins at nanometer resolution. Science.

[2] Rust M.J., Bates M., Zhuang X. (2006). Sub-diffraction-limit imaging by stochastic optical reconstruction microscopy (STORM). Nat. Methods.

[3] Bates M., Huang B., Zhuang X. (2007). S-Multicolor super-resolution imaging with photo-switchable fluorescent probes. Science.

[4] Heilemann M., van de Linde S., Sauer M. (2008). Subdiffraction-resolution fluorescence imaging with conventional fluorescent probes. Angew. Chem. Int. Ed. Engl..

[5] Lord S.J., Lee H.L., Moerner W.E. (2010). Single-molecule spectroscopy and imaging of biomolecules in living cells. Anal. Chem..

[6] Mattheyses A.L., Simon S.M., Rappoport J.Z. (2010). Imaging with total internal reflection fluorescence microscopy for the cell biologist. J. Cell Sci..

[7] Hu Y.S., Zimmerley M., Cang H. (2014). Single-molecule super-resolution light-sheet microscopy. ChemPhysChem.

[8] James J.R., McColl J., Davis S.J. (2011). The T cell receptor triggering apparatus is composed of monovalent or monomeric proteins. J. Biol. Chem..

[9] Jia S., Vaughan J.C., Zhuang X. (2014). Isotropic 3D super-resolution imaging with a self-bending point spread function. Nat. Photonics.

[10] Yang Z., Prokopas M., Dholakia K. (2014). A compact Airy beam light sheet microscope with a tilted cylindrical lens. Biomed. Opt. Express.

[11] Shechtman Y., Weiss L.E., Moerner W.E. (2015). Precise three-dimensional scan-free multiple-particle tracking over large axial ranges with tetrapod point spread functions. Nano Lett..

[12] Huang B., Wang W., Zhuang X. (2008). Three-dimensional super-resolution imaging by stochastic optical reconstruction microscopy. Science.

[13] Juette M.F., Gould T.J., Bewersdorf J. (2008). Three-dimensional sub-100 nm resolution fluorescence microscopy of thick samples. Nat. Methods.

[14] Horrocks M.H., Palayret M., Lee S.F. (2014). The changing point-spread function: single-molecule-based super-resolution imaging. Histochem. Cell Biol..

[15] Pavani S.R.P., Thompson M.A., Moerner W.E. (2009). Three-dimensional, single-molecule fluorescence imaging beyond the diffraction limit by using a double-helix point spread function. Proc. Natl. Acad. Sci. USA.

[16] Thompson M.A., Lew M.D., Moerner W.E. (2010). Localizing and tracking single nanoscale emitters in three dimensions with high spatiotemporal resolution using a double-helix point spread function. Nano Lett..

[17] Lew M.D., Lee S.F., Moerner W.E. (2011). Corkscrew point spread function for far-field three-dimensional nanoscale localization of pointlike objects. Opt. Lett..

[18] Shechtman Y., Sahl S.J., Moerner W.E. (2014). Optimal point spread function design for 3D imaging. Phys. Rev. Lett..

[19] Badieirostami M., Lew M.D., Moerner W.E. (2010). Three-dimensional localization precision of the double-helix point spread function versus astigmatism and biplane. Appl. Phys. Lett..

[20] Sharonov A., Hochstrasser R.M. (2006). Wide-field subdiffraction imaging by accumulated binding of diffusing probes. Proc. Natl. Acad. Sci. USA.

[21] Kanchanawong P., Waterman C.M. (2012). Advances in light-based imaging of three-dimensional cellular ultrastructure. Curr. Opin. Cell Biol..

[22] Lew M.D., Lee S.F., Moerner W.E. (2011). Three-dimensional superresolution colocalization of intracellular protein superstructures and the cell surface in live Caulobacter crescentus. Proc. Natl. Acad. Sci. USA.

[23] Gahlmann A., Ptacin J.L., Moerner W.E. (2013). Quantitative multicolor subdiffraction imaging of bacterial protein ultrastructures in three dimensions. Nano Lett..

[24] Li D., Shao L., Betzig E. (2015). Extended-resolution structured illumination imaging of endocytic and cytoskeletal dynamics. Science.

[25] Liu Z., Legant W.R., Tjian R. (2014). 3D imaging of Sox2 enhancer clusters in embryonic stem cells. eLife.

[26] Liu Z., Lavis L.D., Betzig E. (2015). Imaging live-cell dynamics and structure at the single-molecule level. Mol. Cell.

[27] Abrahamsson S., Chen J., Gustafsson M.G.L. (2013). Fast multicolor 3D imaging using aberration-corrected multifocus microscopy. Nat. Methods.

[28] Hajj B., Wisniewski J., Dahan M. (2014). Whole-cell, multicolor superresolution imaging using volumetric multifocus microscopy. Proc. Natl. Acad. Sci. USA.

[29] Ghosh S., Preza C. (2013). Characterization of a three-dimensional double-helix point-spread function for fluorescence microscopy in the presence of spherical aberration. J. Biomed. Opt..

[30] Hell S., Reiner G., Stelzer E.H.K. (1993). Aberrations in confocal fluorescence microscopy induced by mismatches in refractive index. J. Microsc..

[31] Jacobsen H., Hell S.W. (1995). Effect of the specimen refractive index on the imaging of a confocal fluorescence microscope employing high aperture oil immersion lenses. Bioimaging.

[32] Saxton M.J. (1997). Single-particle tracking: the distribution of diffusion coefficients. Biophys. J..

[33] Dunne P.D., Fernandes R.A., Klenerman D. (2009). DySCo: quantitating associations of membrane proteins using two-color single-molecule tracking. Biophys. J..

[34] Ruprecht V., Brameshuber M., Schütz G.J. (2010). Two-color single molecule tracking combined with photobleaching for the detection of rare molecular interactions in fluid biomembranes. Soft Matter.

[35] Kusumi A., Tsunoyama T.A., Fujiwara T.K. (2014). Tracking single molecules at work in living cells. Nat. Chem. Biol..

[36] Chen J., Zhang Z., Liu Z. (2014). Single-molecule dynamics of enhanceosome assembly in embryonic stem cells. Cell.

[37] Greiss F., Deligiannaki M., Braun D. (2016). Single-molecule imaging in living drosophila embryos with reflected light-sheet microscopy. Biophys. J..

[38] Grimm J.B., English B.P., Lavis L.D. (2015). A general method to improve fluorophores for live-cell and single-molecule microscopy. Nat. Methods.

[39] Herbert, A. Single Molecule Light Microscopy ImageJ Plugins. www.sussex.ac.uk/gdsc/intranet/microscopy/imagej/smlm_plugins. Accessed 2014.

[40] Lew M.D., von Diezmann A.R., Moerner W.E. (2013). Easy-DHPSF open-source software for three-dimensional localization of single molecules with precision beyond the optical diffraction limit. Protoc. Exch..

[41] Kazhdan, M., M. Bolitho, and H. Hoppe. 2006. Poisson surface reconstruction. *SGP ’06 Proc. Symp. Geometry Processing, Cagliari, Italy.* 61–70.

[42] Kazhdan M., Hoppe H. (2013). Screened poisson surface reconstruction. ACM Trans. Graph..

[43] Weimann L., Ganzinger K.A., Klenerman D. (2013). A quantitative comparison of single-dye tracking analysis tools using Monte Carlo simulations. PLoS One.

[44] Qian H., Sheetz M.P., Elson E.L. (1991). Single particle tracking. Analysis of diffusion and flow in two-dimensional systems. Biophys. J..

[45] Brown A.C.N., Oddos S., Davis D.M. (2011). Remodelling of cortical actin where lytic granules dock at natural killer cell immune synapses revealed by super-resolution microscopy. PLoS Biol..

[46] Pancer Z., Cooper M.D. (2006). The evolution of adaptive immunity. Annu. Rev. Immunol..

[47] Garboczi D.N., Ghosh P., Wiley D.C. (1996). Structure of the complex between human T-cell receptor, viral peptide and HLA-A2. Nature.

[48] van der Merwe P.A., Dushek O. (2011). Mechanisms for T cell receptor triggering. Nat. Rev. Immunol..

[49] O’Shaughnessy A., Hendrich B. (2013). CHD4 in the DNA-damage response and cell cycle progression: not so NuRDy now. Biochem. Soc. Trans..

[50] Zhang W., Aubert A., Laue E.D. (2016). The nucleosome remodeling and deacetylase complex NuRD is built from preformed catalytically active sub-modules. J. Mol. Biol..

[51] Tokunaga M., Imamoto N., Sakata-Sogawa K. (2008). Highly inclined thin illumination enables clear single-molecule imaging in cells. Nat. Methods.

[52] Huang F., Sirinakis G., Bewersdorf J. (2016). Ultra-high resolution 3D imaging of whole cells. Cell.

[53] Pernis B. (1985). Internalization of lymphocyte membrane components. Immunol. Today.

[54] Legant W.R., Shao L., Betzig E. (2016). High-density three-dimensional localization microscopy across large volumes. Nat. Methods.

[55] Monks C.R.F., Freiberg B.A., Kupfer A. (1998). Three-dimensional segregation of supramolecular activation clusters in T cells. Nature.

[56] Grakoui A. (1999). The immunological synapse: a molecular machine controlling T cell activation. Science.

[57] Finetti F., Onnis A., Baldari C.T. (2015). Regulation of vesicular traffic at the T cell immune synapse: lessons from the primary cilium. Traffic.

[58] Choudhuri K., Llodrá J., Dustin M.L. (2014). Polarized release of T-cell-receptor-enriched microvesicles at the immunological synapse. Nature.

[59] James J.R., White S.S., Klenerman D. (2007). Single-molecule level analysis of the subunit composition of the T cell receptor on live T cells. Proc. Natl. Acad. Sci. USA.

[60] Adler J., Shevchuk A.I., Parmryd I. (2010). Plasma membrane topography and interpretation of single-particle tracks. Nat. Methods.

[61] Jung Y., Riven I., Haran G. (2016). Three-dimensional localization of T-cell receptors in relation to microvilli using a combination of superresolution microscopies. Proc. Natl. Acad. Sci. USA.

[62] Mege J.-L., Capo C., Bongrand P. (1986). Quantification of cell surface roughness; a method for studying cell mechanical and adhesive properties. J. Theor. Biol..

[63] Zhu D.-M., Dustin M.L., Golan D.E. (2007). Analysis of two-dimensional dissociation constant of laterally mobile cell adhesion molecules. Biophys. J..

[64] Jönsson P., Southcombe J.H., Klenerman D. (2016). Remarkably low affinity of CD4/peptide-major histocompatibility complex class II protein interactions. Proc. Natl. Acad. Sci. USA.

[65] Sage P.T., Varghese L.M., Carman C.V. (2012). Antigen recognition is facilitated by invadosome-like protrusions formed by memory/effector T cells. J. Immunol..

[66] Lee S.A., Ponjavic A., Biteen J.S. (2016). Nanoscopic cellular imaging: confinement broadens understanding. ACS Nano.

[67] Ritter J.G., Veith R., Kubitscheck U. (2010). Light sheet microscopy for single molecule tracking in living tissue. PLoS One.

[68] Yu J., Cao B., Niu H. (2014). Improved localization accuracy in double-helix point spread function super-resolution fluorescence microscopy using selective-plane illumination. Proc. SPIE..

[69] Hu Y.S., Cang H., Lillemeier B.F. (2016). Superresolution imaging reveals nanometer- and micrometer-scale spatial distributions of T-cell receptors in lymph nodes. Proc. Natl. Acad. Sci. USA.

[70] Ricci M.A., Manzo C., Cosma M.P. (2015). Chromatin fibers are formed by heterogeneous groups of nucleosomes in vivo. Cell.

[71] El Beheiry M., Dahan M. (2013). ViSP: representing single-particle localizations in three dimensions. Nat. Methods.

