# Supplementary material for: Three-Dimensional Super-Resolution in Eukaryotic Cells Using the Double-Helix Point Spread Function
Source: Biophys J. 2017 Apr 11;112(7):1444–54. doi: 10.1016/j.bpj.2017.02.023 (PMC5390298; doi:10.1016/j.bpj.2017.02.023)
Supplement: Document S1. Supporting Materials and Methods, Figs. S1–S16, and Table S1 [file mmc1.pdf]

Biophysical Journal, Volume *112*

Supplemental Information

Three-Dimensional Super-Resolution in Eukaryotic Cells Using the Double-Helix Point Spread Function

Alexander R. Carr, Aleks Ponjavic, Srinjan Basu, James McColl, Ana Mafalda Santos, Simon Davis, Ernest D. Laue, David Klenerman, and Steven F. Lee

Supplementary Information

1. Imaging above the coverslip with water and oil immersion objective lenses

Using a water immersion objective lens reduces aberration of the DHPSF when imaging above the coverslip if $n_{media} \approx n_{water} \approx 1.33$. DHPSFs from different depths above the coverslip and their rotation are shown in Figure S1. 100 µL of a ~$3.6 \cdot 10^8$ particles/mL solution of fluorescent beads (TetraSpeck Microspheres, 0.1 µm, ThermoFisher) in 1% agarose solution ($n \approx n_{water} \approx 1.33$) was imaged across a depth of 50 µm. Significant deterioration of the DHPSF is seen when imaging with an oil immersion objective lens (Plan Apo TIRF 60×, Nikon), whereas no significant deterioration is seen when imaging with a water immersion objective lens (Plan Apo VC 60× H, Nikon).

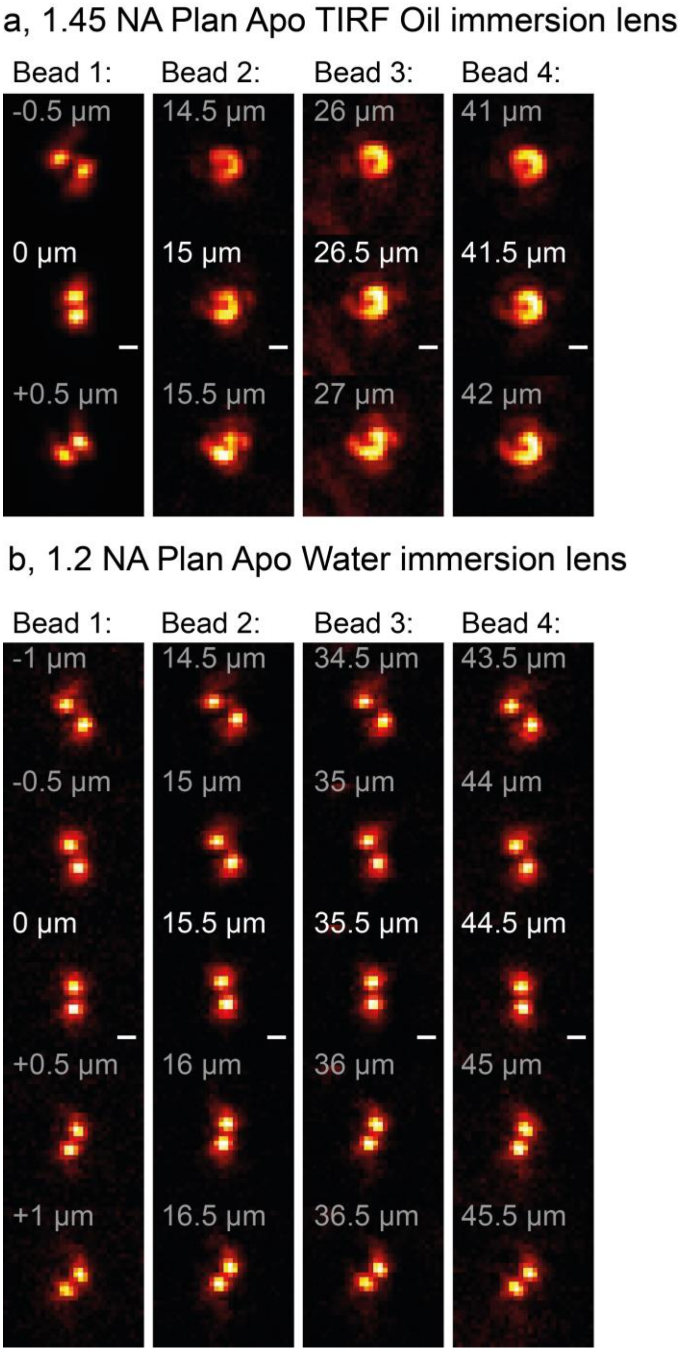

SI Figure S1 Imaging fluorescent beads with suspended in 1% agarose with the DHPSF above the coverslip. Labels represent the distance above the coverslip for each image plane. Scale bars are 1 µm **(a)** Images of 4 different fluorescent beads captured with an oil immersion objective at different distances above the coverslip. Significant deformation of the DHPSF can be seen away from the coverslip surface. **(b)** Images of 4 different fluorescent beads captured with a water immersion objective. No significant deformation of the DHPSF can be observed even up to 50 µm above the surface.

2. DHPSF aberration as a function of axial position of focal plane

The effect of the axial position of the focal plane on systematic localisation errors induced by aberration was investigated by recording DHPSF calibration stacks at three different axial positions of the focal plane. Three calibration stacks were recorded, at roughly the same area of the field of view, at the coverslip surface, 15 µm above and 30 µm above. The maximum axial depth of the focal plane in all presented experiments was ~15 µm.

Tetraspeck fluorescent beads were suspended in 1% Pytagel and imaged at 30ms exposure. Each bead was aligned to be in the focal plane by eye before scanning the objective in 50 nm steps through a distance of 3.5 µm (starting 1.75 µm below the initial plane and moving up). 10 images were recorded at each step. Easy DHPSF was then used to create calibration curves for each bead. There was no obvious difference between curves recorded above the coverslip surface and those at at 15 µm or 30 µm, with all curves overlapping (Figure S2a).

The total 3D Cartesian error between calibration scans was investigated using easy DHPSF to localise the position of the fluorescent beads in these calibration scans. One of the three beads located on the surface was used the calibration stack in easy DHPSF in order to calibrate thresholds for fitting the remaining calibration stacks.

The total Cartesian error between calibration stacks was determined at each 50 nm axial step for each bead using the mean position of localisations from the respective image plane in x, y and z. This was evaluated with the equation: $r = \sqrt{r_x^2 + r_y^2 + r_z^2}$, where r is the total Cartesian error and $r_{x,y,z}$ is the mean displacement in x, y and z dimensions respectively. The x, y and z position of the central axial step for each bead was considered as the origin in order to calculate displacement (the 50 nm z steps expected between successive axial planes were subtracted from the z displacement).

Significant localisation errors increasing towards the periphery of the depth of field were observed reaching up to ~100 nm at the extremes, however, no difference between calibration curves recorded at different heights above the coverslip were seen. This indicates that the cause of this error source is not aberration induced by imaging away from the cover slip. These errors are small compared to the distances considered in quantitative analysis and SI section 9 ‘Benchmarking of 3D MSD analysis’ demonstrates that diffusion measurements are robust even at low localisation precision (corresponding to low photon number in the figure) for the values presented.

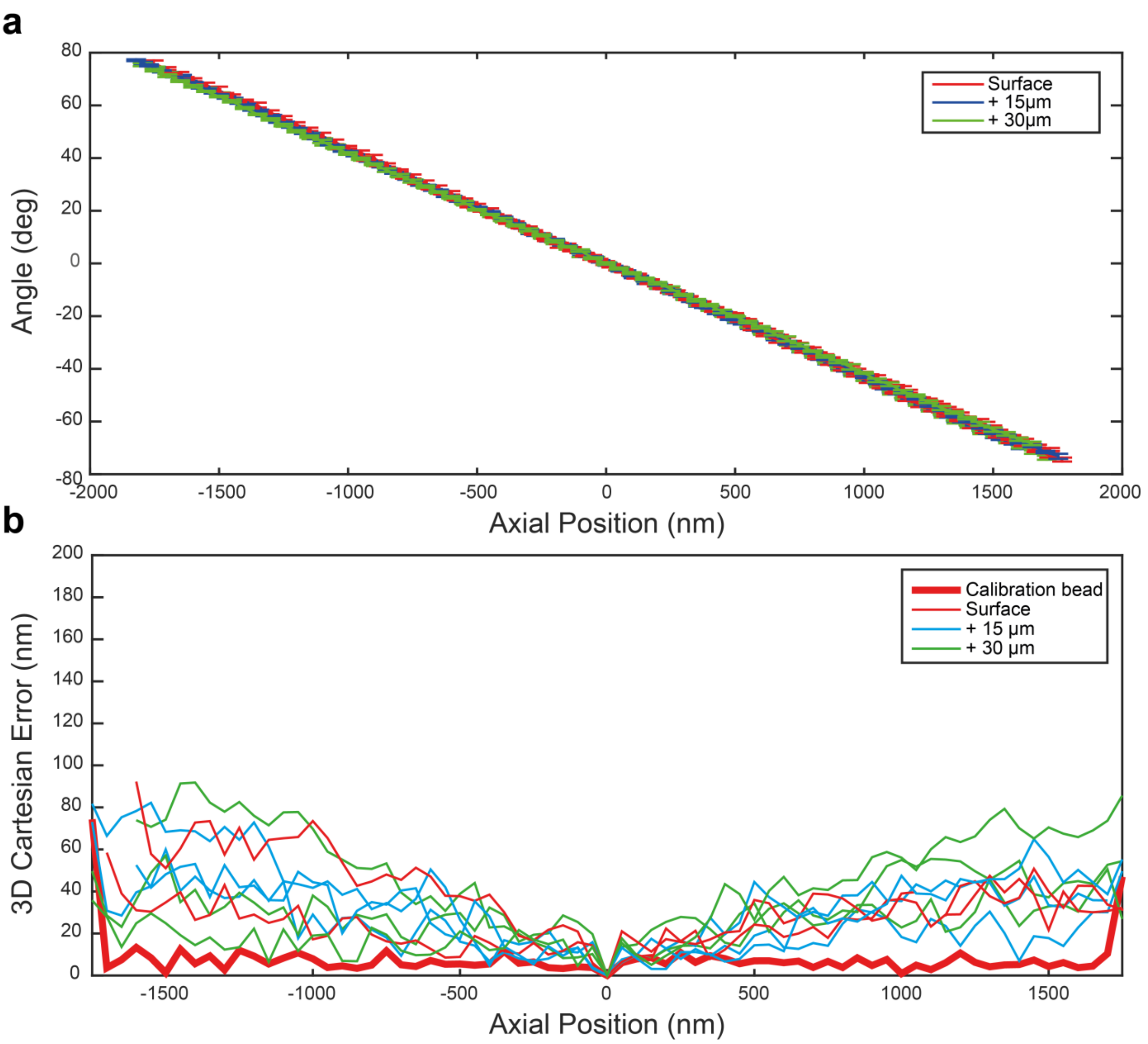

Figure S2 The effect of focal plane axial depth on 3D Cartesian error. Tetraspeck fluorescent beads were imaged at different depths above the coverslip surface in 50 nm axial steps. **(a)** Calibration curves of angle between DHPSF lobes as a function of axial depth for three different fluorescent beads at each axial depth; at the surface (red), 15 μm above (blue) and 30 μm above (green). The majority of the curves cannot be seen due to overlap. **(b)** Total Cartesian error as a function of axial depth for three different fluorescent beads at each axial depth; at the surface (red), 15 μm above (blue) and 30 μm above (green). The bead used for calibration in the easy DHPSF reconstruction is highlighted (thick red line).

3. DHPSF microscope schematic

A bespoke DHPSF microscope was built as described in the main text and as shown in Figure S3.

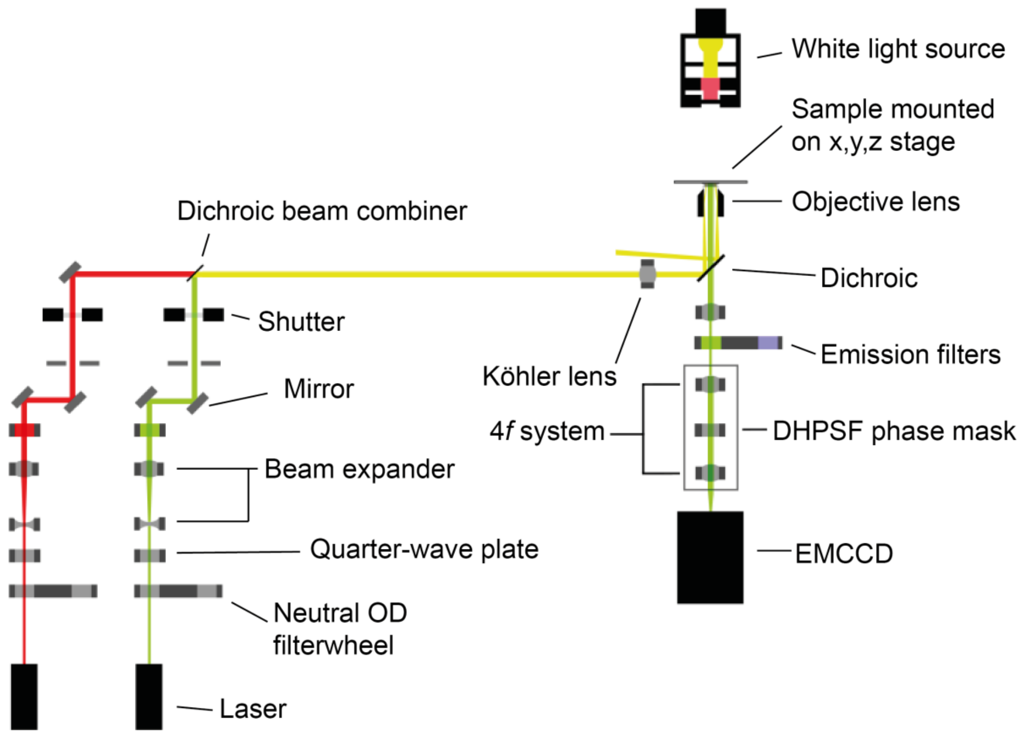

Figure S3 DHPSF microscope schematic. A combined beam path of excitation (561 nm) and activation (405 nm) lasers is shown.

4. Typical detected photon ranges for experimental data

The number of photons detected was given by the sum of the integrated signal from a Gaussian fit to each DHPSF lobe. The signal was given by the total ADU signal from the camera divided by the total camera gain (~38 ADU/photon in the presented experiments).

A histogram was created containing the number of detected photons in each localisation for each different fluorophore and exposure length (Figure S4), which is summarised in SI table 1. Error limits for the corresponding localisation precision were estimated from the distribution of points in Figure 1a.

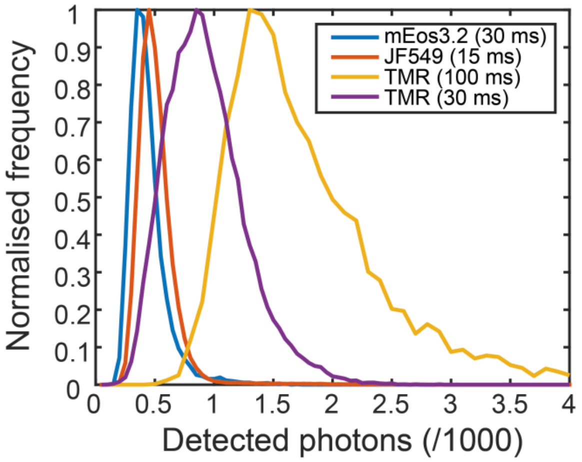

Figure S4 Number of photons detected. Histograms of the number of photons detected during typical imaging in presented data. mEos3.2 (30 ms) and TMR (100ms) were used in localisation imaging whereas JF549 (15 ms) and TMR (30ms) were used in SPT.

Fluorophore	Exposure (ms)	Peak Detected Photons	5th percentile	95th percentile	Back-ground photons per pixel	Peak x,y Localisation precision (nm)	Peak z localisation precision (nm)
JF549	15	450	300	700	6.7 ± 0.5	24 ± 2	55 ± 5
mEos3.2	30	350	250	700	5.4 ± 0.1	26 ± 2	59 ± 5
TMR	30	850	450	1550	10.2 ± 0.4	19 ± 2	43 ± 5
TMR	100	1350	950	3000	24.3 ± 0.4	15 ± 2	33 ± 5

Table S1 Summary of detected photon values from SI Figure 4 with number of background photons per pixel per frame from within cell volumes and lateral and axial localisation precisions.

5. Optimal overlapping of imaging planes for flat localisation density

In order to achieve the most flat localisation density across volumes spanning multiple imaging planes the optimal offset between planes was experimentally determined to be ~3 μm or ~75% of the working depth of field.

First, the localisation density of a single plane was measured by imaging the membrane protein TCR via HaloTag-TMR in the central plane of 5 T cells. Localisations were sorted into 500 nm bins axially and their relative density calculated (Figure S5a). The localisation density correlated well to the axial intensity distribution of the DHPSF as output from easy-DHPSF (Figure S5b), which is expected as the ability to localise PSFs is directly related to the detected photons.

An approximate distribution removing noise in the centre of the density distribution (shown in Figure S5a) was used as example image planes to determine the optimal plane offset. Two identical approximate distributions were plotted with one axially offset by between 2 μm and 5 μm in 500 nm steps. For each offset, the mean localisation density was calculated from a 4 μm thick section including the interface between imaging planes from the sum of the two distributions:

$$\langle density_{total} \rangle = \langle density_1 + density_2 \rangle$$

An overlap of 3-3.5 μm was found to yield the most-flat localisation density across the interface between two imaging planes (highlighted area in Figure S5c).

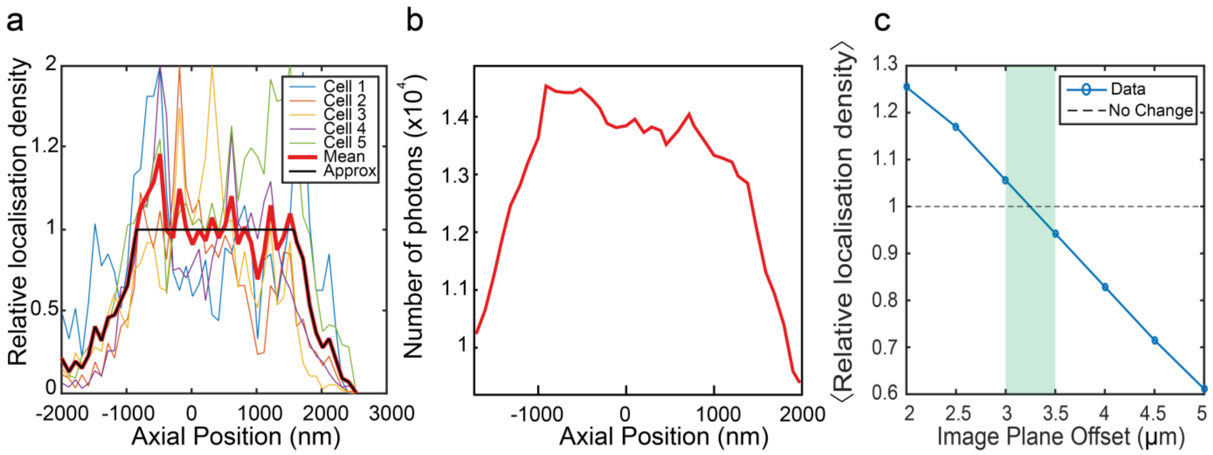

Figure S5 Optimal overlapping of successive image planes. **(a)** Localisation density across a single image plane from 5 cells with average (thick red) and approximate (black) distributions. **(b)** Number of photons detected from a single PSF across the depth of field during DHPSF calibration. **(c)** Relative localisation density across a 4 μm thick section at the interface of two successive simulated imaging planes. The highlighted green area represents the optimal range of plane offset to achieve the most flat localisation density across large samples.

6. Focal drift

The drift of the system was measured by imaging Tetraspeck beads suspended in 1% Phytagel (P8169, Sigma). Beads were imaged in the centre of the field of view for 12,000 frames at 100ms exposure. The mean position of the beads in three dimensions from successive sections of 100 frames was used to track drift (Figure S6). This was repeated 7 times. A linear fit was used to estimate the expected drift as a function of time. These measurements represent the typical drift of the imaging system used to conduct the experiments presented in the manuscript.

The drift was found to follow the equation: $\Delta r = (0.0044 \pm 0.0003)N + (1.3 \pm 1.8)$. Where Δr is the Cartesian drift distance and N is the number of frames (at 100ms exposure). Using this fit, the 20,000 frame acquisitions presented in the manuscript would expect total drift of 89 ± 8 nm by the end of the experiment. However, the majority of the localisations will be recorded with less drift as the density of fluorescence events decays over the course of the experiment.

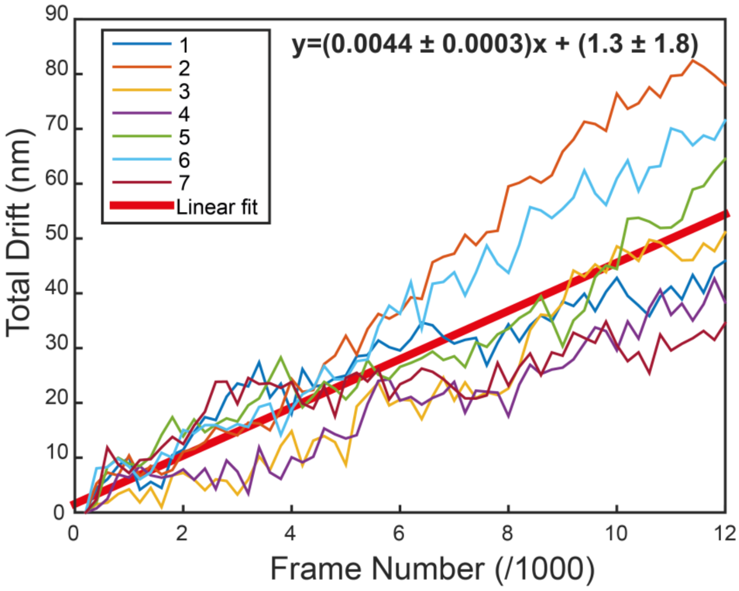

Figure S6 Drift of microscope platform over time. The Cartesian distance drifted as a function of frame number (at 100ms exposure) is plotted for 7 repeats. A linear fit (thick red line) was used to estimate the expected drift at different time points.

7. Lateral offset as a function of axial distance into sample

The lateral position of successive imaging planes relative to each other was examined by measuring the displacement of Tetraspeck beads suspended in 1% Phytagel (P8169, Sigma). Beads were imaged at two image planes, first near the coverslip and then 2.5 μm above this so that the same bead was still in the depth of field. 100 frames were acquired at each image plane before moving to the next. This was repeated 60 times, as in the experiments presented in Figures 2 and 3, acquiring a total of 12,000 frames for each bead.

The average position of each bead was compared between successive image planes for each repeat using with the Cartesian distance between the two centres plotted on a histogram (Figure S7). This histogram was fit with a 1-dimensional Gaussian to extract the centre position and width.

It was determined that moving an axial distance of +2.5 μm resulted in a 9.1 ± 4.4 nm lateral shift. This results in a lateral shift of ~45.5 nm across the presented imaging volumes (~12.5 μm). This is comparable to the localisation precision of the DHPSF and is small compared to the accuracy of the fitted mesh and to the quantitative values considered in the whole-cell scanning experiments, which look at the distance between protein molecules across the whole ~12.5 μm thick volume (see Figure S13, the statistical fluctuations of the presented distances are on the order of ~1-2 μm).

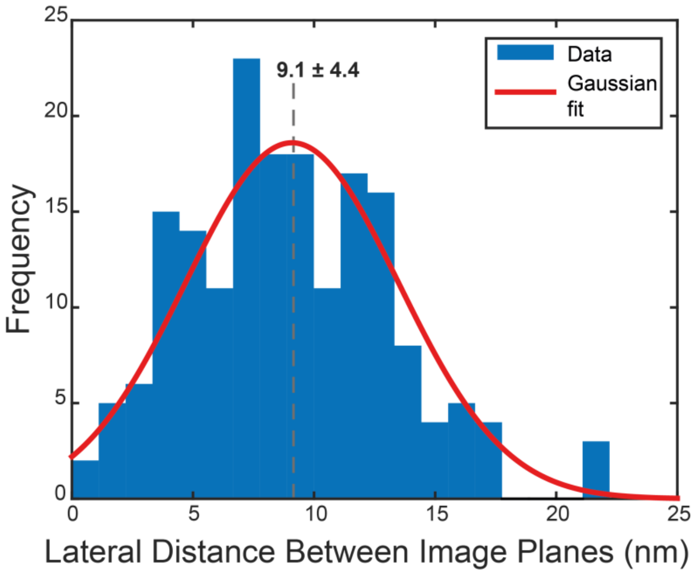

Figure S7 Lateral offset between successive image planes. A histogram of the measured Cartesian lateral offset between successive image planes separated by 2.5 μm axially. A1D Gaussian function was fit to the data (red line) in order to extract the centre position and width, which are labelled.

8. Mesh fitting to localisation data

Using a standard method and functions included in Meshlab (http://meshlab.sourceforge.net) 3D localisation data was converted into an object mesh via the following steps:

1. Import localisation data as point could into meshlab from '.xyz' file type
2. Create normals to each localisation
   a. The built in 'Compute Normals for Point Set' function in the 'Normals, Curvatures and Orientation' tab of the 'Filters' menu was used. An input number of neighbours ranging between 50 and 200 depending on localisation density.
3. Reconstruct a surface mesh using the Poisson surface approach
   a. The built in 'Surface Reconstruction: Poisson' function in the 'Remeshing, Simplification and Reconstruction' tab of the 'Filters' menu was used. Typically an 'Octree Depth' of 10, a 'Solver Divide' of 6, a 'Samples per Node' of 1 and a 'Surface Offsetting' of 1 were selected but these parameters were adjusted slightly between datasets for best results.
4. Uniformly sample the mesh
   a. The built in 'Uniform Mesh Resampling' function in the 'Remeshing, Simplification and Reconstruction' tab of the 'Filters' menu was used

Figure S8 shows a fitted mesh to the localisation data presented in Figure 2 in the main text. Both the mesh and the individual points are shown with an inset zooming in to a 1.5 μm thick section showing how the mesh follows the approximate distribution of the localisations. The localisation and mesh datasets are provided as additional supplementary files.

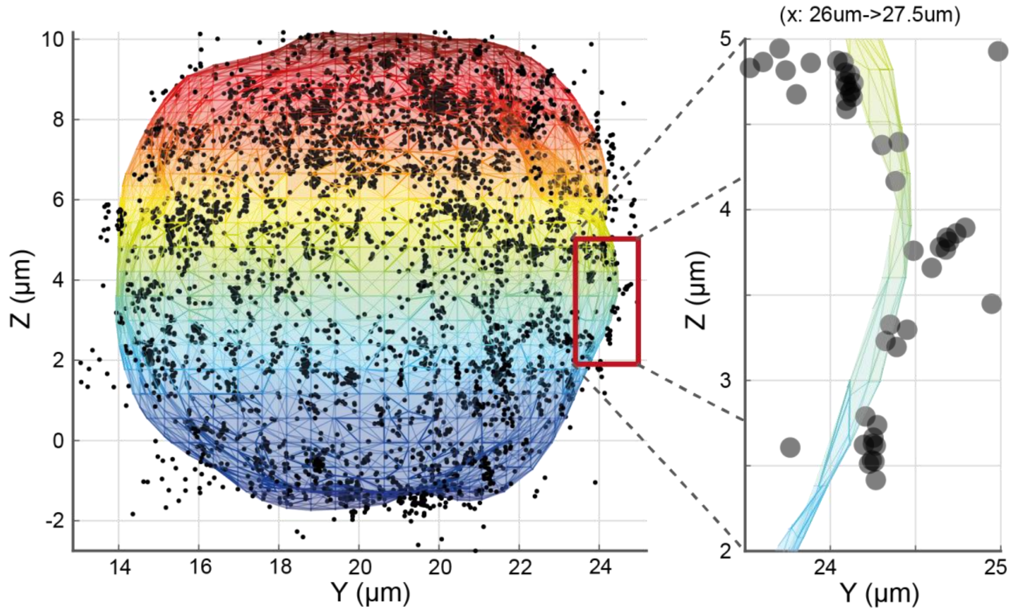

Figure S8 Fitting a mesh to localisation data. Individual localisations from Figure 2 are represented as dark grey dots. The fitted mesh is coloured from red to blue from top to bottom for visibility. The inset shows a zoom in on a 1.5 μm thick volume.

9. Benchmarking of 3D MSD analysis

In order to verify the MSD written analysis algorithm its performance was tested on 2D and 3D data sets. When analysing 2D data each localisation was assigned an axial position of 0 nm in all cases to satisfy the data format required for the algorithm.

A 2D TIRF dataset of TCR diffusing on the basal surface of 12 T cells on passivating coated glass surfaces was used to benchmark the written algorithm's performance against a previously published 2D MSD analysis code (Weimann et al (1)). Unlike the written algorithm, the previously published code includes a 2D fitting step. Peakfit (2) was used to create a localisation list that was then used as the input for the written algorithm. The overall MSD curves produced from all trajectories and calculated diffusion coefficients are shown in Figure S9a. All points on the MSD curves are within error between the two MSD analysis codes and the calculated diffusion coefficients agree well.

The written algorithm was also benchmarked on simulated 3D datasets across a range of diffusion coefficients. Simulated datasets were created by distributing points randomly in space and moving each point in 3 dimensions by a distance randomly sampled from a normal distribution with a width equal to the one dimensional mean squared displacement for each frame.

$$MSD = \sqrt{2 * D * dt}$$

Where D is the diffusion coefficient being simulated and dt is the simulated time between localisations.

For each frame, an additional movement was added in all the dimensions to simulate the localisation precision of the instrument. This distance was sampled from a normal distribution with width equal to the measured lateral localisation precision in x and y and the axial localisation precision in z at a given intensity (distributions can be seen in Figure 1 in main text).

All simulated trajectories were 16 frames long as this is the minimum number of points a trajectory requires to be considered in the analysis code. This is in order to be able to create 4 msd points for a linear fit as the number of MSD points should not exceed 25% of the total number of points in a trajectory, as stated by Saxton (3).

The recall of the simulated 3D diffusion coefficients was determined from 20 simulated data sets at each simulated diffusion coefficient as defined by:

$$Recall = {}^{D_{calculated}}/_{D_{simulated}}$$

Firstly, the effect of the number of tracks considered on recall was investigated using a typical experimental localisation precision of 25 nm laterally and 50 nm axially and a time step of 30 ms (Figure S9b-c). The mean error in recall was below 2% after 100 trajectories are considered and the standard deviation of recall was below 5% after 200 trajectories are considered for all simulated diffusion coefficients. This indicates that after 200 trajectories of minimal length the analysis is robust to diffusion coefficients of 0.01 $\mu m^2/s$, with less trajectories required for faster diffusion coefficients.

The effect of localisation precision as determined by the number of detected photons on recall was investigated (Figure S9d-e). 400 tracks were considered with a time step of 30 ms from 20 simulated datasets for each simulated diffusion coefficient. The mean error in recall was below 2% after 100 photons, which is below the sensitivity of the microscope, for all simulated diffusion coefficients. The standard deviation of recall was below 5% for simulated diffusion coefficients of 0.1 $\mu m^2/s$ and higher

at all considered detected photon numbers. For a simulated diffusion coefficient of 0.01 µm^2/s, ~500 detected photons results in a standard deviation of recall <5%. This indicates that, at all experimentally possible detected photons numbers, analysing 400 trajectories of minimal length the analysis is robust at diffusion coefficients >=0.1 µm^2/s. To analyse slower diffusion coefficients a mean detected photon number of ~>500 is required. These numbers all fit with experimental conditions that were used in all data presented in the main text.

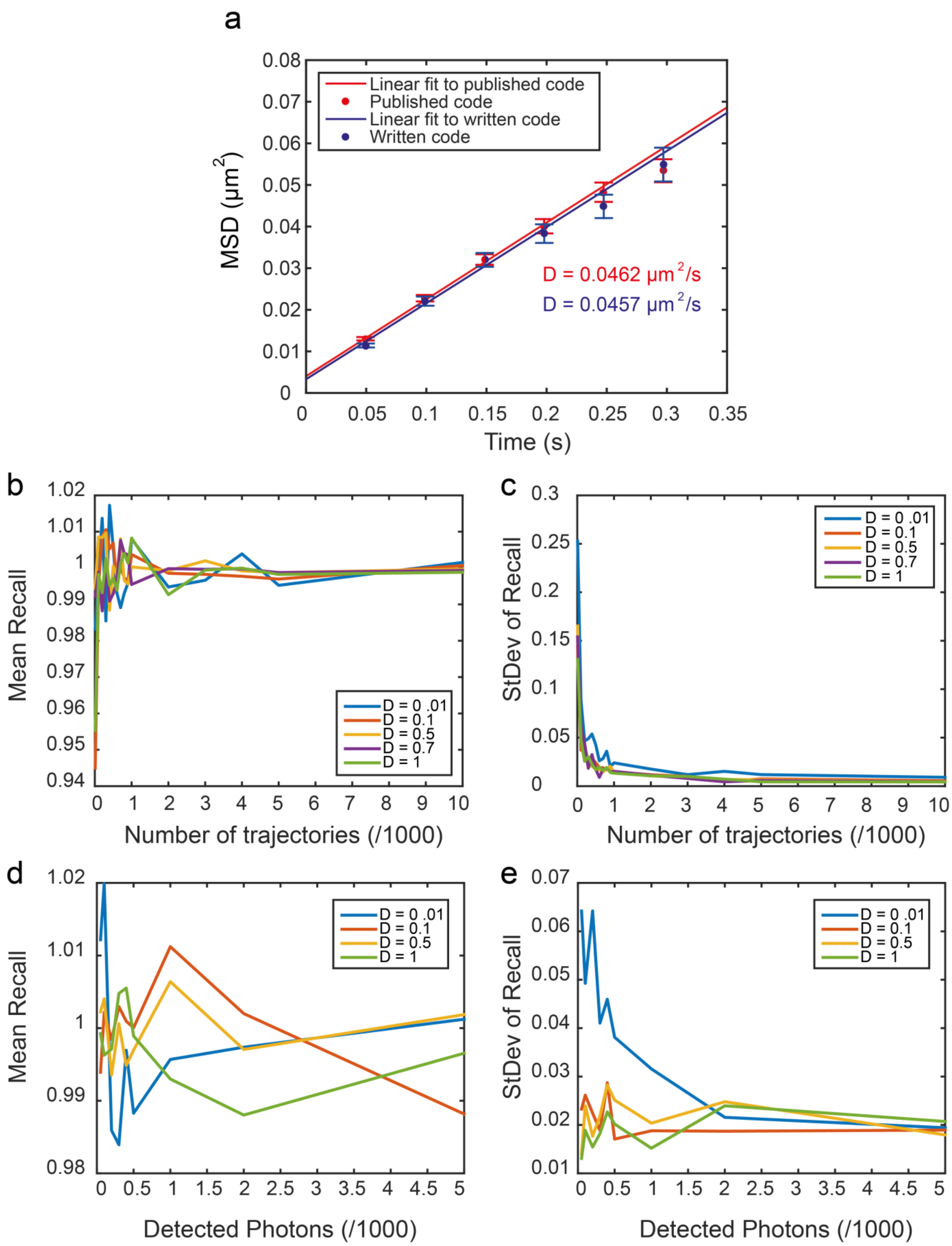

Figure S9 Benchmarking of written MSD analysis algorithm. **(a)** Comparing analysis of a 2D data set to a previously published MSD analysis algorithm. **(b)** Mean recall in diffusion coefficient from 20 simulated datasets at a range of diffusion coefficients as a function of the number of trajectories considered. **(c)** Standard deviation in recall of simulated datasets from (b). **(d)** Mean recall in diffusion coefficient from 20 simulated datasets at a range of diffusion coefficients as a function of detected photons. **(e)** Standard deviation in recall of simulated datasets from (d).

10. Identifying bound trajectories by R^2 analysis of MSD linear fit

In order to separate bound and unbound trajectories during MSD analysis individual MSD curves were compared to a linear fit to the first 4 points. Trajectories whose MSD curve did not fit well to a linear fit, by measure of R^2 value, were considered to be bound. Bound and unbound trajectories could then be analysed separately. In order to determine the threshold R^2 value to best separate the populations simulated data was analysed. As described above, 600 trajectories were simulated using diffusion coefficients of 0 μm^2/s (bound), 0.1 μm^2/s (unbound, typical membrane protein) and 0.7 μm^2/s (unbound, typical of CHD4), each with axial and lateral localisation precisions centred at expected values (see Table S1). The first 4 points of the MSD curve of each trajectory was fit to a straight line and the R^2 value recorded. A cumulative histogram for the R^2 values of each population was created (Figure S10). An R^2 value of 0.85 was determined to result in the most accurate identification of diffusion state, identifying >90% of unbound and bound trajectories correctly.

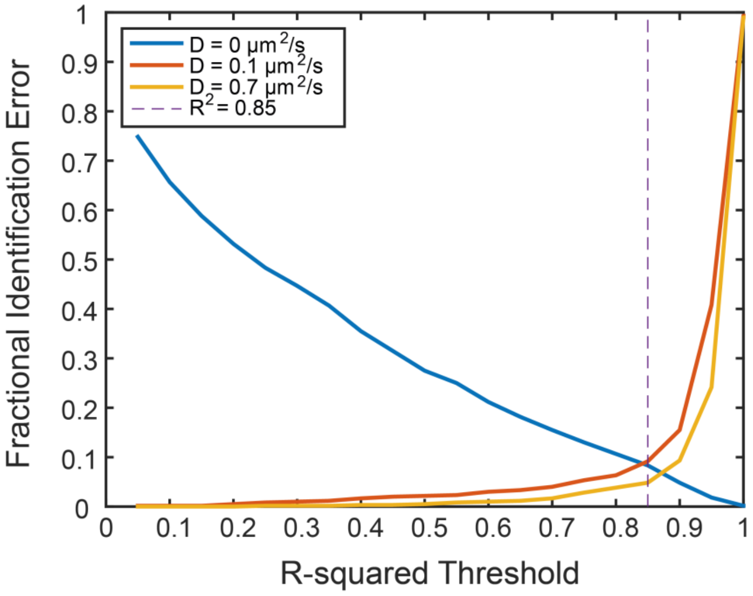

Figure S10 Identifying bound vs unbound populations by R^2 value of linear fit. The fraction of simulated tracks from three diffusion populations that were misidentified as a function of the threshold value chosen. The dashed line is plotted at R^2 = 0.85.

11. Whole cell image data

No significant systematic differences were observed between imaged T cells as shown in SI Figure 11. SI Figure 11 shows additional whole-cell reconstructions of fixed Jurkat T cells expressing CD28-mEos3.2 and TCR-HaloTag-TMR. Localisations are rendered with 200nm localisation precision for visibility.

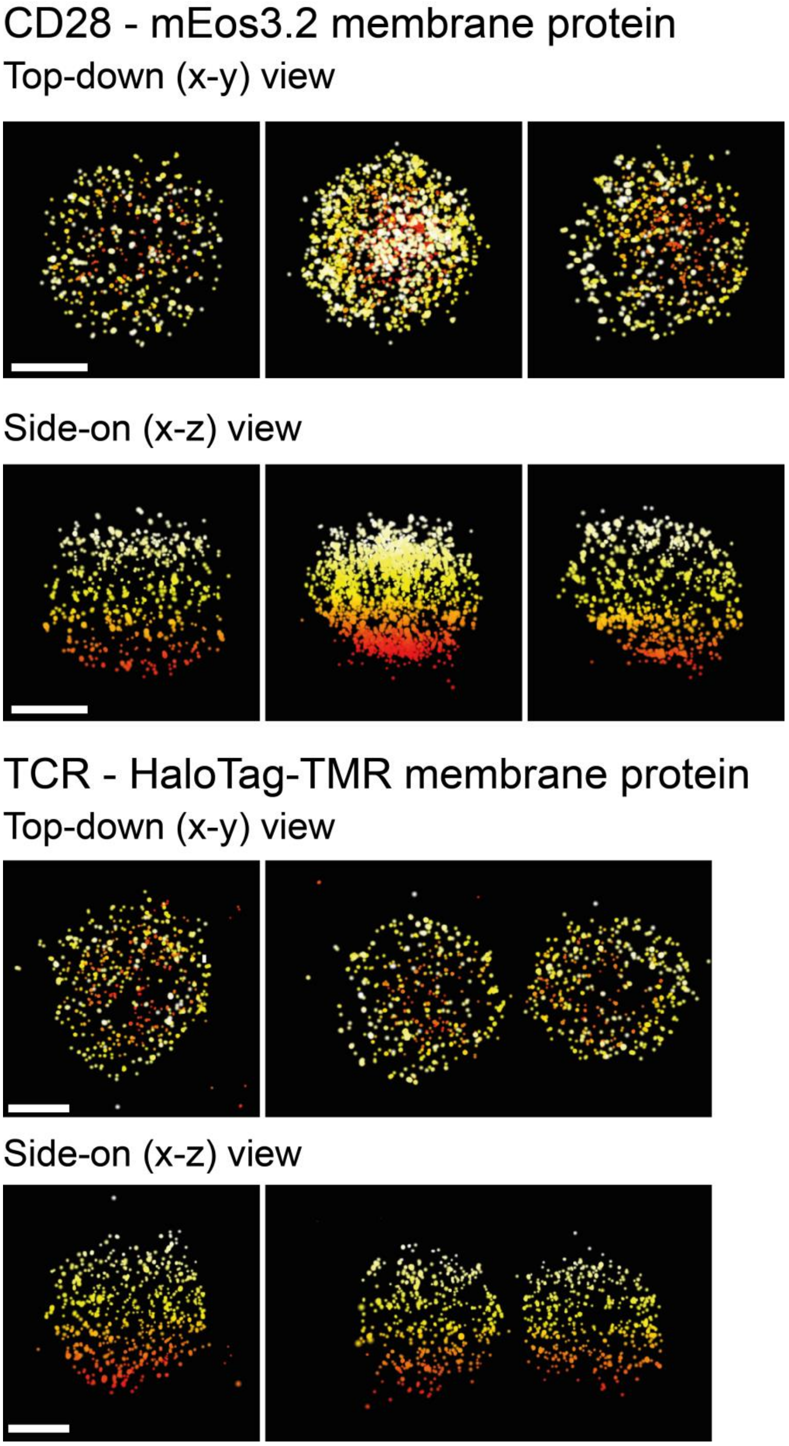

Figure S11 Additional reconstructions of whole-cell super-resolution localisation data of membrane proteins. Side-on and top-down views are shown of three cells expressing CD28-mEos3.2 and three cells expressing TCR-HaloTag-TMR. Localisations are rendered with 200nm precision and scale bars are 5 μm.

12. Nearest neighbour distance of CD28 localisation data

After filtering the localisation data presented in Figure 2a for repeat localisations of the same fluorophore (a radius of 1200 nm and an interval of 3s), the nearest neighbour distance between CD28 molecules was calculated and plotted in a cumulative histogram (Figure S12). 27% of CD28 molecules were revealed to be less than 250 nm apart and 63% were less than 500 nm apart, highlighting the necessity for super-resolution techniques compared to confocal microscopy or other diffraction limited techniques.

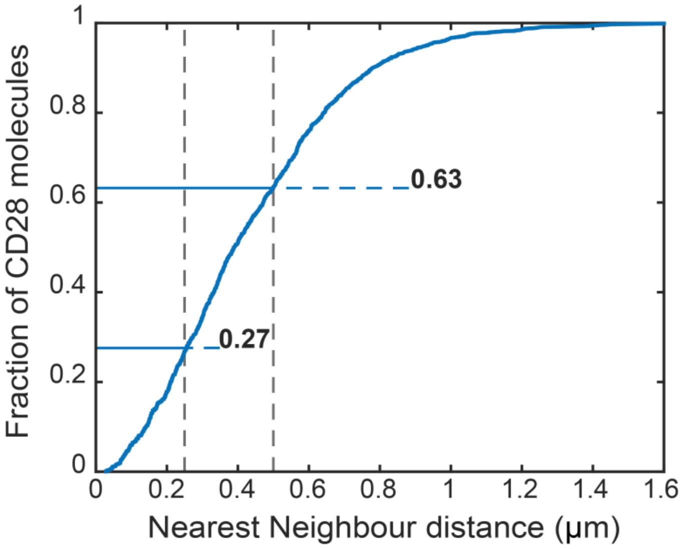

Figure S12 Cumulative histogram of the nearest neighbour distance between CD28 molecules. ~3,000 CD28 molecules were recorded. The two dashed lines indicate a distance of 250 nm and 500 nm. The corresponding fractions at these two distances have been labelled.

13. Correcting for shape with cell-specific models

The distribution of inter-protein distance is highly dependent on the shape of the cell itself. Mostly round cells have an almost uniform distribution due to their symmetry (Figure S13b-c), while other morphologies result in a non-trivial distribution (Figure S13d). In order to correct for cell shape, a model distribution of inter-protein distances was created by randomly sampling fitted meshes. A number of vertices equal to the number of localisations were randomly sampled from vertices on the mesh, 1000 times. Each time the points were randomly translated by 0-250 nm in all three dimensions in order to determine error limits. The difference between the inter-protein distance analysis of the localisation data and model data allows for the effects of shape to be ignored (Figure S13e-g), providing insight into other biological changes.

In all three cells, no significant axial dependence of inter-protein distance distribution was seen (Figure S13e-g).

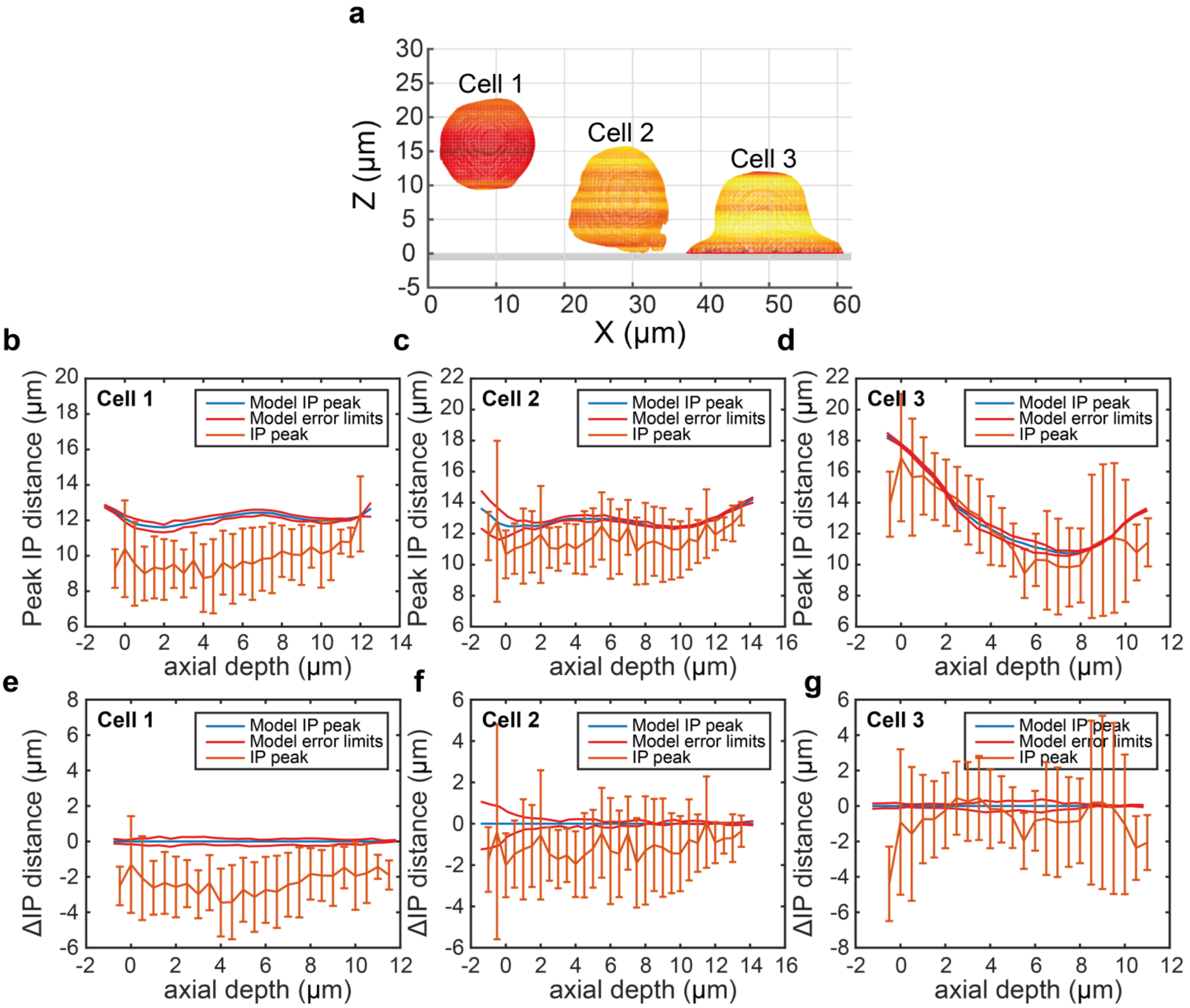

Figure S13 Correcting for the effect of shape on inter-protein (IP) distance distribution. **(a)** Side on cell meshes presented in Figure 3 of the main text. **(b-d)** Peak IP distance of localisation data (orange) and model data (blue) as a function of axial depth from the 3 cells presented in (a). **(e-g)** Shape corrected peak IP distance of localisation data (orange), including error limits determined from model data (red).

14. MSD plot for T cell diffusion data

The ensemble MSD plots for TCR and Zap70 diffusion provide information about the manner of diffusion. The TCR MSD plot fits well to a linear fit indicating free diffusion, while the Zap70 MSD plot drops below the linear fit after the first 4 points indicating a degree of confinement. This is to be expected as TCR is bound to the membrane which forms a closed surface with no edges or obstacles. Zap70 is present intracellularly and thus meets with obstacles such as the outer membrane, nuclear envelope and organelles, partially confining its motion.

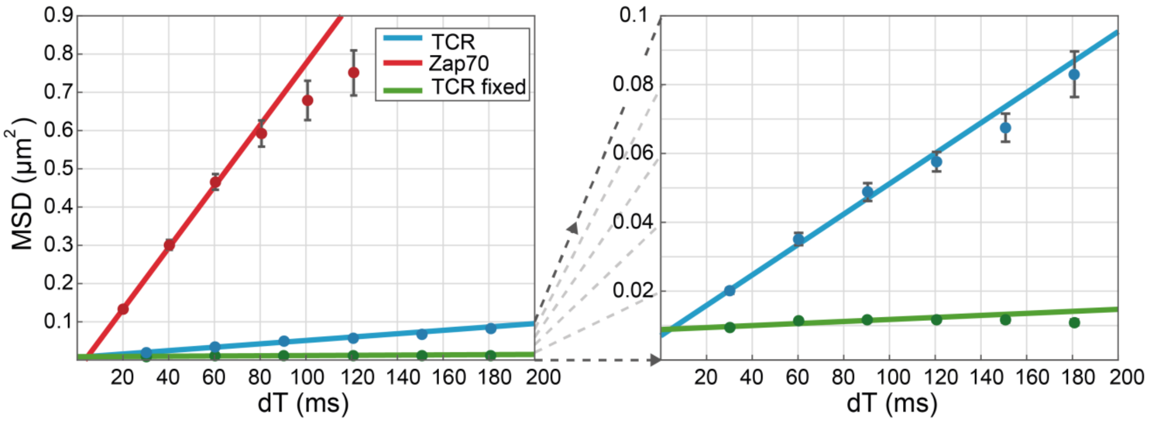

Figure S14 MSD curves for T cell protein diffusion data. (left) MSD plots for Zap70 and TCR live and fixed with linear fits to the first 4 points. (right) Magnification highlighting live and fixed TCR MSD curves.

15. Membrane-bound and cytoplasmic fractions of localised TCR molecules

The smallest distance from each localised protein molecule to the corresponding fitted mesh was used to quantify the fraction of molecules localised in the cytoplasm and on the outer membrane. Duplicate localisations originating from repeat localisation of the same fluorophore were removed by filtering for nearby recorded events in space (<500 nm) and time (<1 second) (results were robust across a large range of thresholding parameters in time and space). The distance between each point and the closest vertex was used to create cumulative histograms for each of the three cells presented (Figure S15).

As the fitted mesh is a weighted approximation of the position of the outer cell membrane, TCR molecules localised on the membrane will not necessarily be found at the fitted mesh. The fraction of molecules differs significantly between the resting cell (0 minutes) and activated cells (5 and 10 minutes) above ~600 nm distances from the fitted meshes.

Analysis of the CD28 localisation data presented in Figure 2 and the corresponding mesh presented in SI Figure 8 was used to determine a threshold separating molecules associated with the outer membrane and intercellular molecules. A significant fraction of CD28 molecules are not associated with the cell's outer membrane but are instead found intracellularly due to protein degradation/creation and the presence of intracellular stores. By analysing only CD28 localisations outside of the mesh volume it is possible to consider a membrane-bound population.

The distance from each CD28 localisation located outside the mesh volume to the nearest vertex was used to create a cumulative histogram. Molecules localised further than 1 μm from the mesh can be considered as located away from the outer membrane of the cell with ~95% certainty (Figure S15a).

For the resting cell, 65% of localised TCR molecules were determined to be on the membrane. For the activated cells (5 and 10 minutes), 72% and 74% of localised TCR molecules were determined to be on the mesh respectively (Figure S15b).

For the resting cell, 30% of TCR molecules were localised intracellularly. For the activated cells (5 and 10 minutes), 17% and 13% of TCR molecules were localised intracellularly respectively (Figure S15c).

For the resting cell, 4% of TCR molecules were localised extracellularly. For the activated cells (5 and 10 minutes), 9% and 12% of TCR molecules were localised extracellularly respectively (Figure S15d. These TCR molecules are more likely associated with long pseudopodia known to be present on the outer membrane of human T cells (4).

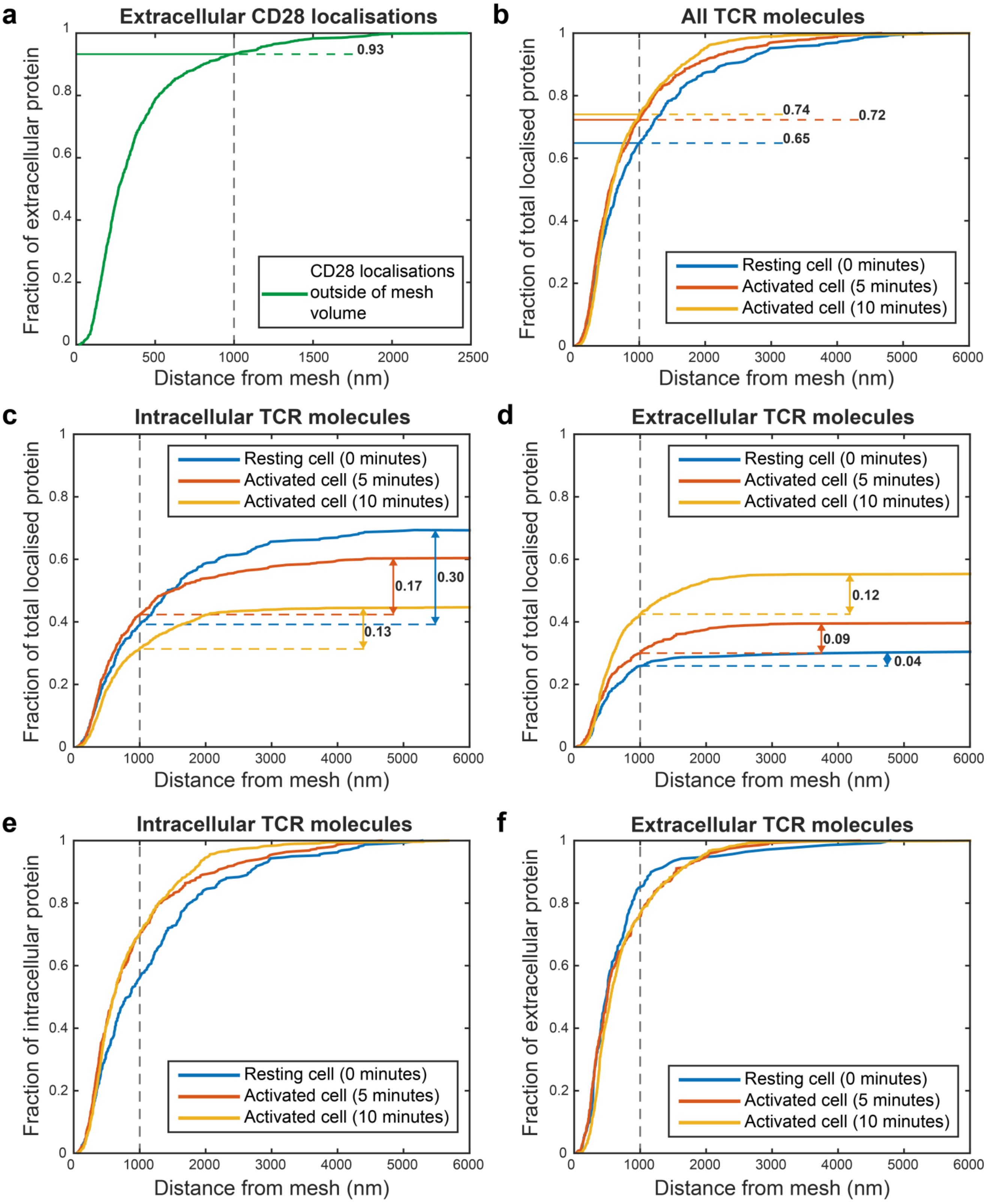

Figure S15 Cumulative histograms of distance from fitted mesh for the cells presented in Figure 2 and 3 in the main text. The grey dashed lines at 1000 nm indicate the threshold used to estimate membrane-bound localisations of TCR molecules. The distribution of the TCR in the resting cell differs significantly from the two activated cells around this threshold. **(a)** Cumulative histogram of the distance from the mesh of CD 28 localisations outside the corresponding mesh volume with the fraction at 1000 nm labelled. **(b)** Cumulative histogram of the distance from the mesh of all TCR molecules to the corresponding mesh with fractions at 1000 nm labelled. **(c)** Cumulative histogram of the distance from the mesh including only TCR molecules localised inside the mesh as a fraction of total number of TCR molecules. The fraction of total localisations further than 1000 nm inside the mesh is labelled for each cell. **(d)** Cumulative histogram of the distance from the mesh including only TCR molecules localised outside the mesh as a fraction of total number of TCR molecules. The fraction of total localisations further than 1000 nm outside the mesh is labelled for each cell. **(e & f)** Cumulative histograms of the distance from the mesh of intracellular and extracellular TCR molecules as a fraction of the number of intracellular and extracellular TCR molecules respectively.

16. 3D diffusion in comparison to 2D models

As described in SI 6, trajectories were simulated diffusing on the top surface of a sphere with a diffusion coefficient of 0.1 $\mu m^2/s$ (Figure S16a) (50 trajectories per sphere, 5 spheres, 20 localisations per track). The trajectories were analysed in 3D using MSD to measure the apparent diffusion coefficient. A 2D projection of the same trajectories was analysed by setting the z-position of each localisation to zero.

The 3D MSD analysis reported a mean diffusion coefficient of 0.103 ± 0.003 $\mu m^2/s$ while the 2D MSD analysis reported a mean diffusion coefficient of 0.078 ± 0.003 $\mu m^2/s$ (Figure S16b). This gives a ratio of 3D to 2D diffusion coefficients of 1.34 ± 0.02, which represents a systematic error in analysing diffusion on a 3D sphere in 2D.

The 3D DHPSF tracking data of TCR presented in Figure 4 was analysed in 2D by setting the z-position of all localisations to zero. The 3D MSD analysis reported a mean diffusion coefficient of 0.110 ± 0.007 $\mu m^2/s$ and the 2D MSD analysis reported a mean diffusion coefficient of 0.064 ± 0.004 $\mu m^2/s$ (Figure S16d). This results in a ratio of 3D to 2D diffusion coefficient of 1.72 ± 0.22, which is considerably larger than expected from diffusion on a sphere. This increase originates from movement away from a spherical surface which can be seen in SI Figure 16c and is likely caused by ruffles and pseudopodia in the T cell outer membrane.

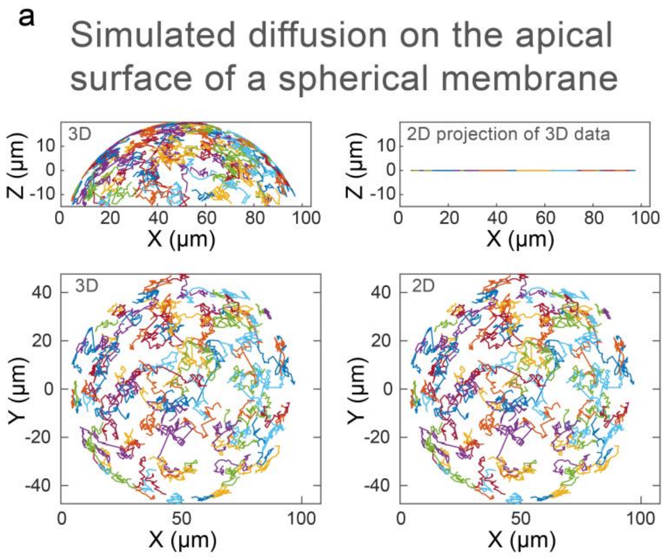

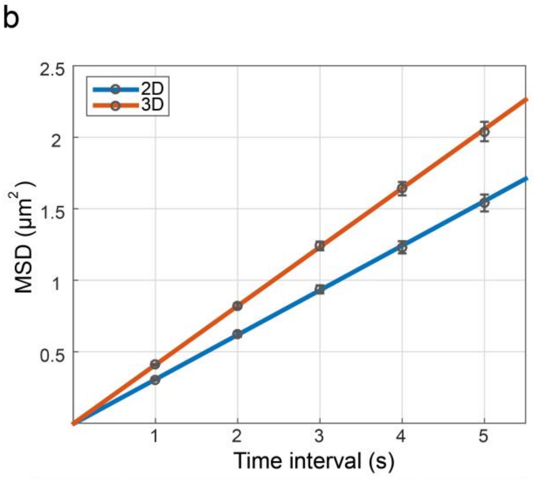

	3D	2D	D_{3D}/D_{2D}
D (µm^2/s)	0.103 ± 0.003	0.078 ± 0.003	1.34 ± 0.02

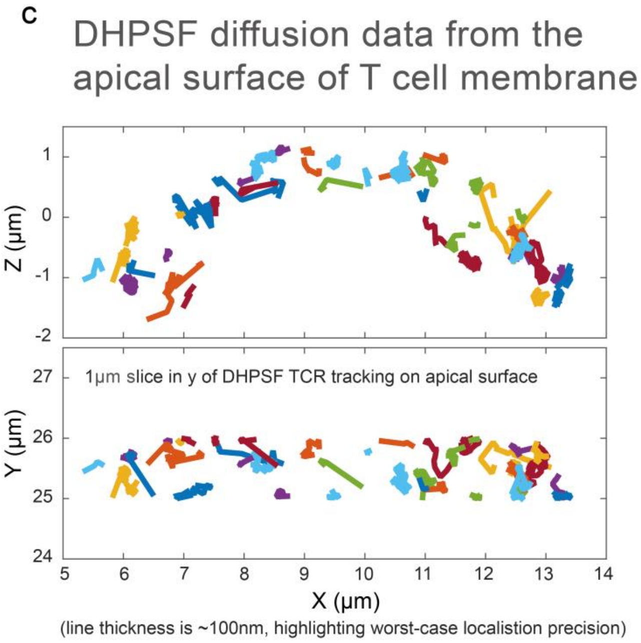

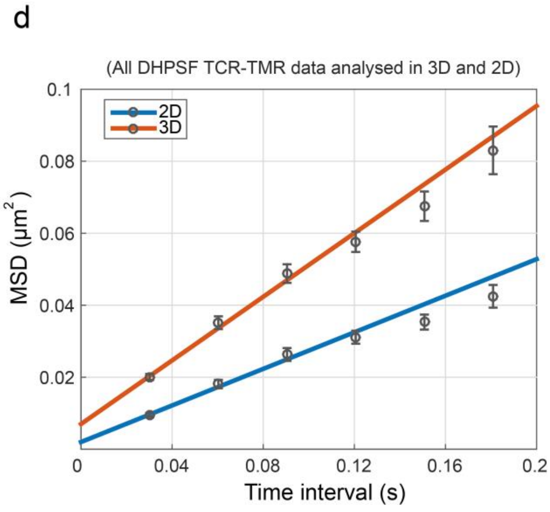

	3D	2D	D_{3D}/D_{2D}
D (µm^2/s)	0.110 ± 0.007	0.0635 ± 0.004	1.73 ± 0.25

Figure S16 2D analysis of 3D diffusion. **(a)** Top-down and side-on views of simulated 3D trajectories on the top surface of a sphere and the corresponding 2D projection. **(b)** MSD plot for simulated diffusion on a spherical surface analysed in 2D (blue) and 3D (red). **(c)** Top-down and side-on views of TCR trajectories from a 1 µm slice of the apical surface of a T cell. The thickness of trajectories is rendered as ~100 nm to represent the worse-case localisation precision of the DHPSF. Trajectories can be seen to exhibit significant radial movement, away from a model sphere. **(d)** MSD plot for TCR diffusion analysed in 2D (blue) and 3D (red).

Supporting References

1. Weimann, L., K.A. Ganzinger, J. McColl, K.L. Irvine, S.J. Davis, N.J. Gay, C.E. Bryant, and D. Klenerman. 2013. A Quantitative Comparison of Single-Dye Tracking Analysis Tools Using Monte Carlo Simulations. PLoS One. 8: e64287.

2. Herbert, A. Single Molecule Light Microscopy ImageJ Plugins. www.sussex.ac.uk/gdsc/intranet/microscopy/imagej/smlm_plugins. .

3. Saxton, M.J. 1997. Single-particle tracking: the distribution of diffusion coefficients. Biophys. J. 72: 1744–1753.

4. Jung, Y., I. Riven, S.W. Feigelson, E. Kartvelishvily, K. Tohya, M. Miyasaka, R. Alon, and G. Haran. 2016. Three-dimensional localization of T-cell receptors in relation to microvilli using a combination of superresolution microscopies. Proc. Natl. Acad. Sci. U. S. A. 113: E5916–E5924.
