# Supplementary material for: Three-Dimensional Super-Resolution in Eukaryotic Cells Using the Double-Helix Point Spread Function
Source: Biophys J. 2017 Apr 11;112(7):1444–54. doi: 10.1016/j.bpj.2017.02.023 (PMC5390298; doi:10.1016/j.bpj.2017.02.023)
Supplement: Document S2. Article plus Supporting Material [file mmc3.pdf]

Biophysical Journal
Article

Biophysical Society

Three-Dimensional Super-Resolution in Eukaryotic Cells Using the Double-Helix Point Spread Function

Alexander R. Carr,[1] Aleks Ponjavic,[1] Srinjan Basu,[2] James McColl,[1] Ana Mafalda Santos,[3] Simon Davis,[3] Ernest D. Laue,[2] David Klenerman,[1] and Steven F. Lee[1,*]
[1]Department of Chemistry and [2]Department of Biochemistry, University of Cambridge, Cambridge, United Kingdom; and [3]Radcliffe Department of Clinical Medicine and Medical Research Council Human Immunology Unit, Weatherall Institute of Molecular Medicine, University of Oxford, Oxford, United Kingdom

ABSTRACT Single-molecule localization microscopy, typically based on total internal reflection illumination, has taken our understanding of protein organization and dynamics in cells beyond the diffraction limit. However, biological systems exist in a complicated three-dimensional environment, which has required the development of new techniques, including the double-helix point spread function (DHPSF), to accurately visualize biological processes. The application of the DHPSF approach has so far been limited to the study of relatively small prokaryotic cells. By matching the refractive index of the objective lens immersion liquid to that of the sample media, we demonstrate DHPSF imaging of up to 15-μm-thick whole eukaryotic cell volumes in three to five imaging planes. We illustrate the capabilities of the DHPSF by exploring large-scale membrane reorganization in human T cells after receptor triggering, and by using single-particle tracking to image several mammalian proteins, including membrane, cytoplasmic, and nuclear proteins in T cells and embryonic stem cells.

INTRODUCTION

Single-molecule imaging (SMI) methods, including super-resolution techniques such as photoactivated localization microscopy (PALM) (1) and (direct) stochastic optical reconstruction microscopy [(d)STORM] (2–4), are widely used to study the dynamics and organization of proteins within cells at a resolution below the diffraction limit (5). The majority of SMI has been carried out using total internal reflection fluorescence microscopy (TIRFM) (6), which is restricted to probing interfaces. Other excitation confinement techniques, such as light-sheet microscopy, are becoming widely used owing to their ability to image above the coverslip with high contrast (7), enabling intracellular imaging and minimizing surface-induced perturbations of protein dynamics (8). Like TIRFM, these methods typically employ two-dimensional (2D) imaging, which complicates the study of curved and irregular structures above the coverslip. This limitation has motivated the development of a number of three-dimensional (3D) SMI techniques, which allow for complete sampling of the protein distribution with high precision in all dimensions. Although many innovative techniques are available to access axial information (9–11), the most commonly implemented are astigmatism (12) and biplane (13). Both of these techniques are capable of achieving ~10 nm lateral and ~20 nm axial localization precision (14), but imaging is currently restricted to a diffraction-limited depth of field of ~500 nm, which complicates the image analysis of large structures. The double-helix point spread function (DHPSF) (15,16) is a widely used example of a rotating PSF whose intensity distributions rotate as they propagate along the optical axis, allowing access to a significantly larger depth of field (~4 μm) (17,18). Whereas biplane and astigmatism have been shown to achieve a greater peak localization precision, one advantage of the DHPSF is that it maintains a more consistent localization precision in all dimensions across the entire depth of field (19). These factors make the DHPSF well suited to super-resolution imaging and single-particle tracking (SPT) in complex biological samples, for both imaging and live-cell single-molecule tracking experiments. In addition to PALM (1), the DHPSF is compatible with any other structurally resolved technique that relies on the localization of isolated emitters, including STORM (2,3), dSTORM (4), and points accumulated for imaging nanoscale topography (PAINT) (20).

3D techniques have been used for SMI in a variety of prokaryotic (21–23) and eukaryotic (24,25) cells. However,

Submitted November 29, 2016, and accepted for publication February 21, 2017.

*Correspondence: sl591@cam.ac.uk

Editor: Antoine van Oijen.
http://dx.doi.org/10.1016/j.bpj.2017.02.023

Crown Copyright © 2017
This is an open access article under the CC BY license (http://creativecommons.org/licenses/by/4.0/).

CrossMark

SMI in eukaryotic cells has proven to be particularly challenging due to their size, which significantly increases autofluorescence and out-of-focus fluorescence. This can be overcome by the use of selective plane illumination techniques (26), at the cost of increased experimental complexity. Scanning many separate image planes is also often required to capture entire cells, complicating the recovery of 3D information. On the other hand, 3D SMI techniques employing a large depth of field, such as DHPSF and multifocal microscopy (MFM) (27,28), require significantly fewer imaging planes to cover thick samples, making these approaches more compatible with whole-cell imaging. However, spherical aberrations typically limit these techniques by degrading the image (29) above the coverslip, making very large volumes difficult to visualize. This effect can be minimized by matching the refractive index of the immersion liquid of the objective lens to the sample medium (30,31), allowing for the DHPSF to be used above the coverslip surface (Figs. S1 and S2 in the Supporting Material).

Dynamic information can also be extracted from SMI by using SPT to determine the diffusion coefficient (32) and stoichiometries (33,34) of protein complexes. SPT has been applied to a variety of eukaryotic cells (35). Most such studies have been conducted in two dimensions using TIRFM, although the use of 3D techniques is emerging, enabling SPT of cytoplasmic and nuclear proteins (36,37). When tracking slower-moving membrane proteins, the narrow depth of field of most techniques is generally not a problem. However, faster-moving proteins are prone to escape the imaging volume, reducing the length of recorded trajectories and therefore potentially compromising the analysis. As a result, imaging of nuclear and cytoplasmic proteins has been problematic due to reduced depths of field when using high-numerical-aperture (high-NA) objective lenses. The large depth of field of the DHPSF combined with a water immersion objective lens allows for imaging of fast-moving proteins with long track lengths throughout cell volumes, making it ideally suited for SPT of a variety of target proteins.

Here, we demonstrate the versatility of the DHPSF in conjunction with a water immersion objective lens for both 3D whole-cell imaging and 3D SPT above coverslip surfaces, which can be performed in technically demanding areas of eukaryotic cells, including apical membranes and inside nuclei. Super-resolved localizations of a membrane protein covering ~10- to 15-μm-thick cell volumes reveal the large-scale receptor reorganization of the membrane of whole T cells.

We demonstrate that the technique can be applied to multiple cell types, including T cells and embryonic stem (ES) cells, enabling new biophysical studies on the 3D organization and dynamics of membrane, cytoplasmic, and nuclear proteins.

MATERIALS AND METHODS

T cell culture

Jurkat T cells (ATCC TIB-152) were transduced via a lentiviral approach to stably express the β-chain of the T cell receptor (TCR), ζ-chain-associated protein kinase (Zap70) HaloTag-tagged proteins, and CD28-mEos3.2 chimeric protein. Jurkat T cells were grown in phenol red-free RPMI supplemented with 10% fetal calf serum (Sigma-Aldrich, Madison, WI), 1% HEPES buffer (Sigma-Aldrich), 1% sodium pyruvate (Sigma-Aldrich), 1% penicillin streptomycin (Sigma-Aldrich), and 2% glutamine (Sigma-Aldrich).

T cell labeling

Before imaging, ~10^6 T cells expressing TCR-β or Zap70 HaloTag-tagged proteins were labeled with HaloLigand-tetramethylrhodamine (TMR; HaloTag TMR Ligand, Promega, Madison, WI) for 30 min at 37°C. The cells were then subjected to three washes in twice-filtered (0.22 μm Millex-GP syringe filter unit, Millipore, Billerica, MA) phosphate-buffered saline (PBS, Life Technologies, Carlsbad, CA) followed by a 30 min incubation in T cell medium (involving centrifugation at 600 $\times$ g, 2 min). The cells were imaged live or were fixed in 4% paraformaldehyde (Sigma-Aldrich) and 0.2% glutaraldehyde (Sigma-Aldrich) for 60 min. Before imaging, the T cells were centrifuged and resuspended in 200 μL of twice-filtered PBS.

T cell coverslip coating

Glass slides (24 $\times$ 50 mm borosilicate, thickness No. 1, Brand, Wertheim, Germany) were cleaned with argon plasma (PDC-002, Harrick Plasma, Ithaca, NY) for 20 min and then coated with poly-L-lysine (molecular mass 150–300 kDa; P4832, Sigma-Aldrich) for 30 min when imaging fixed cells, or nonspecific immunoglobulin G (IgG; Jackson Immunoresearch Europe, Newmarket, UK) for 30 minutes, when imaging live cells. Then, 100 μL of PBS was replaced and 20–100 μL of cells in PBS was added and allowed to settle onto the surface.

To image fixed cells during immunological triggering events, argon-plasma-cleaned slides were coated with activating OKT3 (10 μM/mL; S.D.'s group, Weatherall Institute for Molecular Medicine, University of Oxford) for 20 min. Live cells were added to the surface and allowed to settle for 5 or 10 min before the media was removed and replaced with 200 μL of fixing solution for 60 min. The glass slides were then gently washed with PBS.

ES cell culture

Mouse ES cell lines were cultured in standard serum and mouse leukemia inhibitory factor (mLIF) conditions (Glasgow minimum essential medium (MEM, Sigma-Aldrich) containing 100 mM 2-mercaptoethanol (Life Technologies), 1$\times$ MEM nonessential amino acids (Sigma-Aldrich), 2 mM L-glutamine (Invitrogen, Carlsbad, CA), 1 mM sodium pyruvate (Sigma-Aldrich), 10% fetal bovine serum (HyClone FBS, GE Healthcare, Vienna, Austria), and 10 ng/mL mLIF (provided by the Biochemistry Department, University of Cambridge)). They were passaged every 2 days by washing in PBS (Sigma-Aldrich), adding Trypsin-EDTA 0.25% (Invitrogen) to detach the cells, and then washing in media before replating in fresh media. To help the cells attach to the surface, the plates were incubated for 15 min at room temperature in PBS containing 0.1% gelatin (Sigma-Aldrich).

ES cell labeling

Two days before imaging, cells were passaged onto 35 mm glass-bottom dishes (No. 1.0, MatTek, Ashland, MA) in phenol red-free serum and

mLIF conditions. The cells were labeled with 0.5–5 nM HaloLigand-JF_{549} (38) for at least 15 min, followed by two washes in PBS, and then imaged in phenol red-free serum and mLIF conditions containing 5–10 mM Trolox. For cell fixation, cells were incubated in 1:1 methanol/ethanol for 6 min at −20°C and imaged in PBS containing 10 mM Trolox. The fixed cells were washed with PBS before imaging.

DHPSF microscopy

A custom-made DHPSF microscope was built incorporating a 1.2 NA water immersion objective lens (Plan Apo VC 60×, Nikon, Tokyo, Japan), facilitating imaging above the coverslip surface. The DHPSF transformation was achieved by introducing additional optics into the emission path of a conventional fluorescence microscope (Eclipse Ti-U, Nikon) with the objective lens mounted onto a scanning piezo stage (P-726 PIFOC, PI, Karlsruhe, Germany) (Fig. S3). A 4*f* system of lenses placed at the image plane relayed the image onto an EMCCD detector (Evolve Delta 512, Photometrics, Tucson, AZ). A 580 nm optimized double-helix phase mask (PM) (DoubleHelix, Boulder, CO) placed in the Fourier plane of the 4*f* system performed the DHPSF transformation (Fig. 1 *a*). Excitation and activation illumination was provided by 561 nm (200 mW, Cobolt Jive 100, Cobolt, Solna, Sweden) and 405 nm (120 mW, iBeam smart-405-s, Toptica, Munich, Germany) lasers, respectively, that were circularly polarized, collimated, and focused to the back aperture of the objective lens. The fluorescence signal was then separated from the excitation beams into the emission path by a quad-band dichroic mirror (Di01-R405/488/561/635-25x36, Semrock, Rochester, NY) before being focused into the image plane by a tube lens. Finally, long-pass and band-pass filters (BLP02-561R-25 and FF01-580/14-25, respectively; Semrock) placed immediately before the camera isolated the fluorescence emission.

A range of exposure times were used to image the samples, with 30 ms used to image membrane proteins in SPT and 10–20 ms used to image the faster-diffusing cytoplasmic and nuclear proteins in SPT. An exposure time of 100 ms was used to image fixed cells in whole-cell scanning experiments. When imaging mEos3.2, a continuous 561 nm excitation beam at ~1 kW/cm^2 was used in conjunction with a shutter-pulsed (SC10 shutter controller, ThorLabs, Newton, NJ) 405 nm photoconversion beam at 5 W/cm^2, which photoconverted mEos3.2 molecules across all planes of the sample. When imaging TMR, only a continuous 561 nm excitation beam at ~940 W/cm^2 was used. Specific case-by-case photon values and corresponding localization precisions are included for all presented experiments in Fig. S4.

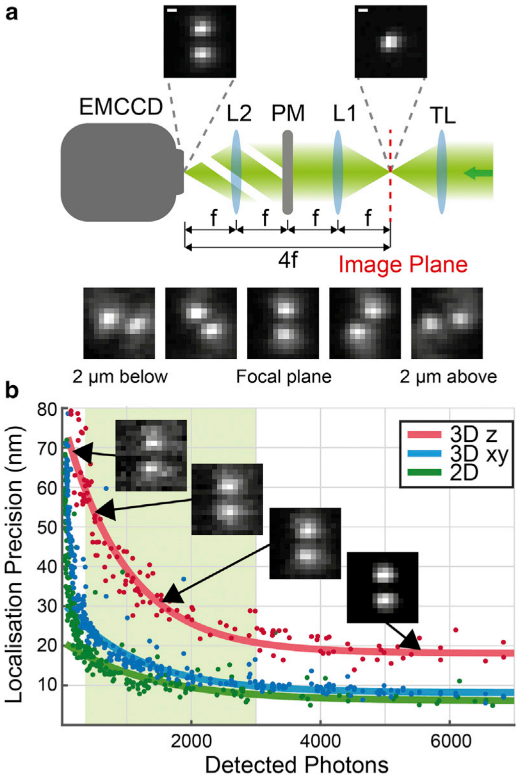

FIGURE 1 Emission path of DHPSF setup and empirically-determined localization precision of the DHPSF as a function of detected photons. (*a*) The DHPSF is implemented by the addition of a 4*f* system comprised of two lenses (L1 & L2) into the emission path of a fluorescence microscope with a DHPSF PM placed in the Fourier-transfer plane of the 4*f* system (*center of the two lenses*). The first plane of the 4*f* system is placed in the image plane of the microscope, relaying the emission signal onto an EMCCD placed a distance 4*f* away. Scale bars are 500 nm. (*b*) Measured lateral (xy) and axial (z) localization precision compared to a 2D fluorescence microscope as a function of detected photons with example DHPSFs. The highlighted region represents typical experimentally determined detected photon numbers when imaging fluorescent proteins and organic dyes. Localization precision was determined by imaging immobilized fluorescent beads.

Fluorescent bead preparation/DHPSF calibration

To calibrate the angle between the lobes in the DHPSF as a function of axial position, 100 μL of an ~3.6 × 10^8 particles/mL solution of fluorescent beads (0.1 μm, TetraSpeck microspheres, ThermoFisher, Waltham, MA) in PBS was imaged on a surface. Microscope slides cleaned with argon plasma (PDC-002, Harrick Plasma) were coated with poly-L-lysine for 30 min and washed with PBS before adding the diluted beads. After 5 min at room temperature, the slides were washed in PBS and imaged using the DHPSF instrument. The piezo stage was used to scan the objective lens axially through the sample in 50 nm steps over 4 μm, recording 10 30-ms exposures at each step.

Determination of localization precision

The DHPSF microscope calibration slides were imaged for 2000 frames with 30 ms exposure, with and without the inclusion of the PM in the imaging path. A range of excitation powers was used such that the emission signal of the beads covered the dynamic range of the EMCCD. The localization precision was determined using a previously described method (23). Beads were localized using PeakFit (GDSC SMLM single-molecule plugins (39)) for 2D images and easy-DHPSF (40) for DHPSF microscopy images, and the distribution of localized positions for each bead was analyzed to determine the localization precision of the instrument as a whole. An exponential decay was fit to each data set to guide the eye.

Lateral and axial localization precision was measured at ~10–25 nm and ~20–60 nm, respectively, in typical measured photon ranges (Fig. S4) when imaging fluorescent proteins or organic dyes (Fig. 1 *b*). Typical photon values for each experiment are listed in Table S1.

Whole-cell scanning

To image whole cells, we scanned the focal plane axially through the cells in 3 μm steps via the piezo-mounted objective, acquiring 100 images at each position before moving to the next position. This whole process was repeated until no more localizations were observed, typically 50 times (~20,000 frames). Between 1000 and 5000 localizations were collected for each cell depending on the experiment. By offsetting the recorded localizations from different imaging planes, a super-resolution whole-cell map

of the tagged protein was produced. Typically, for a 10–15 μm cell, three to five overlapping planes were collected. The collection efficiency profile of the objective lens causes a reduction in recorded localization density at the periphery of the depth of field (Fig. S5, *a* and *b*). We have experimentally determined that successive imaging planes should be offset by ~3 μm or 75% of the working depth of field to achieve the flattest localization density across multiple imaging planes (Fig. S5 *c*). We determined the sample drift and lateral offset between imaging planes by imaging fluorescent beads (Figs. S6 and S7), and found them to be small compared with other error sources in the presented quantitative analysis (a maximum of 89 $\pm$ 8 nm and 45.5 $\pm$ 22 nm).

Mesh fitting

Using a standard method and functions included in Meshlab open source software, (http://meshlab.sourceforge.net), the 3D localization data were converted into an object mesh. For each point, 50–200 neighbors (depending on the localization density) were considered to estimate a normal. These points and their normals were then used to build a surface using the Poisson surface reconstruction approach (41,42), which solves an approximate indicator function of the object by fitting its gradient to the input normal. Finally, meshes were uniformly sampled, creating an even distribution of vertices (see Fig. S8 for detailed instructions).

Cell-specific complete spatial randomness model

An algorithm to create a complete spatial randomness (CSR) model from a given cell mesh was written in MATLAB (The MathWorks, Natick, MA). A number of vertices equal to the number of unique proteins localized from the cell were sampled randomly from the mesh. The select vertices were then displaced by a random distance between −250 and 250 nm in all three dimensions, representing the approximate precision of the fitted mesh (~500 nm). An additional displacement was sampled from a normal distribution in all dimensions to represent the reconstruction error. This displacement was centered at the recorded localization precisions for the number of photons detected in the corresponding experiment. These vertices could then be used as model localization data in quantitative analysis, representing the position of proteins on the outer cell membrane.

Interprotein distance analysis

An algorithm to analyze the interprotein distances of 3D localization data was written in MATLAB. First, duplicate localizations originating from repeat localizations of the same fluorophore were removed by filtering for nearby recorded events in space (<500 nm) and time (<1 s). For each remaining localization, the distance to all other localizations was calculated and used to create a histogram. The peak value of this histogram, representing the median interprotein distance, was then recorded for each localization.

The effect of cell morphology was corrected for by finding the deviation from the cell-specific CSR model localizations. The interprotein distance analysis was conducted on 1000 instances of model localization data for each cell, taking the mean and the 5th and 95th percentiles to create error limits. The mean deviation of the true interprotein distance from the model interprotein distance provides information about how well the CSR model fits as a whole. The distribution of median interprotein distance as a function of axial position was also investigated by taking the mean value in 500 nm slices axially.

Diffusion analysis

An algorithm to perform mean-square displacement (MSD) analysis using 3D localization data was written in MATLAB by extending a previously published method (43) to three dimensions. The performance of the algorithm was benchmarked using simulated data and by comparing the results from 2D data with those produced by the parent algorithm (Fig. S9).

To determine the diffusion coefficient of imaged proteins, an ensemble MSD curve was created by taking the mean MSD of the trajectory data at each time interval given by

$$MSD(n\Delta t) = \frac{1}{l-n}\sum_{i=1}^{l-n}[x(i+n)-x(i)]^2 + [y(i+n)-y(i)]^2 + [z(i+n)-z(i)]^2, \quad (1)$$

where Δt denotes the time step between frames, l the trajectory length, and $x(i)$, $y(i)$, and $z(i)$ the position of the particle in frame i. The gradient of a linear fit to the first four points of the curve was divided by twice the number of dimensions of diffusion (two for membrane-bound diffusion and three for intracellular diffusion) to give the diffusion coefficient (32,44), with the error in fit determining the calculated error in the mean diffusion coefficient:

$$MSD(n\Delta t) = qDn\Delta t + C, \quad (2)$$

where q is given by twice the number of dimensions of diffusion, D denotes the diffusion coefficient, and C is the offset.

To separate bound and unbound trajectories, MSD plots were created for individual trajectories and fit to a straight line. The R-squared value of this fit was used to threshold bound and unbound trajectories. An R-squared value of 0.85 was determined by quantitative analysis of simulated data using empirically determined parameters, including localization precision, and then verified with experimental data (Fig. S10).

RESULTS

DHPSF whole-cell super-resolution imaging

Whole-cell super-resolution imaging was achieved by scanning a water immersion objective lens in three to five axial planes (Fig. 2 *a*; Materials and Methods). This allowed DHPSF imaging of a variety of T cells stably expressing fluorescently tagged forms of two membrane receptors with multiple labeling strategies, including CD28-mEos3.2 in a PALM mode and low-concentration labeling of TCR-HaloTag-TMR. As expected, fixed Jurkat cells appeared predominantly spherical (Fig. 2 *b*), with no large systematic differences in shape for all cells imaged (see Fig. S11 for other examples). In addition, the localization precision (~10–25 nm laterally and ~20–60 nm axially; Fig. 1 *b*) allowed for receptors to be resolved well below the diffraction limit (Fig. 2 *c*). The distribution of nearest-neighbor distances between CD28 molecules revealed that 27% were too close to be resolved by standard confocal microscopy (<250 nm apart) and 63% were closer than 500 nm apart (Fig. S12).

Visualizing and quantifying large-scale reorganization over whole cells

T cells are known to undergo large-scale spatial reorganizations when activated, presumably to maximize contact with

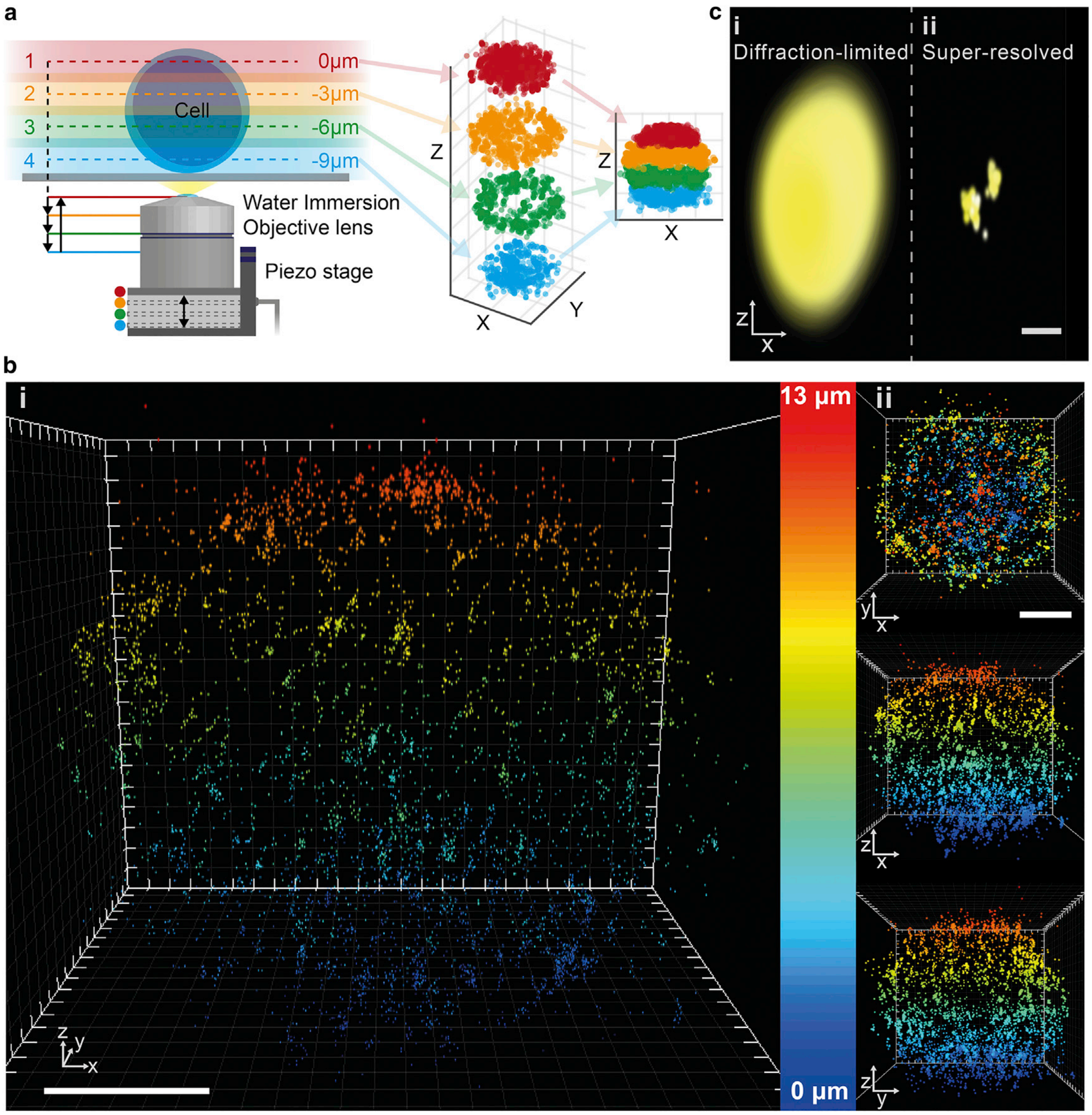

FIGURE 2 Super-resolved whole-cell reconstruction of a T cell using the DHPSF. (*a*) Experimental procedure for whole-cell scanning. A 60x 1.2NA water immersion objective lens was mounted on a piezo stage. Localizations were recorded from 3–5 overlapping imaging planes throughout the cell volume before being combined to form the final whole-cell image. (*b*) Super-resolved whole-cell reconstruction of CD28 membrane protein on a Jurkat T cell (4810 localisations). Jurkat cells expressing CD28-mEos3.2 were imaged with the DHPSF. Localizations are color coded by axial height from the coverslip and rendered with isotropic 3D Gaussians with size determined by experimentally measured localization precision for ~350 photons detected (Figure S3) (*b i*). (*b ii*) Top-down and side-on views of (*b i*) rendered with 100 nm localization precision for visibility. Scale bars are 4 μm. (*b*) and (*c*) were plotted using ViSP localization visualization software (71). (*c*) Comparison between diffraction-limited (*c i*) and super-resolved (25 nm lateral precision and 50 nm axial precision) (*c ii*) rendering of two clusters of localizations originating from two individual TCR proteins separated by ~300 nm. Scale bar is 300 nm. Jurkat cells expressing TCR-HaloTag were labeled with TMR-HaloTag ligand and imaged with the DHPSF.

the target antigen-presenting cell (45). A key protein in this process is the TCR, which binds to major histocompatibility proteins that are expressed by the antigen-presenting cell and present antigenic peptides, resulting in T cell activation (46–48). DHPSF imaging was used to examine and quantify the distribution of the TCR on individual T cells. By fitting meshes to these sparse localization data, the mean position of the plasma membrane of each T cell was extracted and used to visualize large-scale morphological changes at three distinct time points during activation (Fig. 3, *a–c*). As the fitted meshes served to report the average position of the outer membrane, some proteins were localized >1 μm away from the mesh, corresponding to large pseudopodia and internal stores.

Substantial differences in morphology were observed between resting cells fixed in suspension versus those fixed after contacting an activating antibody (OKT3)-coated surface. In suspension, the cells were largely spherical, whereas cells that had been in contact with an activating surface for 5 min exhibited flattening and extension of the basal

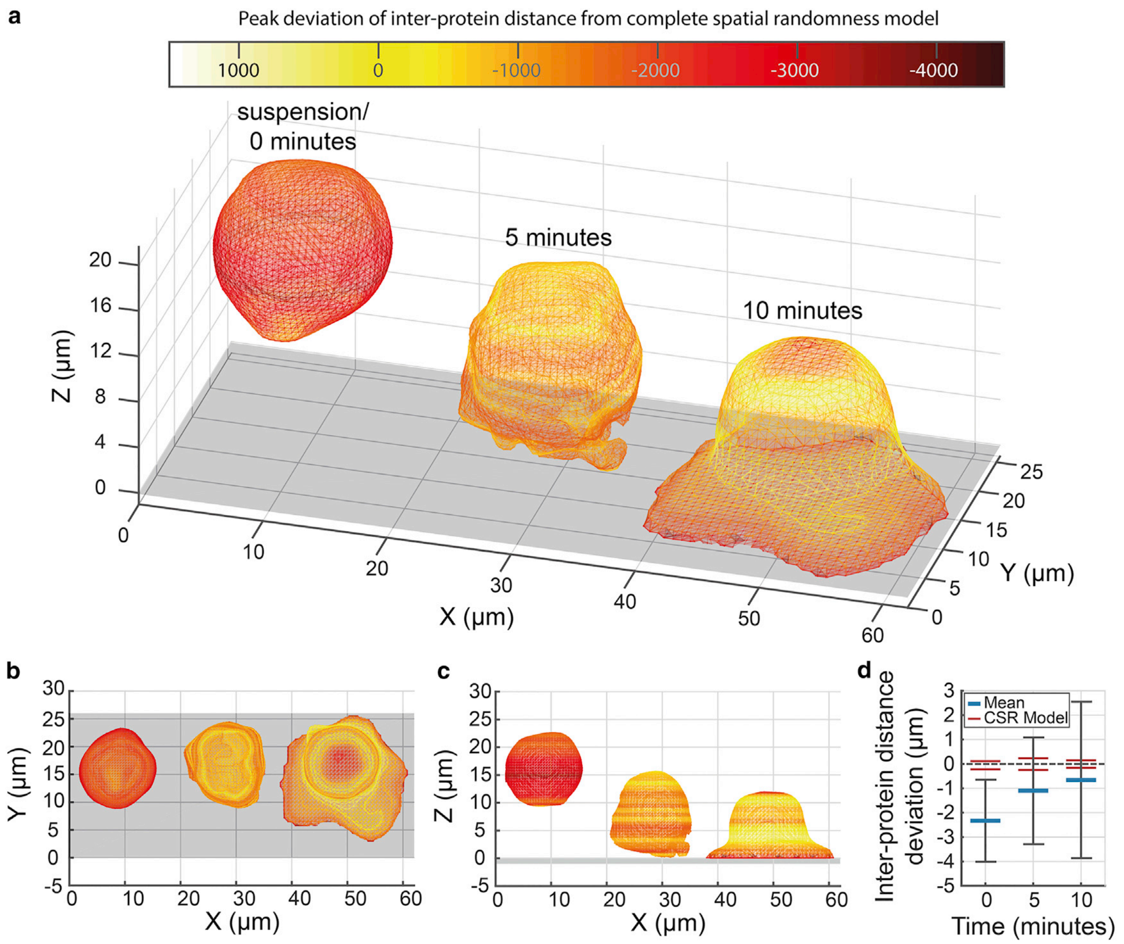

FIGURE 3 Visualization of large-scale reorganization of the outer membrane of Jurkat T cells on activating coated surfaces by mesh fitting to 3D localization data. (*a*) Meshes were fit to DHPSF whole-cell TCR localization data of three Jurkat T cells tagged with HaloTag-TMR. Cells were fixed in solution (*left*, 1149 localizations), 5 minutes (*center*, 2495 localizations), or 10 minutes (*right*, 2397 localisations) after being dropped onto an activating OKT3 coated surface. Large-scale reorganization of the outer membrane caused by immune-response triggering and the formation of an immunological synapse is visualized at different stages. Cells are colored axially by deviation of inter-protein distance from a complete spatial randomness model of a membrane-bound protein with yellow, which indicates no deviation from model system, and red/black, which indicates a reduction in peak inter-protein distance compared to model system. The flat grey surface represents the coverslip of the experiment. The cell fixed in solution (*left*) has been rendered away from the coverslip to emphasize the resting state. (*b–c*) Top-down and side-on views of (*a*). (*d*) Mean inter-protein distance deviation of each cell shown in (*a*) from a complete spatial randomness model of a membrane-bound protein.

membrane close to the surface. Much larger membrane spreading was evident for cells contacting the surface for 10 min.

For the three time points, the vertex densities in the final cell meshes were 5.1, 4.9, and 2.8 vertices/μm^2 with 0.4, 0.7, and 0.8 localizations per vertex, respectively.

Furthermore, it is possible to decouple protein reorganization from morphological changes by using quantitative analysis (Fig. S13). Initially, the mean interprotein distance distribution of the TCR was computed across whole cells, and by creating cell-specific volumes, this distribution was compared with a model of protein positioned randomly on the outer membrane (see Materials and Methods for a detailed discussion). The interprotein distance distribution of the TCR in the resting cell was found to be significantly different from that predicted by the membrane-bound CSR model. In contrast, the TCR distribution in the activated cells (after 5 and 10 min) overlapped substantially with the analogous CSR models (Fig. 3 *d*). Deviation in the inter-TCR distance was not observed to change significantly with axial depth for any of the cells analyzed (Fig. S13, *e–g*).

DHPSF-SPT on the apical surface and in the cytoplasm of T cells

Membrane-bound and cytoplasmic T cell proteins were imaged on the top ~4 μm of Jurkat T cells attached to IgG-coated coverslips (Fig. 4 *a*). The membrane-associated TCR, labeled with HaloTag-TMR, was imaged as it diffused over the nanostructured outer membrane of live Jurkat T cells, and the intracellular protein Zap70, also labeled with HaloTag-TMR, was imaged inside live Jurkat

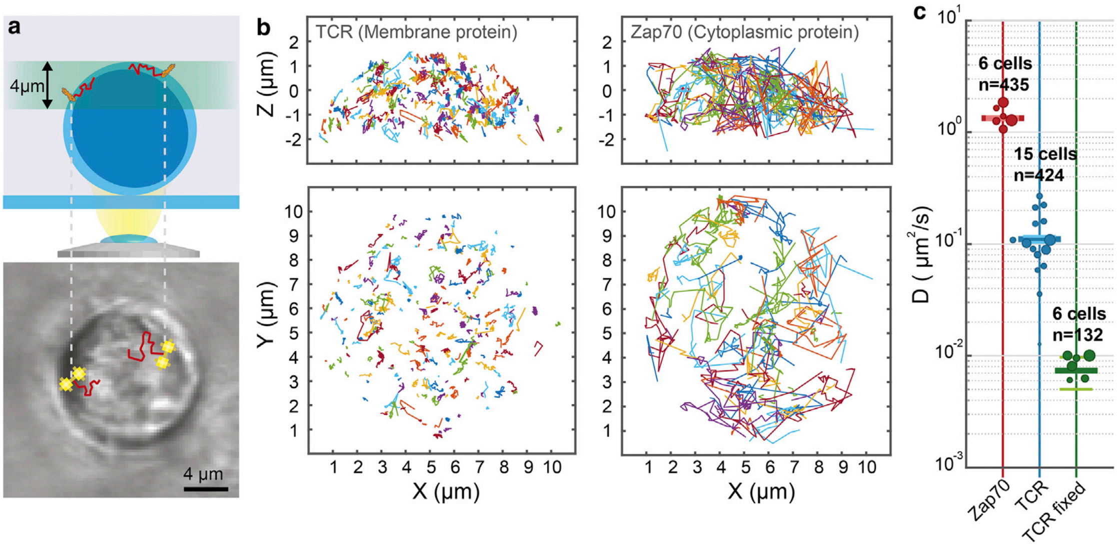

FIGURE 4 SPT of membrane and cytoplasmic proteins using the DHPSF in live human T cells. (*a*) Experimental procedure for SPT at the apical surface using the DHPSF. Jurkat T cells were labeled with HaloTag-TMR and dropped onto passivating-coated coverlips. A 60x water immersion objective was focused at the apical surface to image the membrane protein TCR or the cytoplasmic protein Zap70. TCR was also imaged in fixed cells. (*b*) Representative side-on (*top*) and top-down (*bottom*) views of trajectories of unfixed TCR (*left*) and Zap70 (*right*) proteins at the apical surface. The difference in speed between the two proteins can be clearly seen in the length of trajectories. (*c*) Mean diffusion coefficient for Zap70 and TCR live and fixed determined by MSD analysis (*horizontal bars*) with cell-to-cell variation (*circles*) and total number of trajectories. The size of the circles is proportional to the number of tracks obtained from the cell. Small circles represent cells with fewer trajectories while large circles represent cells with many trajectories. The number of tracks ranges from ~30-140 per cell for Zap70 and ~5-140 for TCR.

T cells (Fig. 4 *b*). MSD analysis revealed that the TCR had a mean diffusion coefficient of 0.110 ± 0.007 $\mu m^2/s$ (424 trajectories, 15 cells), whereas Zap70 had a significantly faster mean diffusion coefficient of 1.34 ± 0.04 $\mu m^2/s$ (435 trajectories, six cells) (Fig. 4 *c*). The precision in diffusion coefficient measurements was estimated by MSD analysis of static TCR in fixed cells and found to be 0.008 ± 0.002 $\mu m^2/s$ (132 trajectories, six cells), agreeing well with the observed errors in live-cell TCR experiments.

The MSD plot for the TCR fits well to a straight line, indicating free diffusion, whereas the plot for Zap70 drops below the straight line fitted to the first four points, indicating a degree of confinement (32) (Fig. S14).

DHPSF-SPT in the nucleus of ES cells

We investigated the chromatin remodeler CHD4, a nuclear protein that is known to play a critical role in ES cell pluripotency as part of the larger nucleosome remodeling and deacetylase complex, as well as in the DNA damage response and cell-cycle regulation (49). CHD4 was previously shown to occupy two diffusion states in near-equal ratios: 1) bound to chromatin and 2) a fast-moving state (50). It was reported that removal of the nuclear protein Methyl-CpG Binding Domain Protein 3 (MBD3) resulted in an ~25% increase in the diffusion coefficient of the fast-moving population of CHD4 compared with wild-type (WT) cells (50). We used the DHPSF to track nuclear CHD4 tagged with HaloTag-JF_{549} in WT mouse ES cells and MBD3 null mouse ES cells (Fig. 5, *a* and *b*).

The mean diffusion coefficient of unbound CHD4 was determined to be 0.60 ± 0.01 $\mu m^2/s$ in wild-type cells (851 trajectories, 58 cells) and 0.75 ± 0.03 $\mu m^2/s$ in MBD3 null cells (1212 trajectories, 26 cells), agreeing with previous observations (50). Both MSD plots exhibited linearity for the first four points (Fig. 5 *c*), as observed for measurements of Zap70 diffusion.

DISCUSSION

Whole-cell imaging with the DHPSF

The DHPSF facilitates the probing of complex structures in the intricate 3D environment of the cell nucleus and the apical cell surface. Due to its large depth of field, super-resolution, whole-cell images of ~10- to 15-μm-thick samples can be collected in as few as three to five imaging planes. Reducing the number of required imaging planes is beneficial not only because it reduces experimental complexity but also because it allows one to image a greater fraction of emitting fluorophores simultaneously when using excitation geometries that excite all axial planes (i.e., epifluorescence or highly inclined and laminated optical sheet) (51). This is highly relevant when imaging low-density samples such as weakly expressing proteins (e.g., the TCR). Comparable sectioning of whole cells has previously been demonstrated with other 3D imaging methods (28,52).

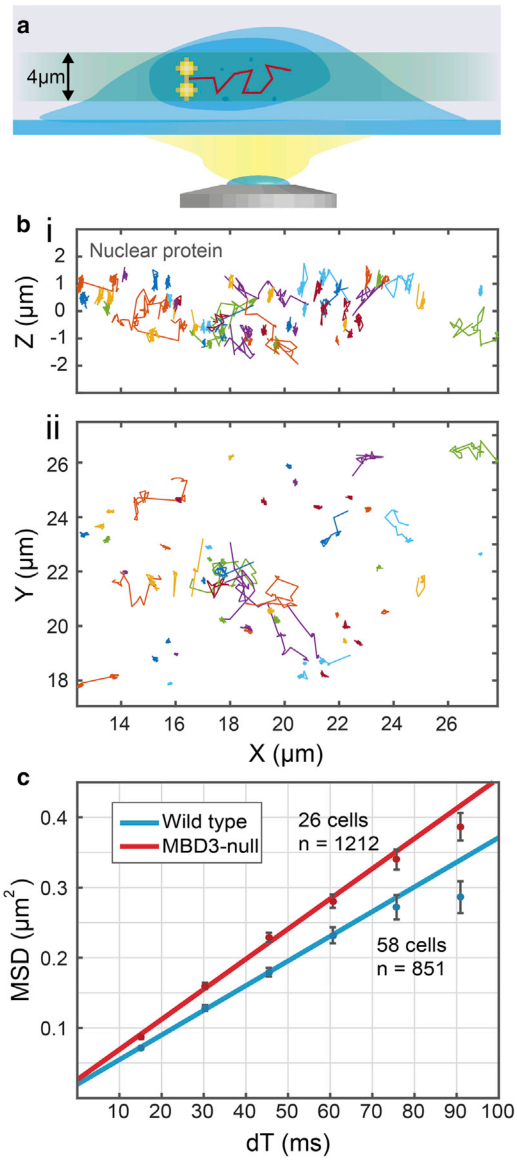

FIGURE 5 SPT of CHD4 nuclear protein using the DHPSF in live mouse ES cells. (*a*) Experimental procedure for SPT in the nucleus of ES cells using the DHPSF. CHD4 was labelled with HaloTag-JF549 in WT cells and MBD3 null mutants and imaged in a ~4 μm-thick plane above the coverslip, within the nucleus of ES cells. (*b*) Side-on (*i*) and top-down (*ii*) view of CHD4 trajectories from a single ES nucleus. The bound and unbound states of CHD4 can clearly be seen. (*c*) MSD plot for the unbound trajectories in WT cells and MBD3 null cells with linear fits to the first 4 points.

Visualizing morphology and quantifying protein reorganization

Fitting meshes to localization data facilitates the visualization of larger morphological changes in the outer membrane of cells as compared with rendering individual localizations, as the eye is no longer drawn to the variations in localization density that are intrinsic to pointillism-based imaging techniques. The meshes report the weighted position of outer localizations, emphasizing the cell membrane and reducing the visual impact of localizations arising from intracellular sources (53).

The precision of the fitted mesh is primarily determined by the sampling density of the localization data. However, even at relatively low sampling densities, large-scale membrane reorganization can be observed and quantitatively analyzed. Mesh fitting is also compatible with super-resolution membrane imaging and visualization, providing adequate sampling density. This is possible in imaging strategies with large localization numbers, such as PAINT (20), which can achieve 10^6–10^9 localizations from a single cell with sufficiently long imaging periods (54).

The reorganization shown in Fig. 3 is likely to be an active process as the T cell attempts to form an immunological synapse (55,56). The peak interprotein distance of the TCR in the resting cell was found to be smaller than predicted by the corresponding membrane-only model system, implying that a significant fraction of the TCR was localized intracellularly. However, the distribution of peak inter-TCR distance in activated cells overlapped with their model systems, indicating a reduction in the intracellular fraction. This observation is supported by an additional analysis of membrane-bound and cytoplasmic TCR fractions, in which TCR molecules that localized within the mesh by >1 μm were considered as intracellular (Fig. S15). In the resting cell, 30% of TCR molecules were localized in the cytoplasm, whereas 17% and 13% of TCR molecules were localized in the cytoplasm in both activated cells (5 and 10 min, respectively). In the resting cell, 65% of TCR molecules were localized within 1 μm of the mesh, whereas 72% and 74% were localized within 1 μm of the mesh in both activated cells (5 and 10 min, respectively). These data are consistent with the notion that immunological stimulation causes intracellular TCRs to be recruited to the outer membrane in Jurkat T cells. One possible mechanism for this is that the TCR is stored in microvesicles in the resting cell, and T cell signaling results in the release of these stored TCRs at the cell surface (57,58). No axial dependence of the distribution of interprotein distance of the TCR was seen, implying that the changes occur across the entire cell rather than being part of a directed process. This analysis nevertheless demonstrates that the DHPSF can resolve relatively small redistributions of molecules across whole cells, such as the transfer of proteins from the cytosol to the cell surface. In the case of low-expressing proteins (e.g., the TCR), a small number of molecules moving from the cell cytoplasm to the cell membrane could significantly affect the overall distribution.

SPT of proteins on the apical surface and in the cytoplasm of T cells

The TCR diffusion rates are significantly higher than suggested by literature values obtained from the apical surface

of Jurkat T cells, which James et al. (59) measured to be 0.06 ± 0.01 $\mu m^2/s$ using fluorescence correlation spectroscopy. No value for cytoplasmic Zap70 could be found for comparison.

Analysis of a 2D *x-y* projection of the 3D data produces a diffusion coefficient of 0.064 ± 0.004 $\mu m^2/s$ for the TCR (Fig. S16 *d*), in good agreement with the literature values. This difference in extracted diffusion coefficient, from three dimensions to two dimensions, was found to be greater than predicted by simulating trajectories on the apical surface of a spherical membrane alone (a factor of 1.72 ± 0.22 difference was seen compared with the predicted factor of 1.34 ± 0.02; Fig. S16 *b*). This is due to trajectories displaying a radial component as well as the angular component expected from a spherical surface (highlighted in Fig. S16 *c*). This is most likely caused by pseudopodia or surface ruffling present on the plasma membrane (60,61). A previous analysis of T cell morphology using electron microscopy determined a roughness factor of 1.8 (62), which has since been used to correct for 3D effects in T cell membrane protein studies (63,64). This represents a case where 3D SPT is essential for accurately studying protein dynamics in complex 3D environments.

SPT of proteins in the nucleus of ES cells

An increase in the diffusion coefficient of CHD4 in the absence of MBD3 compared with wild-type cells was seen, confirming observations made in a previous 2D study (50). The earlier study of CHD4 was not able to utilize MSD analysis because the fast diffusion led to short recorded trajectories, primarily due to the small nominal focal plane (~500 nm) in conventional 2D SPT. Due to the large depth of field afforded by the DHPSF, longer trajectories could be recorded and thus MSD analysis could show that the fast fraction of CHD4 is largely freely diffusing within the nucleus. This example confirms that the DHPSF can be used to study proteins exhibiting a variety of diffusion states across a large dynamic range of diffusion coefficients.

Advantages and disadvantages of the DHPSF

As we have shown, the DHPSF can be used to perform 3D SPT in two key areas in which 2D methods typically perform poorly: 1) on apical cell surfaces and 2) in the nuclei of living cells. Even when imaging membrane phenomena on glass surfaces, the presence of membrane ruffles could make a significant axial contribution to observable behaviors (60,65).

The increased depth of field of the DHPSF (~4 μm) compared with the majority of 3D single-molecule SPT techniques (e.g., astigmatism and biplane) also has the advantage of capturing extended trajectories, as the fluorescent molecules are less likely to leave the imaging volume. This allows for more robust quantitation in standard analysis tools such as MSD, making the DHPSF well suited for tracking a large range of proteins throughout the cell, particularly fast-moving cytoplasmic and nuclear proteins. Other 3D tracking techniques, such as the emerging MFM, have demonstrated a similar depth of field and axial localization precision (27).

The primary disadvantage of the DHPSF is the increased size of the PSF versus other methods. As with the majority of 3D localization techniques, the PSF occupies more area on the detector in the conjugate image plane. In the case of the DHPSF, the area is ~5-fold larger compared with the analogous 2D experiment. Consequently, labeling concentrations should be reduced accordingly, which increases the experimental acquisition time. Secondary issues are the reduction in signal originating from the DHPSF and inefficiencies in the DHPSF PM and additional optics. As the photons comprising the standard 2D PSF are split into two separately localized lobes, the apparent fluorophore intensity is reduced (~43% of 2D PSF photons are observed in each lobe). Furthermore, the use of a low-NA water immersion objective lens not only reduces collection efficiency compared with oil immersion objectives but also causes background signal to be collected from a larger volume. Therefore, samples with a low signal/noise ratio can be more difficult to image, requiring careful experimental optimization and design. Fluorescence-background-reduction techniques such as selective plane illumination could be employed to increase signal/noise ratios (66) and offset this effect. The large depth of field of DHPSF is ideally suited to implementing simple light-sheet systems that typically have a thickness of a few microns (67). Light-sheet microscopy has been combined with the DHPSF to increase signal/noise ratios (68), but this approach has not yet been applied to cell imaging.

CONCLUSIONS

3D super-resolution imaging and SPT have become vital biophysical tools for understanding the organization and dynamics of membrane and intracellular proteins. Using the DHPSF, we were able to super-resolve the position of membrane proteins across whole cells as well as provide quantitative information about their distribution. This approach could potentially be used to elucidate biophysical phenomena related to the nanoscale organization of proteins, such as the recruitment of intracellular TCR to the outer membrane, the role played by TCR clustering in the adaptive immune response (69), and nuclear protein organization in ES cells (70). Large-scale spatial reorganization could be quantified at the single-molecule level and visualized by fitting the superresolved localizations of membrane proteins with a surface mesh. The diffusion coefficients measured and observations made using the DHPSF for SPT agree well with previously published findings. Ultimately, we demonstrate the first, to our knowledge, implementation of the DHPSF

in eukaryotic cells, and show that the technique is compatible with both super-resolution imaging and SPT for studying protein dynamics with super-localise spatial resolution in all three dimensions (71).

SUPPORTING MATERIAL

Supporting Materials and Methods, Supporting Data, sixteen figures, and one table are available at http://www.biophysj.org/biophysj/supplemental/S0006-3495(17)30237-0.

AUTHOR CONTRIBUTIONS

S.F.L. and A.R.C. designed the experimental plan. A.R.C. performed experiments, analyzed the data, and wrote the article. A.P. provided code for simulating tracks on a spherical membrane. S.B. provided and labeled ES cells. J.M. maintained and labeled Jurkat T cells. A.M.S. and S.D.'s lab provided all T cell lines. All authors contributed to the writing of the manuscript and helpful discussion.

ACKNOWLEDGMENTS

We thank Luke Lavis for providing JF_{549} fluorescent dye for use in the ES cell experiments. We thank the Royal Society for the University Research Fellowship of S.F.L. (UF120277). This work was kindly funded by the Engineering and Physical Sciences Research Council (EP/M003663/1) and by the Wellcome Trust.

REFERENCES

1. Betzig, E., G. H. Patterson, …, H. F. Hess. 2006. Imaging intracellular fluorescent proteins at nanometer resolution. *Science.* 313:1642–1645.
2. Rust, M. J., M. Bates, and X. Zhuang. 2006. Sub-diffraction-limit imaging by stochastic optical reconstruction microscopy (STORM). *Nat. Methods.* 3:793–795.
3. Bates, M., B. Huang, …, X. Zhuang. 2007. S-Multicolor super-resolution imaging with photo-switchable fluorescent probes. *Science.* 317:1749–1753.
4. Heilemann, M., S. van de Linde, …, M. Sauer. 2008. Subdiffraction-resolution fluorescence imaging with conventional fluorescent probes. *Angew. Chem. Int. Ed. Engl.* 47:6172–6176.
5. Lord, S. J., H. L. Lee, and W. E. Moerner. 2010. Single-molecule spectroscopy and imaging of biomolecules in living cells. *Anal. Chem.* 82:2192–2203.
6. Mattheyses, A. L., S. M. Simon, and J. Z. Rappoport. 2010. Imaging with total internal reflection fluorescence microscopy for the cell biologist. *J. Cell Sci.* 123:3621–3628.
7. Hu, Y. S., M. Zimmerley, …, H. Cang. 2014. Single-molecule super-resolution light-sheet microscopy. *ChemPhysChem.* 15:577–586.
8. James, J. R., J. McColl, …, S. J. Davis. 2011. The T cell receptor triggering apparatus is composed of monovalent or monomeric proteins. *J. Biol. Chem.* 286:31993–32001.
9. Jia, S., J. C. Vaughan, and X. Zhuang. 2014. Isotropic 3D super-resolution imaging with a self-bending point spread function. *Nat. Photonics.* 8:302–306.
10. Yang, Z., M. Prokopas, …, K. Dholakia. 2014. A compact Airy beam light sheet microscope with a tilted cylindrical lens. *Biomed. Opt. Express.* 5:3434–3442.
11. Shechtman, Y., L. E. Weiss, …, W. E. Moerner. 2015. Precise three-dimensional scan-free multiple-particle tracking over large axial ranges with tetrapod point spread functions. *Nano Lett.* 15:4194–4199.
12. Huang, B., W. Wang, …, X. Zhuang. 2008. Three-dimensional super-resolution imaging by stochastic optical reconstruction microscopy. *Science.* 319:810–813.
13. Juette, M. F., T. J. Gould, …, J. Bewersdorf. 2008. Three-dimensional sub-100 nm resolution fluorescence microscopy of thick samples. *Nat. Methods.* 5:527–529.
14. Horrocks, M. H., M. Palayret, …, S. F. Lee. 2014. The changing point-spread function: single-molecule-based super-resolution imaging. *Histochem. Cell Biol.* 141:577–585.
15. Pavani, S. R. P., M. A. Thompson, …, W. E. Moerner. 2009. Three-dimensional, single-molecule fluorescence imaging beyond the diffraction limit by using a double-helix point spread function. *Proc. Natl. Acad. Sci. USA.* 106:2995–2999.
16. Thompson, M. A., M. D. Lew, …, W. E. Moerner. 2010. Localizing and tracking single nanoscale emitters in three dimensions with high spatiotemporal resolution using a double-helix point spread function. *Nano Lett.* 10:211–218.
17. Lew, M. D., S. F. Lee, …, W. E. Moerner. 2011. Corkscrew point spread function for far-field three-dimensional nanoscale localization of point-like objects. *Opt. Lett.* 36:202–204.
18. Shechtman, Y., S. J. Sahl, …, W. E. Moerner. 2014. Optimal point spread function design for 3D imaging. *Phys. Rev. Lett.* 113:133902.
19. Badieirostami, M., M. D. Lew, …, W. E. Moerner. 2010. Three-dimensional localization precision of the double-helix point spread function versus astigmatism and biplane. *Appl. Phys. Lett.* 97:161103.
20. Sharonov, A., and R. M. Hochstrasser. 2006. Wide-field subdiffraction imaging by accumulated binding of diffusing probes. *Proc. Natl. Acad. Sci. USA.* 103:18911–18916.
21. Kanchanawong, P., and C. M. Waterman. 2012. Advances in light-based imaging of three-dimensional cellular ultrastructure. *Curr. Opin. Cell Biol.* 24:125–133.
22. Lew, M. D., S. F. Lee, …, W. E. Moerner. 2011. Three-dimensional superresolution colocalization of intracellular protein superstructures and the cell surface in live Caulobacter crescentus. *Proc. Natl. Acad. Sci. USA.* 108:E1102–E1110.
23. Gahlmann, A., J. L. Ptacin, …, W. E. Moerner. 2013. Quantitative multicolor subdiffraction imaging of bacterial protein ultrastructures in three dimensions. *Nano Lett.* 13:987–993.
24. Li, D., L. Shao, …, E. Betzig. 2015. Extended-resolution structured illumination imaging of endocytic and cytoskeletal dynamics. *Science.* 349:aab3500.
25. Liu, Z., W. R. Legant, …, R. Tjian. 2014. 3D imaging of Sox2 enhancer clusters in embryonic stem cells. *eLife.* 3:e04236.
26. Liu, Z., L. D. Lavis, and E. Betzig. 2015. Imaging live-cell dynamics and structure at the single-molecule level. *Mol. Cell.* 58:644–659.
27. Abrahamsson, S., J. Chen, …, M. G. L. Gustafsson. 2013. Fast multicolor 3D imaging using aberration-corrected multifocus microscopy. *Nat. Methods.* 10:60–63.
28. Hajj, B., J. Wisniewski, …, M. Dahan. 2014. Whole-cell, multicolor superresolution imaging using volumetric multifocus microscopy. *Proc. Natl. Acad. Sci. USA.* 111:17480–17485.
29. Ghosh, S., and C. Preza. 2013. Characterization of a three-dimensional double-helix point-spread function for fluorescence microscopy in the presence of spherical aberration. *J. Biomed. Opt.* 18:036010.
30. Hell, S., G. Reiner, …, E. H. K. Stelzer. 1993. Aberrations in confocal fluorescence microscopy induced by mismatches in refractive index. *J. Microsc.* 169:391–405.
31. Jacobsen, H., and S. W. Hell. 1995. Effect of the specimen refractive index on the imaging of a confocal fluorescence microscope employing high aperture oil immersion lenses. *Bioimaging.* 3:39–47.
32. Saxton, M. J. 1997. Single-particle tracking: the distribution of diffusion coefficients. *Biophys. J.* 72:1744–1753.
33. Dunne, P. D., R. A. Fernandes, …, D. Klenerman. 2009. DySCo: quantitating associations of membrane proteins using two-color single-molecule tracking. *Biophys. J.* 97:L5–L7.

34. Ruprecht, V., M. Brameshuber, and G. J. Schütz. 2010. Two-color single molecule tracking combined with photobleaching for the detection of rare molecular interactions in fluid biomembranes. *Soft Matter.* 6:568–581.

35. Kusumi, A., T. A. Tsunoyama, …, T. K. Fujiwara. 2014. Tracking single molecules at work in living cells. *Nat. Chem. Biol.* 10:524–532.

36. Chen, J., Z. Zhang, …, Z. Liu. 2014. Single-molecule dynamics of enhanceosome assembly in embryonic stem cells. *Cell.* 156:1274–1285.

37. Greiss, F., M. Deligiannaki, …, D. Braun. 2016. Single-molecule imaging in living drosophila embryos with reflected light-sheet microscopy. *Biophys. J.* 110:939–946.

38. Grimm, J. B., B. P. English, …, L. D. Lavis. 2015. A general method to improve fluorophores for live-cell and single-molecule microscopy. *Nat. Methods.* 12:244–250, 3, 250.

39. Herbert, A. Single Molecule Light Microscopy ImageJ Plugins. www.sussex.ac.uk/gdsc/intranet/microscopy/imagej/smlm_plugins. Accessed 2014.

40. Lew, M. D., A. R. von Diezmann, and W. E. Moerner. 2013. Easy-DHPSF open-source software for three-dimensional localization of single molecules with precision beyond the optical diffraction limit. *Protoc. Exch.* 2013:026.

41. Kazhdan, M., M. Bolitho, and H. Hoppe. 2006. Poisson surface reconstruction. *SGP '06 Proc. Symp. Geometry Processing, Cagliari, Italy.* 61–70.

42. Kazhdan, M., and H. Hoppe. 2013. Screened poisson surface reconstruction. *ACM Trans. Graph.* 32:1–13.

43. Weimann, L., K. A. Ganzinger, …, D. Klenerman. 2013. A quantitative comparison of single-dye tracking analysis tools using Monte Carlo simulations. *PLoS One.* 8:e64287.

44. Qian, H., M. P. Sheetz, and E. L. Elson. 1991. Single particle tracking. Analysis of diffusion and flow in two-dimensional systems. *Biophys. J.* 60:910–921.

45. Brown, A. C. N., S. Oddos, …, D. M. Davis. 2011. Remodelling of cortical actin where lytic granules dock at natural killer cell immune synapses revealed by super-resolution microscopy. *PLoS Biol.* 9:e1001152.

46. Pancer, Z., and M. D. Cooper. 2006. The evolution of adaptive immunity. *Annu. Rev. Immunol.* 24:497–518.

47. Garboczi, D. N., P. Ghosh, …, D. C. Wiley. 1996. Structure of the complex between human T-cell receptor, viral peptide and HLA-A2. *Nature.* 384:134–141.

48. van der Merwe, P. A., and O. Dushek. 2011. Mechanisms for T cell receptor triggering. *Nat. Rev. Immunol.* 11:47–55.

49. O'Shaughnessy, A., and B. Hendrich. 2013. CHD4 in the DNA-damage response and cell cycle progression: not so NuRDy now. *Biochem. Soc. Trans.* 41:777–782.

50. Zhang, W., A. Aubert, …, E. D. Laue. 2016. The nucleosome remodeling and deacetylase complex NuRD is built from preformed catalytically active sub-modules. *J. Mol. Biol.* 428:2931–2942.

51. Tokunaga, M., N. Imamoto, and K. Sakata-Sogawa. 2008. Highly inclined thin illumination enables clear single-molecule imaging in cells. *Nat. Methods.* 5:159–161.

52. Huang, F., G. Sirinakis, …, J. Bewersdorf. 2016. Ultra-high resolution 3D imaging of whole cells. *Cell.* 166:1028–1040.

53. Pernis, B. 1985. Internalization of lymphocyte membrane components. *Immunol. Today.* 6:45–49.

54. Legant, W. R., L. Shao, …, E. Betzig. 2016. High-density three-dimensional localization microscopy across large volumes. *Nat. Methods.* 13:359–365.

55. Monks, C. R. F., B. A. Freiberg, …, A. Kupfer. 1998. Three-dimensional segregation of supramolecular activation clusters in T cells. *Nature.* 395:82–86.

56. Grakoui, A. 1999. The immunological synapse: a molecular machine controlling T cell activation. *Science.* 285:221–227.

57. Finetti, F., A. Onnis, and C. T. Baldari. 2015. Regulation of vesicular traffic at the T cell immune synapse: lessons from the primary cilium. *Traffic.* 16:241–249.

58. Choudhuri, K., J. Llodrá, …, M. L. Dustin. 2014. Polarized release of T-cell-receptor-enriched microvesicles at the immunological synapse. *Nature.* 507:118–123.

59. James, J. R., S. S. White, …, D. Klenerman. 2007. Single-molecule level analysis of the subunit composition of the T cell receptor on live T cells. *Proc. Natl. Acad. Sci. USA.* 104:17662–17667.

60. Adler, J., A. I. Shevchuk, …, I. Parmryd. 2010. Plasma membrane topography and interpretation of single-particle tracks. *Nat. Methods.* 7:170–171.

61. Jung, Y., I. Riven, …, G. Haran. 2016. Three-dimensional localization of T-cell receptors in relation to microvilli using a combination of super-resolution microscopies. *Proc. Natl. Acad. Sci. USA.* 113:E5916–E5924.

62. Mege, J.-L., C. Capo, …, P. Bongrand. 1986. Quantification of cell surface roughness; a method for studying cell mechanical and adhesive properties. *J. Theor. Biol.* 119:147–160.

63. Zhu, D.-M., M. L. Dustin, …, D. E. Golan. 2007. Analysis of two-dimensional dissociation constant of laterally mobile cell adhesion molecules. *Biophys. J.* 92:1022–1034.

64. Jönsson, P., J. H. Southcombe, …, D. Klenerman. 2016. Remarkably low affinity of CD4/peptide-major histocompatibility complex class II protein interactions. *Proc. Natl. Acad. Sci. USA.* 113:5682–5687.

65. Sage, P. T., L. M. Varghese, …, C. V. Carman. 2012. Antigen recognition is facilitated by invadosome-like protrusions formed by memory/effector T cells. *J. Immunol.* 188:3686–3699.

66. Lee, S. A., A. Ponjavic, …, J. S. Biteen. 2016. Nanoscopic cellular imaging: confinement broadens understanding. *ACS Nano.* 10:8143–8153.

67. Ritter, J. G., R. Veith, …, U. Kubitscheck. 2010. Light sheet microscopy for single molecule tracking in living tissue. *PLoS One.* 5:e11639.

68. Yu, J., B. Cao, …, H. Niu. 2014. Improved localization accuracy in double-helix point spread function super-resolution fluorescence microscopy using selective-plane illumination. *Proc. SPIE.* 92300N. http://dx.doi.org/10.1117/12.2068945.

69. Hu, Y. S., H. Cang, and B. F. Lillemeier. 2016. Superresolution imaging reveals nanometer- and micrometer-scale spatial distributions of T-cell receptors in lymph nodes. *Proc. Natl. Acad. Sci. USA.* 113:7201–7206.

70. Ricci, M. A., C. Manzo, …, M. P. Cosma. 2015. Chromatin fibers are formed by heterogeneous groups of nucleosomes in vivo. *Cell.* 160:1145–1158.

71. El Beheiry, M., and M. Dahan. 2013. ViSP: representing single-particle localizations in three dimensions. *Nat. Methods.* 10:689–690.

Biophysical Journal, Volume *112*

Supplemental Information

Three-Dimensional Super-Resolution in Eukaryotic Cells Using the Double-Helix Point Spread Function

Alexander R. Carr, Aleks Ponjavic, Srinjan Basu, James McColl, Ana Mafalda Santos, Simon Davis, Ernest D. Laue, David Klenerman, and Steven F. Lee

Supplementary Information

1. Imaging above the coverslip with water and oil immersion objective lenses

Using a water immersion objective lens reduces aberration of the DHPSF when imaging above the coverslip if $n_{media} \approx n_{water} \approx 1.33$. DHPSFs from different depths above the coverslip and their rotation are shown in Figure S1. 100 µL of a ~$3.6 \cdot 10^{8}$ particles/mL solution of fluorescent beads (TetraSpeck Microspheres, 0.1 µm, ThermoFisher) in 1% agarose solution ($n \approx n_{water} \approx 1.33$) was imaged across a depth of 50 µm. Significant deterioration of the DHPSF is seen when imaging with an oil immersion objective lens (Plan Apo TIRF 60×, Nikon), whereas no significant deterioration is seen when imaging with a water immersion objective lens (Plan Apo VC 60× H, Nikon).

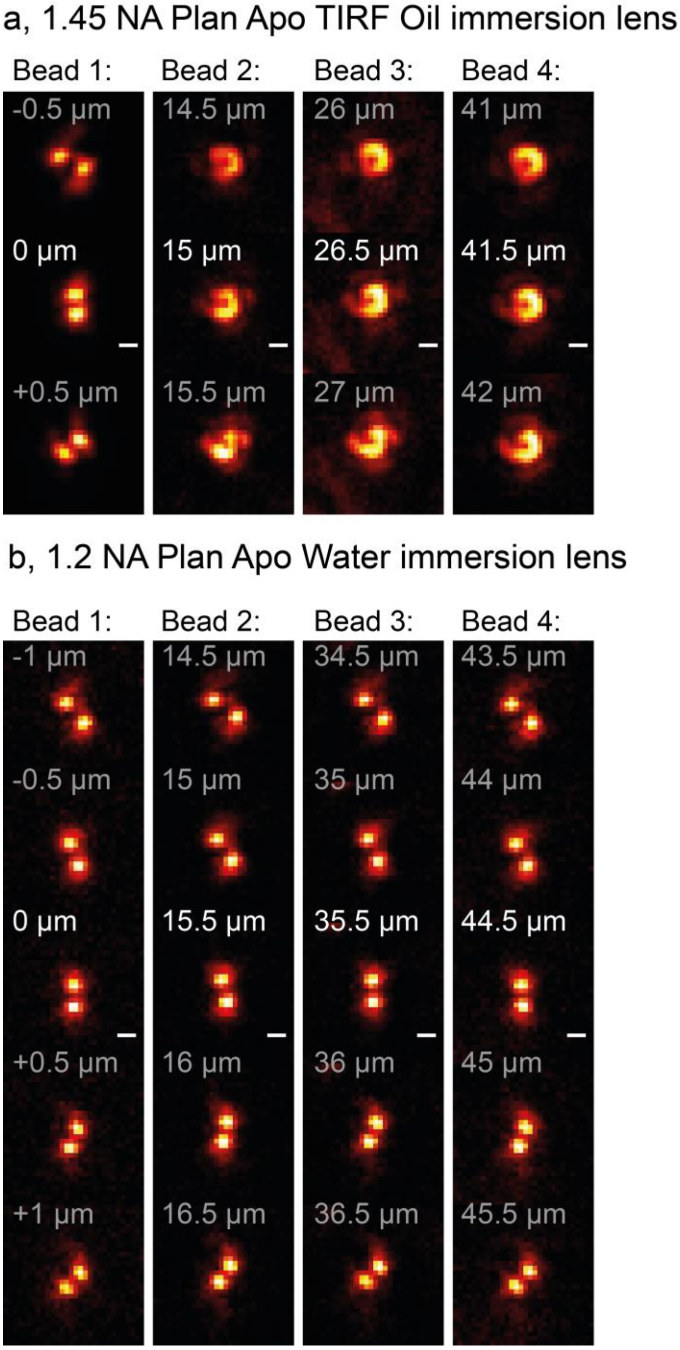

SI Figure S1 Imaging fluorescent beads with suspended in 1% agarose with the DHPSF above the coverslip. Labels represent the distance above the coverslip for each image plane. Scale bars are 1 µm **(a)** Images of 4 different fluorescent beads captured with an oil immersion objective at different distances above the coverslip. Significant deformation of the DHPSF can be seen away from the coverslip surface. **(b)** Images of 4 different fluorescent beads captured with a water immersion objective. No significant deformation of the DHPSF can be observed even up to 50 µm above the surface.

2. DHPSF aberration as a function of axial position of focal plane

The effect of the axial position of the focal plane on systematic localisation errors induced by aberration was investigated by recording DHPSF calibration stacks at three different axial positions of the focal plane. Three calibration stacks were recorded, at roughly the same area of the field of view, at the coverslip surface, 15 µm above and 30 µm above. The maximum axial depth of the focal plane in all presented experiments was ~15 µm.

Tetraspeck fluorescent beads were suspended in 1% Pytagel and imaged at 30ms exposure. Each bead was aligned to be in the focal plane by eye before scanning the objective in 50 nm steps through a distance of 3.5 µm (starting 1.75 µm below the initial plane and moving up). 10 images were recorded at each step. Easy DHPSF was then used to create calibration curves for each bead. There was no obvious difference between curves recorded above the coverslip surface and those at at 15 µm or 30 µm, with all curves overlapping (Figure S2a).

The total 3D Cartesian error between calibration scans was investigated using easy DHPSF to localise the position of the fluorescent beads in these calibration scans. One of the three beads located on the surface was used the calibration stack in easy DHPSF in order to calibrate thresholds for fitting the remaining calibration stacks.

The total Cartesian error between calibration stacks was determined at each 50 nm axial step for each bead using the mean position of localisations from the respective image plane in x, y and z. This was evaluated with the equation: $r = \sqrt{r_x^2 + r_y^2 + r_z^2}$, where r is the total Cartesian error and $r_{x,y,z}$ is the mean displacement in x, y and z dimensions respectively. The x, y and z position of the central axial step for each bead was considered as the origin in order to calculate displacement (the 50 nm z steps expected between successive axial planes were subtracted from the z displacement).

Significant localisation errors increasing towards the periphery of the depth of field were observed reaching up to ~100 nm at the extremes, however, no difference between calibration curves recorded at different heights above the coverslip were seen. This indicates that the cause of this error source is not aberration induced by imaging away from the cover slip. These errors are small compared to the distances considered in quantitative analysis and SI section 9 ‘Benchmarking of 3D MSD analysis’ demonstrates that diffusion measurements are robust even at low localisation precision (corresponding to low photon number in the figure) for the values presented.

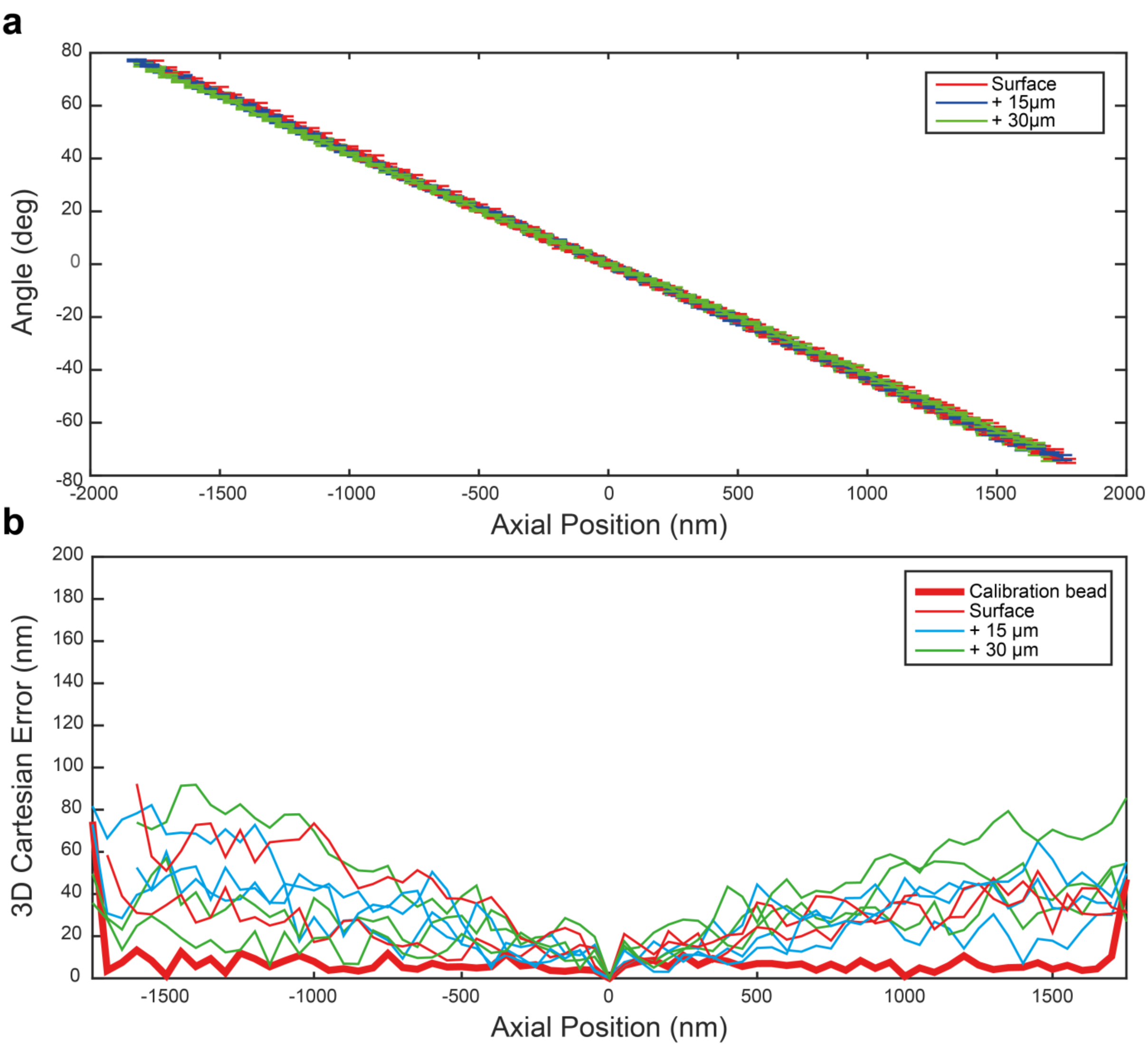

Figure S2 The effect of focal plane axial depth on 3D Cartesian error. Tetraspeck fluorescent beads were imaged at different depths above the coverslip surface in 50 nm axial steps. **(a)** Calibration curves of angle between DHPSF lobes as a function of axial depth for three different fluorescent beads at each axial depth; at the surface (red), 15 μm above (blue) and 30 μm above (green). The majority of the curves cannot be seen due to overlap. **(b)** Total Cartesian error as a function of axial depth for three different fluorescent beads at each axial depth; at the surface (red), 15 μm above (blue) and 30 μm above (green). The bead used for calibration in the easy DHPSF reconstruction is highlighted (thick red line).

3. **DHPSF microscope schematic**

A bespoke DHPSF microscope was built as described in the main text and as shown in Figure S3.

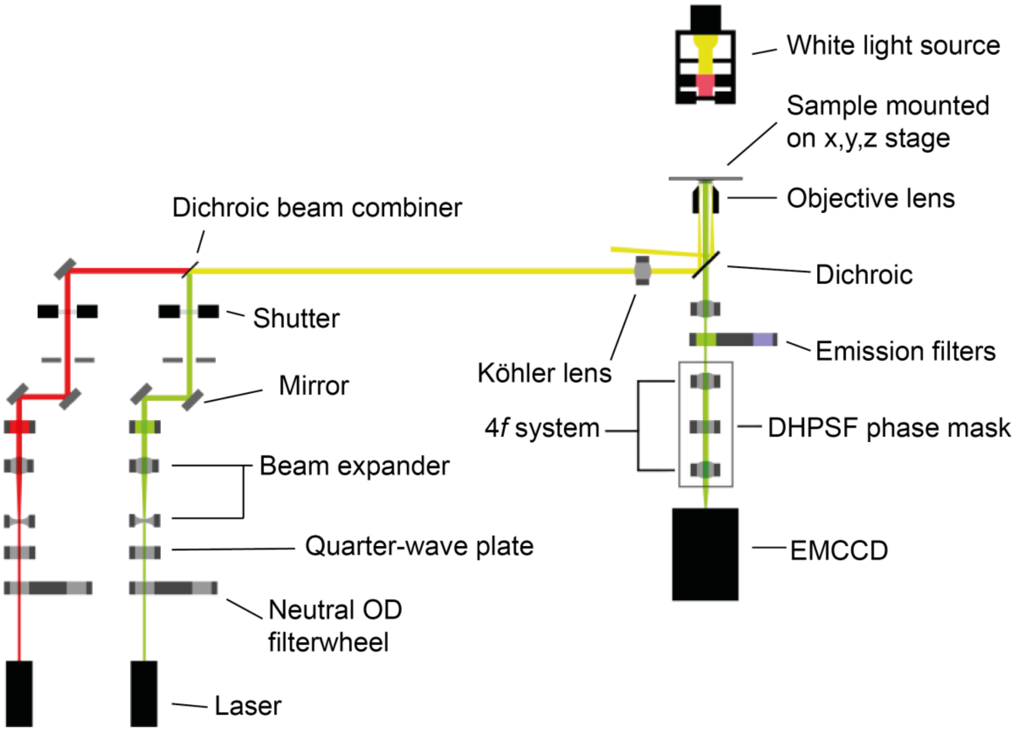

Figure S3 DHPSF microscope schematic. A combined beam path of excitation (561 nm) and activation (405 nm) lasers is shown.

4. Typical detected photon ranges for experimental data

The number of photons detected was given by the sum of the integrated signal from a Gaussian fit to each DHPSF lobe. The signal was given by the total ADU signal from the camera divided by the total camera gain (~38 ADU/photon in the presented experiments).

A histogram was created containing the number of detected photons in each localisation for each different fluorophore and exposure length (Figure S4), which is summarised in SI table 1. Error limits for the corresponding localisation precision were estimated from the distribution of points in Figure 1a.

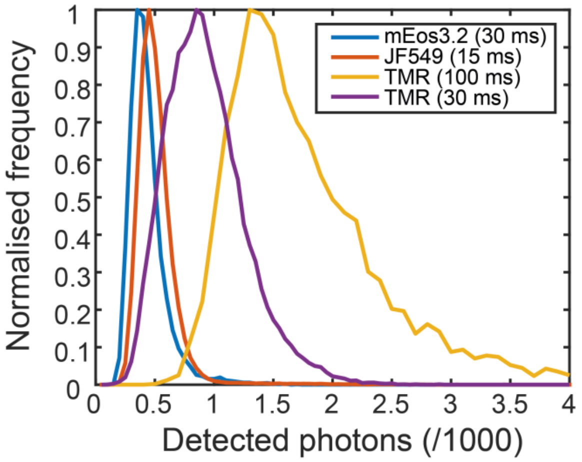

Figure S4 Number of photons detected. Histograms of the number of photons detected during typical imaging in presented data. mEos3.2 (30 ms) and TMR (100ms) were used in localisation imaging whereas JF549 (15 ms) and TMR (30ms) were used in SPT.

Fluorophore	Exposure (ms)	Peak Detected Photons	5th percentile	95th percentile	Back-ground photons per pixel	Peak x,y Localisation precision (nm)	Peak z localisation precision (nm)
JF549	15	450	300	700	6.7 ± 0.5	24 ± 2	55 ± 5
mEos3.2	30	350	250	700	5.4 ± 0.1	26 ± 2	59 ± 5
TMR	30	850	450	1550	10.2 ± 0.4	19 ± 2	43 ± 5
TMR	100	1350	950	3000	24.3 ± 0.4	15 ± 2	33 ± 5

Table S1 Summary of detected photon values from SI Figure 4 with number of background photons per pixel per frame from within cell volumes and lateral and axial localisation precisions.

5. Optimal overlapping of imaging planes for flat localisation density

In order to achieve the most flat localisation density across volumes spanning multiple imaging planes the optimal offset between planes was experimentally determined to be ~3 μm or ~75% of the working depth of field.

First, the localisation density of a single plane was measured by imaging the membrane protein TCR via HaloTag-TMR in the central plane of 5 T cells. Localisations were sorted into 500 nm bins axially and their relative density calculated (Figure S5a). The localisation density correlated well to the axial intensity distribution of the DHPSF as output from easy-DHPSF (Figure S5b), which is expected as the ability to localise PSFs is directly related to the detected photons.

An approximate distribution removing noise in the centre of the density distribution (shown in Figure S5a) was used as example image planes to determine the optimal plane offset. Two identical approximate distributions were plotted with one axially offset by between 2 μm and 5 μm in 500 nm steps. For each offset, the mean localisation density was calculated from a 4 μm thick section including the interface between imaging planes from the sum of the two distributions:

$$\langle density_{total} \rangle = \langle density_1 + density_2 \rangle$$

An overlap of 3-3.5 μm was found to yield the most-flat localisation density across the interface between two imaging planes (highlighted area in Figure S5c).

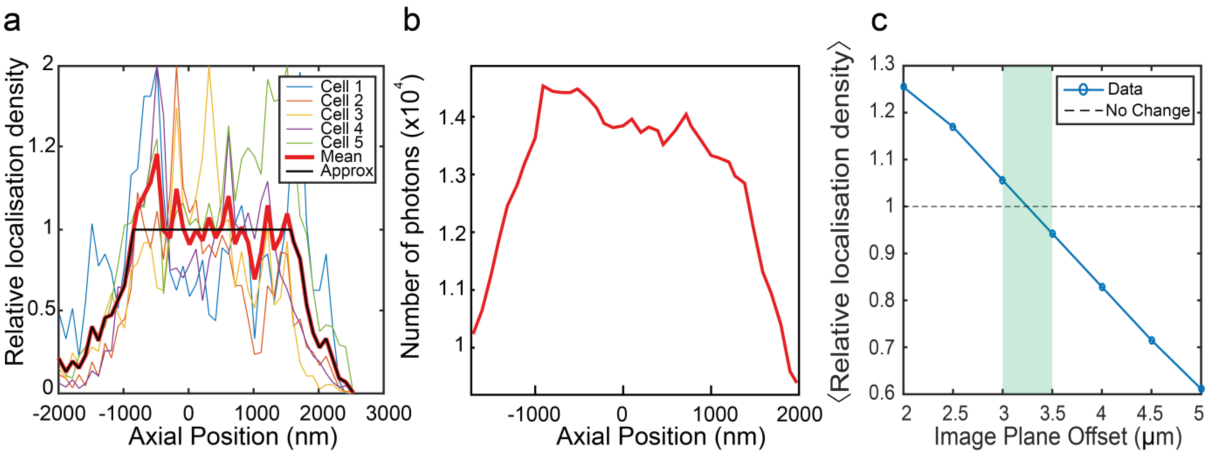

Figure S5 Optimal overlapping of successive image planes. **(a)** Localisation density across a single image plane from 5 cells with average (thick red) and approximate (black) distributions. **(b)** Number of photons detected from a single PSF across the depth of field during DHPSF calibration. **(c)** Relative localisation density across a 4 μm thick section at the interface of two successive simulated imaging planes. The highlighted green area represents the optimal range of plane offset to achieve the most flat localisation density across large samples.

6. Focal drift

The drift of the system was measured by imaging Tetraspeck beads suspended in 1% Phytagel (P8169, Sigma). Beads were imaged in the centre of the field of view for 12,000 frames at 100ms exposure. The mean position of the beads in three dimensions from successive sections of 100 frames was used to track drift (Figure S6). This was repeated 7 times. A linear fit was used to estimate the expected drift as a function of time. These measurements represent the typical drift of the imaging system used to conduct the experiments presented in the manuscript.

The drift was found to follow the equation: $\Delta r = (0.0044 \pm 0.0003)N + (1.3 \pm 1.8)$. Where Δr is the Cartesian drift distance and N is the number of frames (at 100ms exposure). Using this fit, the 20,000 frame acquisitions presented in the manuscript would expect total drift of 89 ± 8 nm by the end of the experiment. However, the majority of the localisations will be recorded with less drift as the density of fluorescence events decays over the course of the experiment.

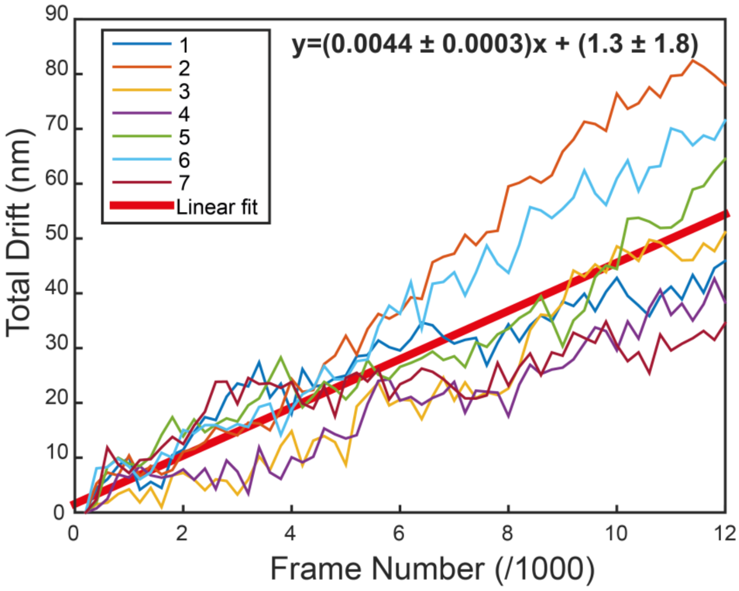

Figure S6 Drift of microscope platform over time. The Cartesian distance drifted as a function of frame number (at 100ms exposure) is plotted for 7 repeats. A linear fit (thick red line) was used to estimate the expected drift at different time points.

7. Lateral offset as a function of axial distance into sample

The lateral position of successive imaging planes relative to each other was examined by measuring the displacement of Tetraspeck beads suspended in 1% Phytagel (P8169, Sigma). Beads were imaged at two image planes, first near the coverslip and then 2.5 µm above this so that the same bead was still in the depth of field. 100 frames were acquired at each image plane before moving to the next. This was repeated 60 times, as in the experiments presented in Figures 2 and 3, acquiring a total of 12,000 frames for each bead.

The average position of each bead was compared between successive image planes for each repeat using with the Cartesian distance between the two centres plotted on a histogram (Figure S7). This histogram was fit with a 1-dimensional Gaussian to extract the centre position and width.

It was determined that moving an axial distance of +2.5 µm resulted in a 9.1 ± 4.4 nm lateral shift. This results in a lateral shift of ~45.5 nm across the presented imaging volumes (~12.5 µm). This is comparable to the localisation precision of the DHPSF and is small compared to the accuracy of the fitted mesh and to the quantitative values considered in the whole-cell scanning experiments, which look at the distance between protein molecules across the whole ~12.5 µm thick volume (see Figure S13, the statistical fluctuations of the presented distances are on the order of ~1-2 µm).

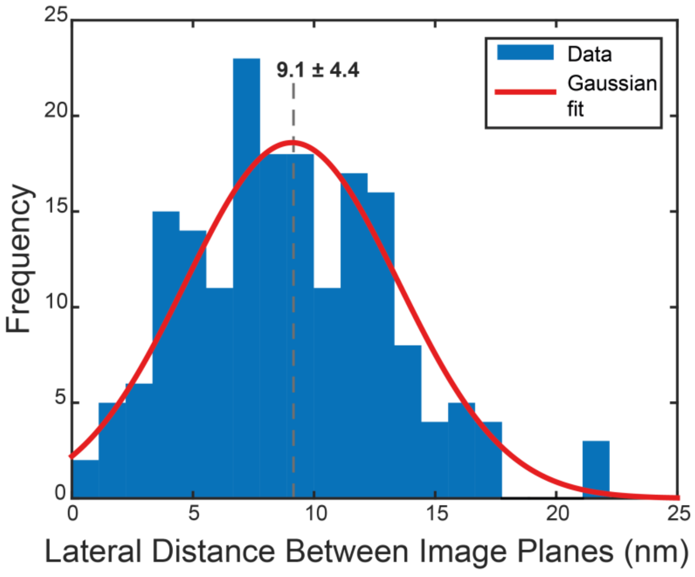

Figure S7 Lateral offset between successive image planes. A histogram of the measured Cartesian lateral offset between successive image planes separated by 2.5 µm axially. A1D Gaussian function was fit to the data (red line) in order to extract the centre position and width, which are labelled.

8. Mesh fitting to localisation data

Using a standard method and functions included in Meshlab (http://meshlab.sourceforge.net) 3D localisation data was converted into an object mesh via the following steps:

1. Import localisation data as point could into meshlab from '.xyz' file type
2. Create normals to each localisation
    a. The built in 'Compute Normals for Point Set' function in the 'Normals, Curvatures and Orientation' tab of the 'Filters' menu was used. An input number of neighbours ranging between 50 and 200 depending on localisation density.
3. Reconstruct a surface mesh using the Poisson surface approach
    a. The built in 'Surface Reconstruction: Poisson' function in the 'Remeshing, Simplification and Reconstruction' tab of the 'Filters' menu was used. Typically an 'Octree Depth' of 10, a 'Solver Divide' of 6, a 'Samples per Node' of 1 and a 'Surface Offsetting' of 1 were selected but these parameters were adjusted slightly between datasets for best results.
4. Uniformly sample the mesh
    a. The built in 'Uniform Mesh Resampling' function in the 'Remeshing, Simplification and Reconstruction' tab of the 'Filters' menu was used

Figure S8 shows a fitted mesh to the localisation data presented in Figure 2 in the main text. Both the mesh and the individual points are shown with an inset zooming in to a 1.5 µm thick section showing how the mesh follows the approximate distribution of the localisations. The localisation and mesh datasets are provided as additional supplementary files.

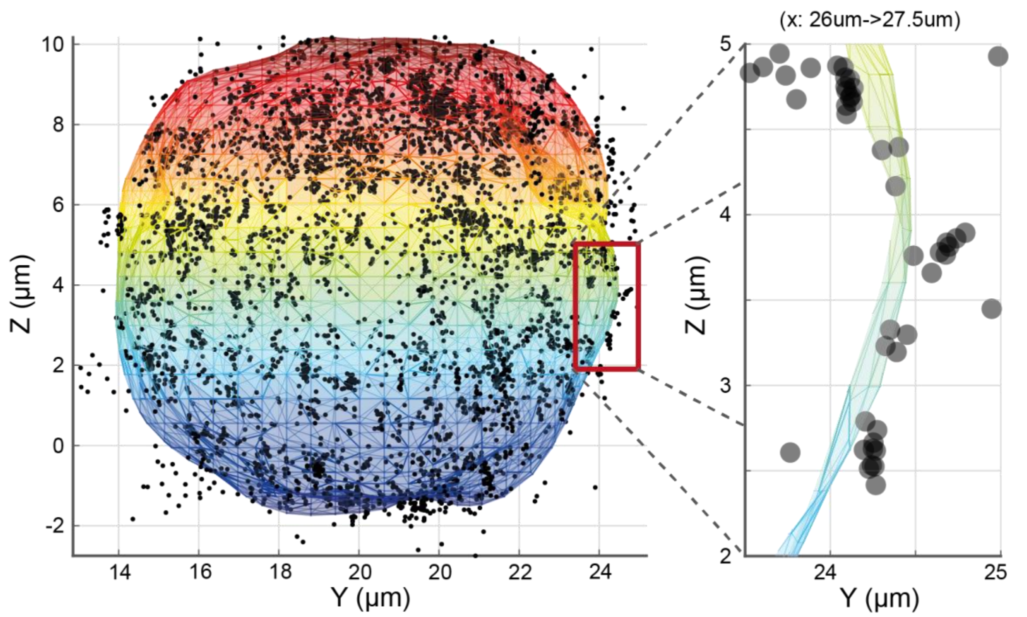

Figure S8 Fitting a mesh to localisation data. Individual localisations from Figure 2 are represented as dark grey dots. The fitted mesh is coloured from red to blue from top to bottom for visibility. The inset shows a zoom in on a 1.5 µm thick volume.

9. Benchmarking of 3D MSD analysis

In order to verify the MSD written analysis algorithm its performance was tested on 2D and 3D data sets. When analysing 2D data each localisation was assigned an axial position of 0 nm in all cases to satisfy the data format required for the algorithm.

A 2D TIRF dataset of TCR diffusing on the basal surface of 12 T cells on passivating coated glass surfaces was used to benchmark the written algorithm's performance against a previously published 2D MSD analysis code (Weimann et al (1)). Unlike the written algorithm, the previously published code includes a 2D fitting step. Peakfit (2) was used to create a localisation list that was then used as the input for the written algorithm. The overall MSD curves produced from all trajectories and calculated diffusion coefficients are shown in Figure S9a. All points on the MSD curves are within error between the two MSD analysis codes and the calculated diffusion coefficients agree well.

The written algorithm was also benchmarked on simulated 3D datasets across a range of diffusion coefficients. Simulated datasets were created by distributing points randomly in space and moving each point in 3 dimensions by a distance randomly sampled from a normal distribution with a width equal to the one dimensional mean squared displacement for each frame.

$$MSD = \sqrt{2 * D * dt}$$

Where D is the diffusion coefficient being simulated and dt is the simulated time between localisations.

For each frame, an additional movement was added in all the dimensions to simulate the localisation precision of the instrument. This distance was sampled from a normal distribution with width equal to the measured lateral localisation precision in x and y and the axial localisation precision in z at a given intensity (distributions can be seen in Figure 1 in main text).

All simulated trajectories were 16 frames long as this is the minimum number of points a trajectory requires to be considered in the analysis code. This is in order to be able to create 4 msd points for a linear fit as the number of MSD points should not exceed 25% of the total number of points in a trajectory, as stated by Saxton (3).

The recall of the simulated 3D diffusion coefficients was determined from 20 simulated data sets at each simulated diffusion coefficient as defined by:

$$Recall = {}^{D_{calculated}}/_{D_{simulated}}$$

Firstly, the effect of the number of tracks considered on recall was investigated using a typical experimental localisation precision of 25 nm laterally and 50 nm axially and a time step of 30 ms (Figure S9b-c). The mean error in recall was below 2% after 100 trajectories are considered and the standard deviation of recall was below 5% after 200 trajectories are considered for all simulated diffusion coefficients. This indicates that after 200 trajectories of minimal length the analysis is robust to diffusion coefficients of 0.01 $\mu m^2/s$, with less trajectories required for faster diffusion coefficients.

The effect of localisation precision as determined by the number of detected photons on recall was investigated (Figure S9d-e). 400 tracks were considered with a time step of 30 ms from 20 simulated datasets for each simulated diffusion coefficient. The mean error in recall was below 2% after 100 photons, which is below the sensitivity of the microscope, for all simulated diffusion coefficients. The standard deviation of recall was below 5% for simulated diffusion coefficients of 0.1 $\mu m^2/s$ and higher

at all considered detected photon numbers. For a simulated diffusion coefficient of 0.01 μm^2/s, ~500 detected photons results in a standard deviation of recall <5%. This indicates that, at all experimentally possible detected photons numbers, analysing 400 trajectories of minimal length the analysis is robust at diffusion coefficients >=0.1 μm^2/s. To analyse slower diffusion coefficients a mean detected photon number of ~>500 is required. These numbers all fit with experimental conditions that were used in all data presented in the main text.

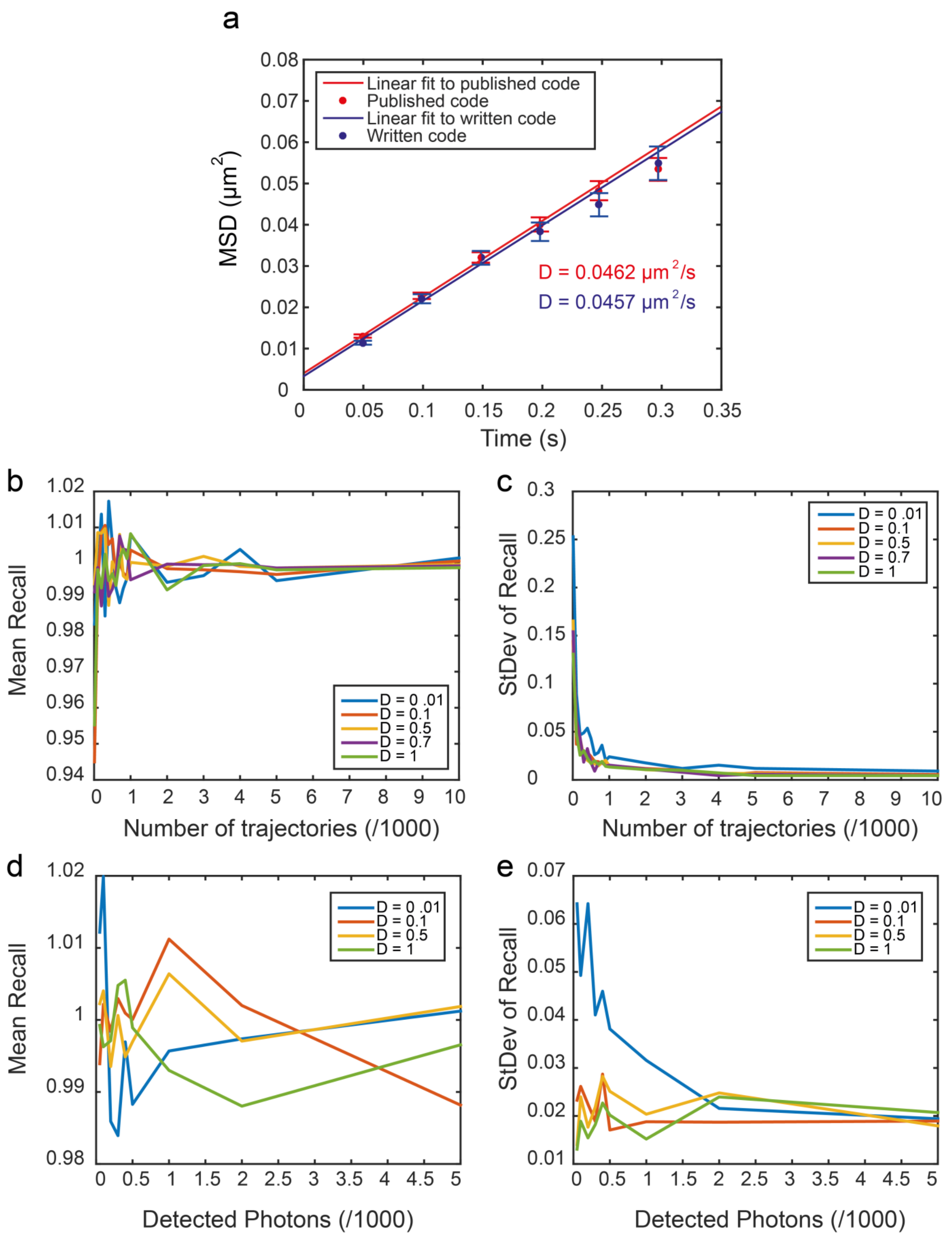

Figure S9 Benchmarking of written MSD analysis algorithm. **(a)** Comparing analysis of a 2D data set to a previously published MSD analysis algorithm. **(b)** Mean recall in diffusion coefficient from 20 simulated datasets at a range of diffusion coefficients as a function of the number of trajectories considered. **(c)** Standard deviation in recall of simulated datasets from (b). **(d)** Mean recall in diffusion coefficient from 20 simulated datasets at a range of diffusion coefficients as a function of detected photons. **(e)** Standard deviation in recall of simulated datasets from (d).

10. Identifying bound trajectories by R^2 analysis of MSD linear fit

In order to separate bound and unbound trajectories during MSD analysis individual MSD curves were compared to a linear fit to the first 4 points. Trajectories whose MSD curve did not fit well to a linear fit, by measure of R^2 value, were considered to be bound. Bound and unbound trajectories could then be analysed separately. In order to determine the threshold R^2 value to best separate the populations simulated data was analysed. As described above, 600 trajectories were simulated using diffusion coefficients of 0 μm²/s (bound), 0.1 μm²/s (unbound, typical membrane protein) and 0.7 μm²/s (unbound, typical of CHD4), each with axial and lateral localisation precisions centred at expected values (see Table S1). The first 4 points of the MSD curve of each trajectory was fit to a straight line and the R^2 value recorded. A cumulative histogram for the R^2 values of each population was created (Figure S10). An R^2 value of 0.85 was determined to result in the most accurate identification of diffusion state, identifying >90% of unbound and bound trajectories correctly.

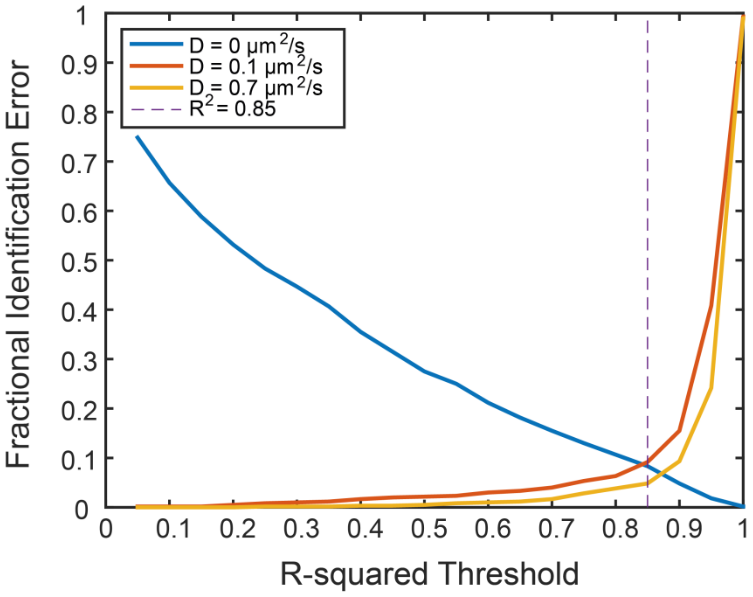

Figure S10 Identifying bound vs unbound populations by R^2 value of linear fit. The fraction of simulated tracks from three diffusion populations that were misidentified as a function of the threshold value chosen. The dashed line is plotted at R^2 = 0.85.

11. Whole cell image data

No significant systematic differences were observed between imaged T cells as shown in SI Figure 11. SI Figure 11 shows additional whole-cell reconstructions of fixed Jurkat T cells expressing CD28-mEos3.2 and TCR-HaloTag-TMR. Localisations are rendered with 200nm localisation precision for visibility.

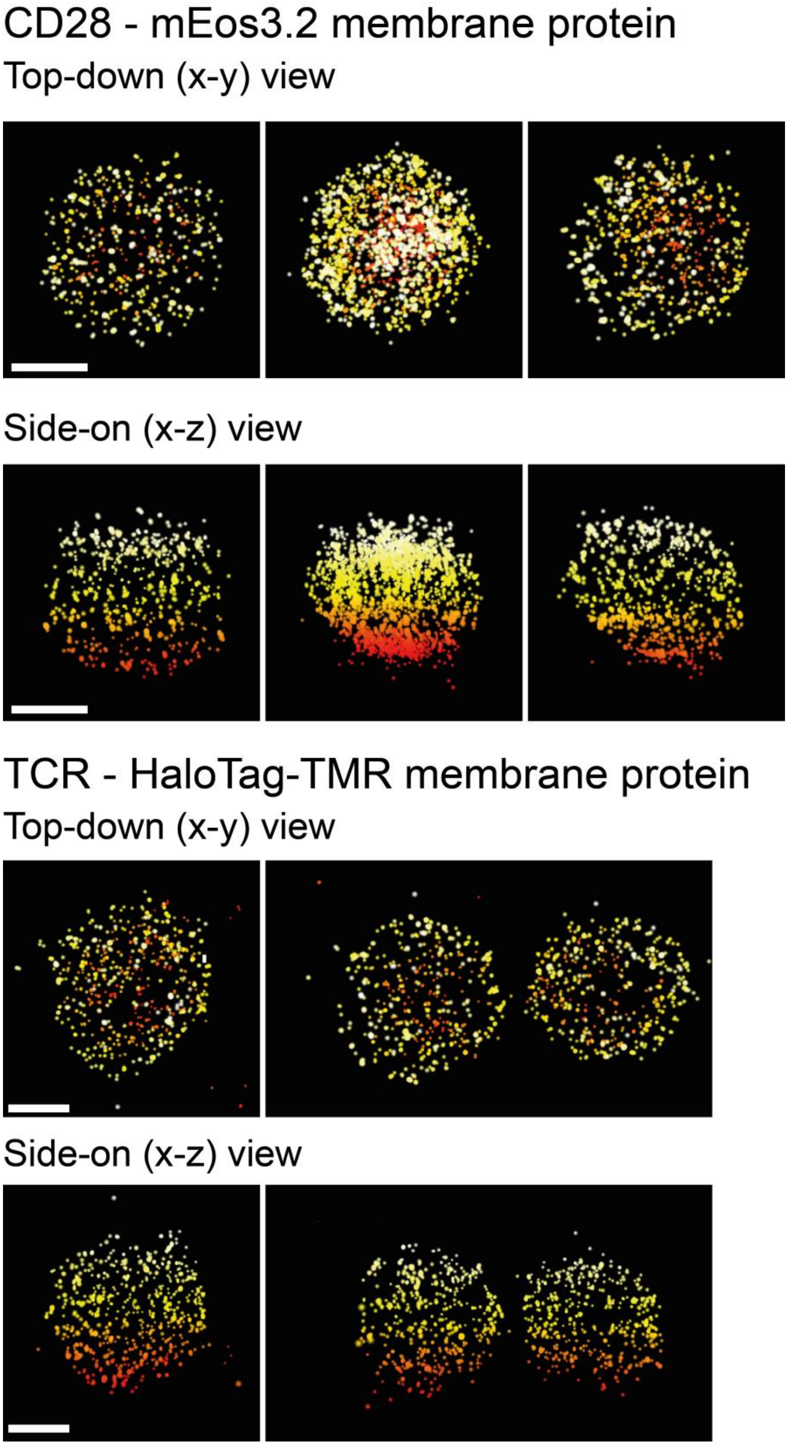

Figure S11 Additional reconstructions of whole-cell super-resolution localisation data of membrane proteins. Side-on and top-down views are shown of three cells expressing CD28-mEos3.2 and three cells expressing TCR-HaloTag-TMR. Localisations are rendered with 200nm precision and scale bars are 5 μm.

12. Nearest neighbour distance of CD28 localisation data

After filtering the localisation data presented in Figure 2a for repeat localisations of the same fluorophore (a radius of 1200 nm and an interval of 3s), the nearest neighbour distance between CD28 molecules was calculated and plotted in a cumulative histogram (Figure S12). 27% of CD28 molecules were revealed to be less than 250 nm apart and 63% were less than 500 nm apart, highlighting the necessity for super-resolution techniques compared to confocal microscopy or other diffraction limited techniques.

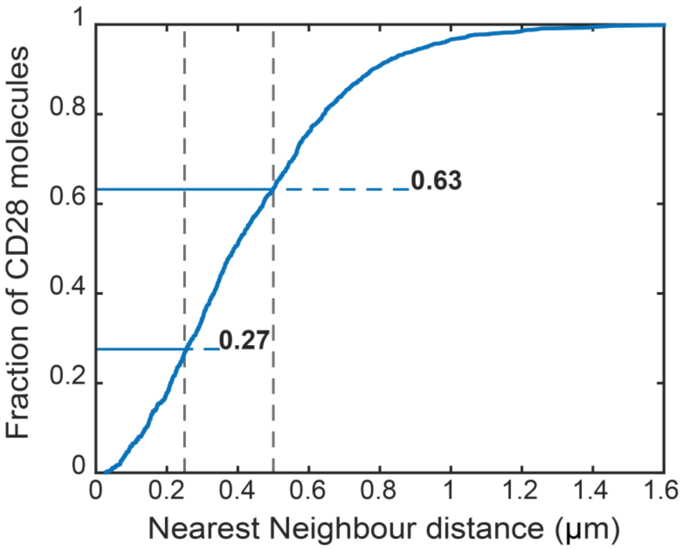

Figure S12 Cumulative histogram of the nearest neighbour distance between CD28 molecules. ~3,000 CD28 molecules were recorded. The two dashed lines indicate a distance of 250 nm and 500 nm. The corresponding fractions at these two distances have been labelled.

13. Correcting for shape with cell-specific models

The distribution of inter-protein distance is highly dependent on the shape of the cell itself. Mostly round cells have an almost uniform distribution due to their symmetry (Figure S13b-c), while other morphologies result in a non-trivial distribution (Figure S13d). In order to correct for cell shape, a model distribution of inter-protein distances was created by randomly sampling fitted meshes. A number of vertices equal to the number of localisations were randomly sampled from vertices on the mesh, 1000 times. Each time the points were randomly translated by 0-250 nm in all three dimensions in order to determine error limits. The difference between the inter-protein distance analysis of the localisation data and model data allows for the effects of shape to be ignored (Figure S13e-g), providing insight into other biological changes.

In all three cells, no significant axial dependence of inter-protein distance distribution was seen (Figure S13e-g).

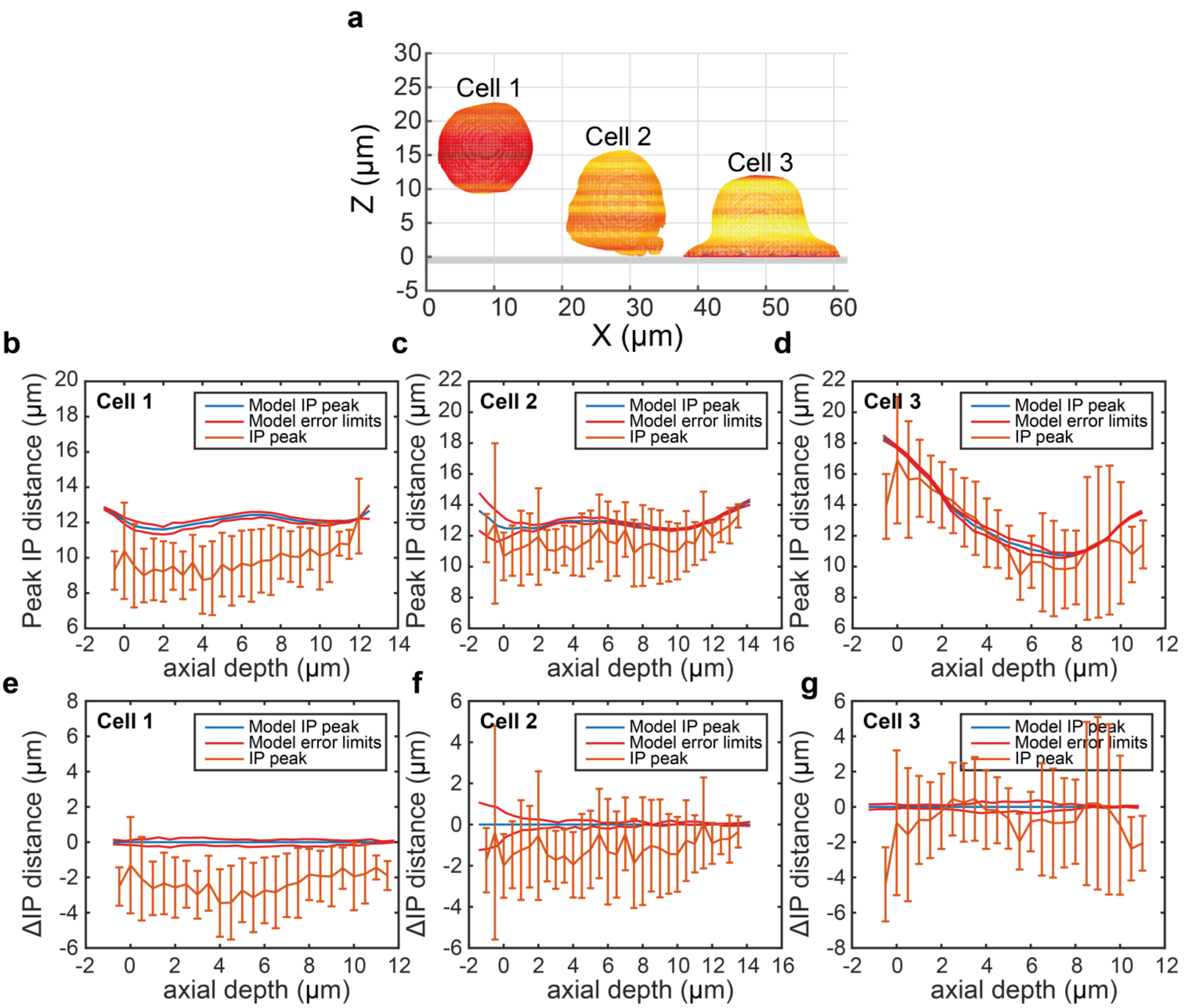

Figure S13 Correcting for the effect of shape on inter-protein (IP) distance distribution. **(a)** Side on cell meshes presented in Figure 3 of the main text. **(b-d)** Peak IP distance of localisation data (orange) and model data (blue) as a function of axial depth from the 3 cells presented in (a). **(e-g)** Shape corrected peak IP distance of localisation data (orange), including error limits determined from model data (red).

14. MSD plot for T cell diffusion data

The ensemble MSD plots for TCR and Zap70 diffusion provide information about the manner of diffusion. The TCR MSD plot fits well to a linear fit indicating free diffusion, while the Zap70 MSD plot drops below the linear fit after the first 4 points indicating a degree of confinement. This is to be expected as TCR is bound to the membrane which forms a closed surface with no edges or obstacles. Zap70 is present intracellularly and thus meets with obstacles such as the outer membrane, nuclear envelope and organelles, partially confining its motion.

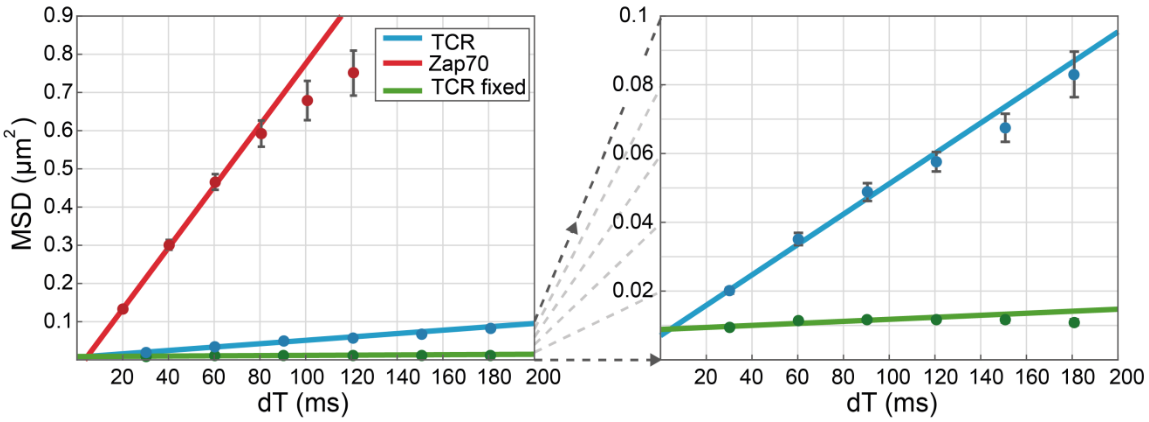

Figure S14 MSD curves for T cell protein diffusion data. (left) MSD plots for Zap70 and TCR live and fixed with linear fits to the first 4 points. (right) Magnification highlighting live and fixed TCR MSD curves.

15. Membrane-bound and cytoplasmic fractions of localised TCR molecules

The smallest distance from each localised protein molecule to the corresponding fitted mesh was used to quantify the fraction of molecules localised in the cytoplasm and on the outer membrane. Duplicate localisations originating from repeat localisation of the same fluorophore were removed by filtering for nearby recorded events in space (<500 nm) and time (<1 second) (results were robust across a large range of thresholding parameters in time and space). The distance between each point and the closest vertex was used to create cumulative histograms for each of the three cells presented (Figure S15).

As the fitted mesh is a weighted approximation of the position of the outer cell membrane, TCR molecules localised on the membrane will not necessarily be found at the fitted mesh. The fraction of molecules differs significantly between the resting cell (0 minutes) and activated cells (5 and 10 minutes) above ~600 nm distances from the fitted meshes.

Analysis of the CD28 localisation data presented in Figure 2 and the corresponding mesh presented in SI Figure 8 was used to determine a threshold separating molecules associated with the outer membrane and intercellular molecules. A significant fraction of CD28 molecules are not associated with the cell's outer membrane but are instead found intracellularly due to protein degradation/creation and the presence of intracellular stores. By analysing only CD28 localisations outside of the mesh volume it is possible to consider a membrane-bound population.

The distance from each CD28 localisation located outside the mesh volume to the nearest vertex was used to create a cumulative histogram. Molecules localised further than 1 μm from the mesh can be considered as located away from the outer membrane of the cell with ~95% certainty (Figure S15a).

For the resting cell, 65% of localised TCR molecules were determined to be on the membrane. For the activated cells (5 and 10 minutes), 72% and 74% of localised TCR molecules were determined to be on the mesh respectively (Figure S15b).

For the resting cell, 30% of TCR molecules were localised intracellularly. For the activated cells (5 and 10 minutes), 17% and 13% of TCR molecules were localised intracellularly respectively (Figure S15c).

For the resting cell, 4% of TCR molecules were localised extracellularly. For the activated cells (5 and 10 minutes), 9% and 12% of TCR molecules were localised extracellularly respectively (Figure S15d. These TCR molecules are more likely associated with long pseudopodia known to be present on the outer membrane of human T cells (4).

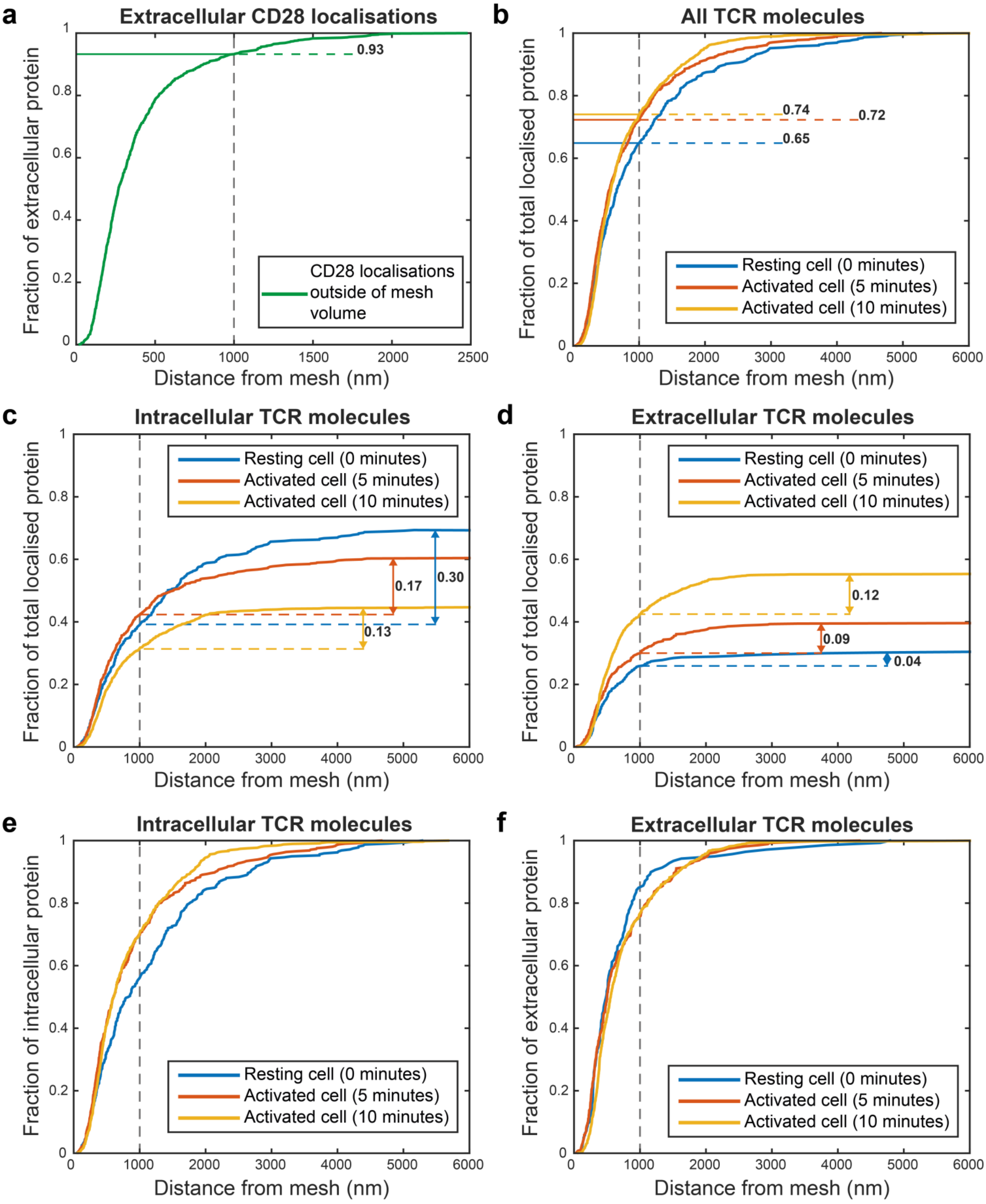

Figure S15 Cumulative histograms of distance from fitted mesh for the cells presented in Figure 2 and 3 in the main text. The grey dashed lines at 1000 nm indicate the threshold used to estimate membrane-bound localisations of TCR molecules. The distribution of the TCR in the resting cell differs significantly from the two activated cells around this threshold. **(a)** Cumulative histogram of the distance from the mesh of CD 28 localisations outside the corresponding mesh volume with the fraction at 1000 nm labelled. **(b)** Cumulative histogram of the distance from the mesh of all TCR molecules to the corresponding mesh with fractions at 1000 nm labelled. **(c)** Cumulative histogram of the distance from the mesh including only TCR molecules localised inside the mesh as a fraction of total number of TCR molecules. The fraction of total localisations further than 1000 nm inside the mesh is labelled for each cell. **(d)** Cumulative histogram of the distance from the mesh including only TCR molecules localised outside the mesh as a fraction of total number of TCR molecules. The fraction of total localisations further than 1000 nm outside the mesh is labelled for each cell. **(e & f)** Cumulative histograms of the distance from the mesh of intracellular and extracellular TCR molecules as a fraction of the number of intracellular and extracellular TCR molecules respectively.

16. 3D diffusion in comparison to 2D models

As described in SI 6, trajectories were simulated diffusing on the top surface of a sphere with a diffusion coefficient of 0.1 $\mu m^2/s$ (Figure S16a) (50 trajectories per sphere, 5 spheres, 20 localisations per track). The trajectories were analysed in 3D using MSD to measure the apparent diffusion coefficient. A 2D projection of the same trajectories was analysed by setting the z-position of each localisation to zero.

The 3D MSD analysis reported a mean diffusion coefficient of 0.103 ± 0.003 $\mu m^2/s$ while the 2D MSD analysis reported a mean diffusion coefficient of 0.078 ± 0.003 $\mu m^2/s$ (Figure S16b). This gives a ratio of 3D to 2D diffusion coefficients of 1.34 ± 0.02, which represents a systematic error in analysing diffusion on a 3D sphere in 2D.

The 3D DHPSF tracking data of TCR presented in Figure 4 was analysed in 2D by setting the z-position of all localisations to zero. The 3D MSD analysis reported a mean diffusion coefficient of 0.110 ± 0.007 $\mu m^2/s$ and the 2D MSD analysis reported a mean diffusion coefficient of 0.064 ± 0.004 $\mu m^2/s$ (Figure S16d). This results in a ratio of 3D to 2D diffusion coefficient of 1.72 ± 0.22, which is considerably larger than expected from diffusion on a sphere. This increase originates from movement away from a spherical surface which can be seen in SI Figure 16c and is likely caused by ruffles and pseudopodia in the T cell outer membrane.

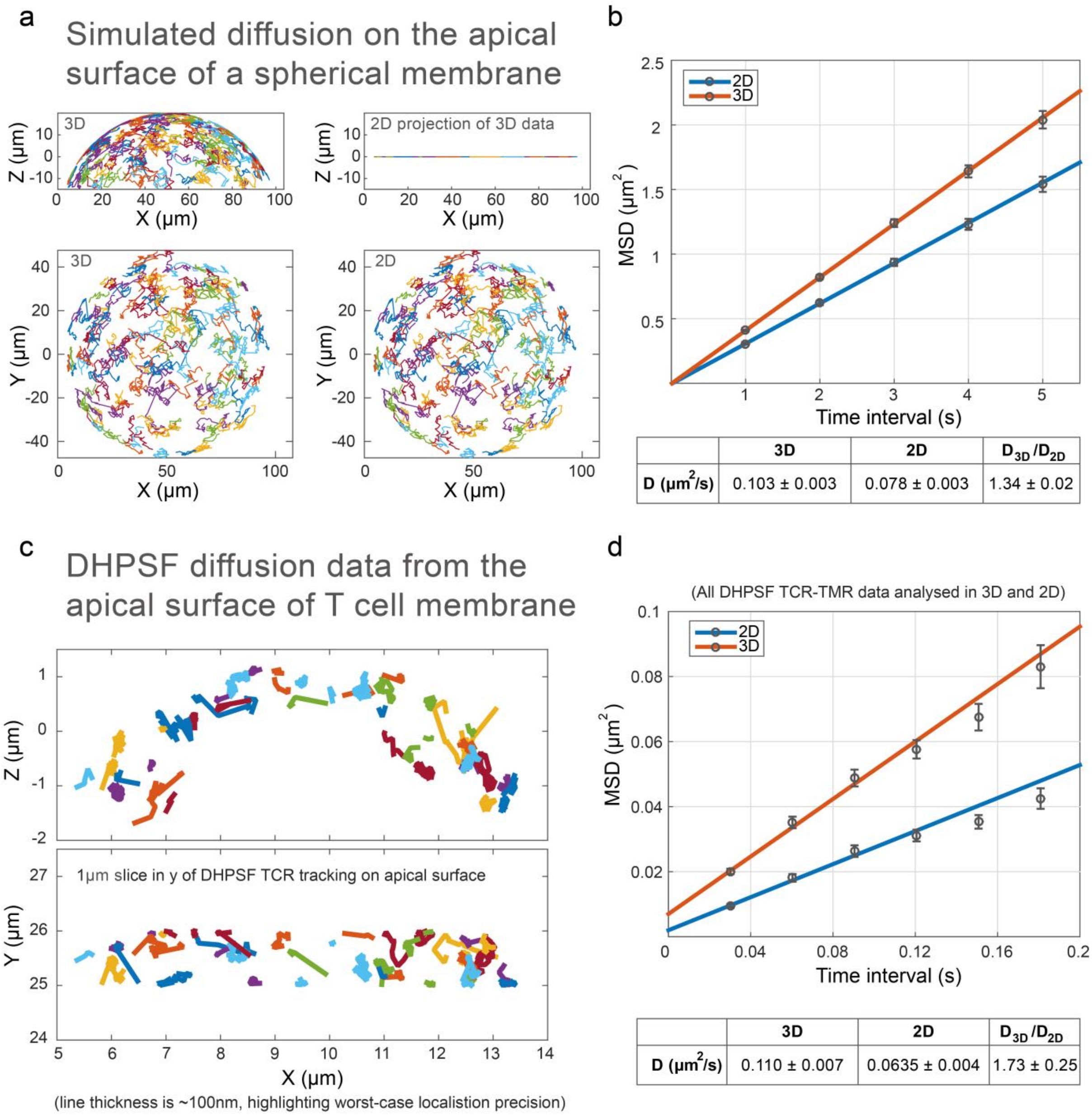

	3D	2D	D_{3D}/D_{2D}
D (μm^2/s)	0.103 ± 0.003	0.078 ± 0.003	1.34 ± 0.02

	3D	2D	D_{3D}/D_{2D}
D (μm^2/s)	0.110 ± 0.007	0.0635 ± 0.004	1.73 ± 0.25

Figure S16 2D analysis of 3D diffusion. **(a)** Top-down and side-on views of simulated 3D trajectories on the top surface of a sphere and the corresponding 2D projection. **(b)** MSD plot for simulated diffusion on a spherical surface analysed in 2D (blue) and 3D (red). **(c)** Top-down and side-on views of TCR trajectories from a 1 μm slice of the apical surface of a T cell. The thickness of trajectories is rendered as ~100 nm to represent the worse-case localisation precision of the DHPSF. Trajectories can be seen to exhibit significant radial movement, away from a model sphere. **(d)** MSD plot for TCR diffusion analysed in 2D (blue) and 3D (red).

Supporting References

1. Weimann, L., K.A. Ganzinger, J. McColl, K.L. Irvine, S.J. Davis, N.J. Gay, C.E. Bryant, and D. Klenerman. 2013. A Quantitative Comparison of Single-Dye Tracking Analysis Tools Using Monte Carlo Simulations. PLoS One. 8: e64287.

2. Herbert, A. Single Molecule Light Microscopy ImageJ Plugins. www.sussex.ac.uk/gdsc/intranet/microscopy/imagej/smlm_plugins. .

3. Saxton, M.J. 1997. Single-particle tracking: the distribution of diffusion coefficients. Biophys. J. 72: 1744–1753.

4. Jung, Y., I. Riven, S.W. Feigelson, E. Kartvelishvily, K. Tohya, M. Miyasaka, R. Alon, and G. Haran. 2016. Three-dimensional localization of T-cell receptors in relation to microvilli using a combination of superresolution microscopies. Proc. Natl. Acad. Sci. U. S. A. 113: E5916–E5924.
